# Rotaxane‐Functionalized Dyes for Charge‐Rectification in *p*‐Type Photoelectrochemical Devices

**DOI:** 10.1002/advs.202306032

**Published:** 2023-12-18

**Authors:** Tessel Bouwens, Tijmen M. A. Bakker, Kaijian Zhu, Annemarie Huijser, Simon Mathew, Joost N. H. Reek

**Affiliations:** ^1^ van ’t Hoff Institute for Molecular Sciences University of Amsterdam Science Park 904 Amsterdam 1098 XH The Netherlands; ^2^ PhotoCatalytic Synthesis Group MESA+ Institute for Nanotechnology University of Twente P.O. Box 217 Enschede 7500 AE The Netherlands

**Keywords:** femtosecond transient absorption, interfacial photoelectrochemistry, *p*‐type dye‐sensitized solar cell, rotaxanes, supramolecular electronics

## Abstract

A supramolecular photovoltaic strategy is applied to enhance power conversion efficiencies (PCE) of photoelectrochemical devices by suppressing electron–hole recombination after photoinduced electron transfer (PET). Here, the author exploit supramolecular localization of the redox mediator—in close proximity to the dye—through a rotaxane topology, reducing electron–hole recombination in *p*‐type dye‐sensitized solar cells (*p*‐DSSCs). Dye P_Rotaxane_ features 1,5‐dioxynaphthalene recognition sites (DNP‐arms) with a mechanically‐interlocked macrocyclic redox mediator naphthalene diimide macrocycle (3‐NDI‐ring), stoppering synthetically via click chemistry. The control molecule P_Stopper_ has stoppered DNP‐arms, preventing rotaxane formation with the 3‐NDI‐ring. Transient absorption and time‐resolved fluorescence spectroscopy studies show ultrafast (211 ± 7 fs and 2.92 ± 0.05 ps) PET from the dye‐moiety of P_Rotaxane_ to its mechanically interlocked 3‐NDI‐ring‐acceptor, slowing down the electron–hole recombination on NiO surfaces compared to the analogue . *p*‐DSSCs employing P_Rotaxane_ (PCE = 0.07%) demonstrate a 30% PCE increase compared to P_Stopper_ (PCE = 0.05%) devices, combining enhancements in both open‐circuit voltages (*V*_OC_ = 0.43 vs 0.36 V) and short‐circuit photocurrent density (*J*_SC_ = −0.39 vs −0.34 mA cm^−2^). Electrochemical impedance spectroscopy shows that P_Rotaxane_ devices exhibit hole lifetimes (*τ*_h_) approaching 1 s, a 16‐fold improvement compared to traditional I^−^/I_3_^−^‐based systems (*τ*_h_ = 50 ms), demonstrating the benefits obtained upon nanoengineering of interfacial dye‐regeneration at the photocathode.

## Graphical abstract

A rotaxane‐dye design is demonstrated to enhance the charge separation in *p*‐type dye‐sensitized solar cells . Supramolecular localization of the redox mediator—in close proximity to the dye—is exploited through a rotaxane‐topology to achieve charge‐rectification. This supramolecular photovoltaic strategy enhances the power conversion efficiency (PCE) of photoelectrochemical devices by suppressing electron–hole recombination after photoinduced electron transfer (PET).

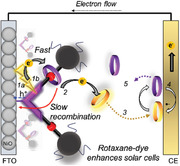

## 1 Introduction

Dyes and pigments featuring rotaxane topologies are known in the scientific community and find application as nanoscale switches in molecular electronics amongst others.^[^ ^1^, ^2^, ^3^, ^4^, ^5^ ^]^ In this work, we investigate a supramolecular photovoltaics approach to rectify charge propagation in solar cells through the use of rotaxane sensitizers.

Supramolecular electronics is a nascent sub‐field of molecular electronics and has the potential to benefit from weak, non‐covalent interactions between the components to (pre)organize their spatial positions to promote charge transport within molecular electronic devices.^[^ ^6^, ^7^, ^8^, ^9^, ^10^, ^11^, ^12^, ^13^, ^14^, ^15^ ^]^ While the development of supramolecular electronics is a thriving field,^[^ ^16^, ^17^, ^18^, ^19^, ^20^, ^21^, ^22^ ^]^ the topic of supramolecular photovoltaics is scarcely explored. Recently, a self‐assembled molecular *p*–*n* junction capable of a long charge‐separated state lifetime (26 ms for the cathode), demonstrates that supramolecular candidates have the potential to replace silicon‐based photovoltaics.^[^ ^10^, ^13^, ^14^, ^23^, ^24^ ^]^

The dye‐sensitized solar cell (DSSC), first reported by Grätzel and O'Regan, is a thin‐film technology for the direct conversion of solar energy to electricity.^[^ ^25^ ^]^ In contrast to a silicon solar cell, light harvesting and charge separation events in a DSSC are decoupled and performed by different components. State‐of‐the‐art devices exceed power conversion efficiencies (PCEs) of 15.2% for *n*‐type DSSCs,^[^ ^26^ ^]^ while the complementary *p*‐type DSSC (*p*‐DSSCs) remain subordinate with a current PCE record of 2.51%.^[^ ^27^, ^28^ ^]^ This disparity in performance precludes the widespread application of tandem DSSCs—especially to photoelectrochemical cells for solar fuel formation—and therefore, these efficiency losses must be addressed.^[^ ^29^, ^30^, ^31^, ^32^, ^33^, ^34^ ^]^

**Figure 1:**
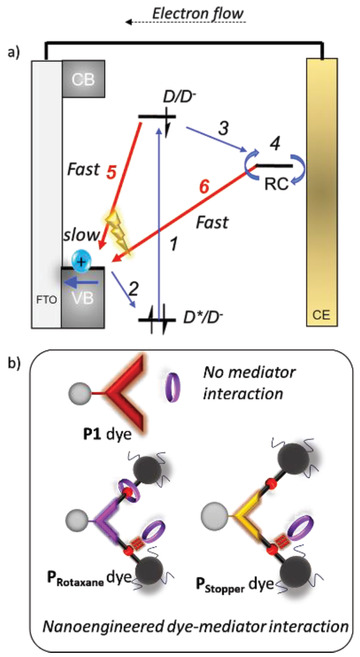
a) Schematic representation of forward electron propagation steps (1–4) (blue arrows) and recombination pathways (5, 6) (red arrows) leading to efficiency losses within *p*‐DSSCs. FTO = fluorine‐doped tin oxide; band; CB = conduction band; D = dye; RC = redox couple. a) Schematic representation of the benchmark system P1 dye (no mediator interaction) and P_Rotaxane_/P_Stopper_ dyes comprising nanoengineered dye‐mediator interactions. P_Rotaxane_ contains a permanently bound mediator as a built‐in regeneration system and P_Stopper_ is designed as a control to study the influence of a permanently bound mediator. The 3‐NDI‐ring is represented as a purple ring in the figure.

**Figure** **1**a illustrates the role of the different components in the forward electron propagation steps (Figure 1a, processes 1–4) (blue arrows) in *p*‐DSSCs. Upon excitation of the dye (Figure 1a, process 1), hole injection takes place into the valence band (VB) of the semiconductor NiO (Figure 1a, process 2).^[^ ^27^ ^]^ The subsequently reduced dye (D^•−^) transfers an electron to the redox mediator (Figure 1a, process 3), which diffuses towards the counter electrode (CE) (Figure 1a, process 4) for regeneration and closing the cycle. However, after hole injection the charge carriers in NiO^+^|D^•−^ can also recombine (Figure 1a, process 5). Additionally, the reduced mediator can react with NiO^+^ sites (Figure 1a, process 6). These charge recombination pathways (Figure 1a, processes 5 and 6, red arrows) lead to efficiency losses within *p*‐DSSCs.^[^ ^27^, ^35^ ^]^ The low PCE in *p*‐DSSC can be attributed to severe electron–hole recombination caused by the extremely small hole diffusion coefficient in NiO (4 × 10^−8^ cm^2^ s^−1^)^[^ ^36^ ^]^ compared to 3–4 orders of magnitude faster (10^−4^ cm^2^ s^−1^)^[^ ^37^ ^]^ electron diffusion in TiO_2_.^[^ ^38^, ^39^, ^40^ ^]^ Therefore, fast and directional transport of charges after separation is crucial to promote forward electron propagation.

To compete with recombination pathways, dye–mediator interactions have been introduced in *p*‐DSSCs to promote fast‐forward electron transfer from the dye to the redox mediator by improving the proximity of this species, thereby enhancing the regeneration rate of the dye.^[^ ^41^, ^42^, ^43^, ^44^, ^45^ ^]^ Previously, we introduced a pseudorotaxane strategy to pre‐organize a tetracationic mediator close to the dye, elevating the photocurrent density (*J*_SC_) tenfold.^[^ ^46^ ^]^ Following this, we developed a new system where a neutral naphthalene diimide macrocycle (3‐NDI‐ring) forms pseudorotaxanes with the dye via the 1,5‐dioxynaphthalene recognition sites (DNP‐arms), and serves as redox mediator. After photoinduced electron transfer (PET), the 3‐NDI‐ring dethreads, effectively removing the charge away from the NiO^+^–D^0^ interface (NiO^+^|D).^[^ ^47^ ^]^ DSSCs based on such molecular mechanical systems show an increase in hole lifetime by a factor of two, enhancing the open circuit voltage (*V*_OC_) and improving the PCE 5‐fold compared to the benchmark system P1 which does not facilitate dye–mediator interactions.

**Figure 2:**
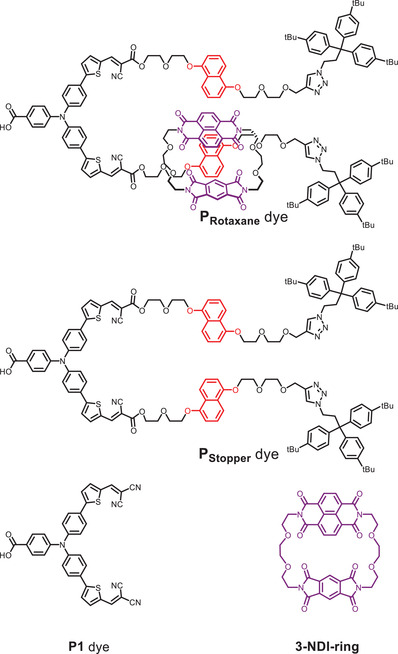
Molecular structures of the benchmark dye P1, the macrocyclic redox mediator 3‐NDI‐ring, P_rotaxane_, and P_Stopper._

In this work, we explore the potential of a new design approach that includes a rotaxane topology (**Figure** **2**) and investigate the DSSC performance. The 3‐NDI‐ring is used as a redox mediator, possibly leading to mediator pre‐organizations via weak interactions with the stoppered treats that have 1,5‐dioxynaphthalene recognition sites not occupied with a permanent ring. We hypothesize that the P_Rotaxane_ system featuring the nanoengineered strategy for dye‐regeneration inspired by former dye–mediator interactions^[^ ^46^ ^]^ will lead to the inhibition of recombination pathways 5 and 6 (Figure 1a) with concomitant promotion of forward electron propagation within the device.

## 2 Results and Discussion

### 2.1 Synthesis and Characterization of the Dyes

**Figure 3:**
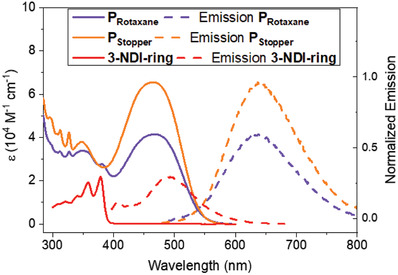
UV–vis spectra in MeCN of P_Rotaxane_ (solid violet line), P_Stopper_ (solid orange line), and compound 3‐NDI‐ring (red solid line) along with the normalized fluorescence spectrum upon excitation at *λ*_max_ (P_Rotaxane_: violet dashed line, P_Stopper_: orange dashed line, 3‐NDI‐ring: red dashed line). Fluorescence spectra are normalized with respect to the fluorescence intensity of P_Stopper_ at 295 nm.

All compounds were prepared according to standard synthetic procedures described in Section S1.2, Supporting Information. Both the P_Rotaxane_ and P_Stopper_ dyes were fully characterized by NMR (^1^H, ^13^C, ^1^H DOSY NMR), Electrospray Ionization High Resolution Mass spectrometry (ESI‐HRMS), and spectrophotometric techniques. The large size of these dyes prompted the determination of the diffusion coefficient by ^1^H DOSY NMR. Subsequent calculation of the molecular radius (*r*, Figure S11, Supporting Information) revealed P_Rotaxane_ is larger (*r* = 1.3 nm) than P_Stopper_ (*r* = 1.1 nm), ascribed to the presence of the 3‐NDI‐ring. The absorption and fluorescence spectra in MeCN solutions of P_Rotaxane_, P_Stopper_, and 3‐NDI‐ring are given in **Figure** **3** and summarized in **Table** **1**. The UV–vis spectrum of reference compound P_Stopper_ features an absorption maximum at 462 nm attributed to intramolecular charge transfer (ICT) from the triphenylamine donor to the cyanoacrylate acceptor.^[^ ^47^ ^]^ Additionally, P_Stopper_ features absorption bands centered at 295, 311, and 326 nm, from the DNP‐arms attached to the cyanoacrylate moiety.

**Table 1:** Optical and electrochemical properties of P_Rotaxane_ and P_Stopper_ (0.2 mM) in DCM (0.1 M TBAPF_6_, glassy carbon working electrode, a leakless Ag/AgCl reference electrode, Pt wire counter electrode.

Dye	*λ*_max_ [nm]	ε × 10^4^ [M^−1^ cm^−1^]	E_0‐0_ [eV]	E_D/D_^+^	E_D/D_‐	Dye coverage × 10^−7^ [mol cm^−2^]
P_Stopper_	462	6.6	2.29	1.10	−0.89	1.58
P_Rotaxane_	467	4.2	2.30	1.17	−0.89	1.07

^a)^ Ferrocene/ferrocenium (Fc/Fc+) was added as an internal redox standard to determine the redox potentials versus NHE (E1/2 Fc/Fc+ = 630 mV vs NHE in MeCN^[^ ^48^ ^]^ and 700 mV vs NHE in DCM).^[^ ^49^ ^]^

^b)^ E_0‐0_ (eV) determined from the intersection between the normalized absorption and fluorescence spectra.

Introduction of the electron acceptor 3‐NDI‐ring into the dye structure to yield P_Rotaxane_ results in a slight red shift of the ICT absorbance to 465 nm compared to the control compound P_Stopper_, likely originating from an additional charge‐transfer band (@ 460 nm) upon interlocking of 3‐NDI‐ring and the DNP‐arms. The emergence of the charge‐transfer band upon combining 3‐NDI‐ring with the dihydroxynaphthalene recognition site was established in previous work,^[^ ^47^ ^]^ where the mixing of 3‐NDI‐ring and P_STATION_ prompts both broadening and red shifting of the UV–vis spectrum as a result of pseudorotaxane formation. In this work, we observe the same broadening/red shift phenomenon upon comparing the spectrum of P_Rotaxane_ with P_Stopper_. This similarity gives strong evidence for CT‐band formation at 460 nm upon rotaxane formation. An additional absorption band in the P_Rotaxane_ spectrum centered at 378 nm is ascribed to the mechanically interlocked 3‐NDI‐ring. These new absorption features in P_Rotaxane_ coincide with a reduction (≈30%) in molar absorptivity (*ε*), ascribed to the mechanically interlocked 3‐NDI‐ring, and consistent with the earlier observation that host–guest complex formation can attenuate the molar absorptivity of the host.^[^ ^50^ ^]^

**Figure 4:**
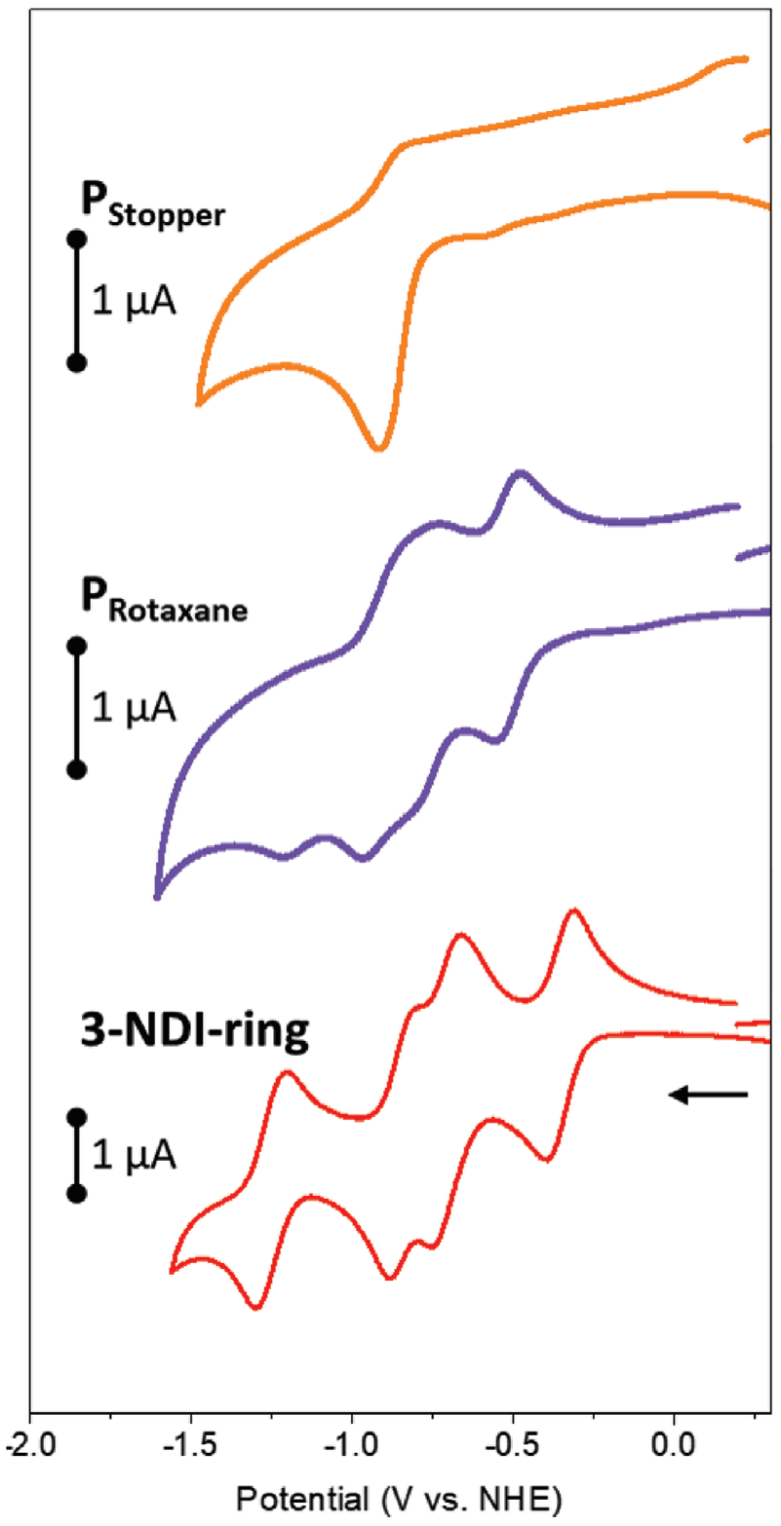
Cyclic voltammograms (0.1 M TBAPF_6_ in DCM, 0.1 V s^−1^) of P_Stopper_ (orange line), P_Rotaxane_ (violet solid line) (0.2 mM each), and 3‐NDI‐ring (red line) (0.5 mM). The arrow indicates the scanning direction. Note: The last reduction of 3‐NDI‐ring of P_Rotaxane_ cannot be discerned through CV but is visible through differential pulse voltammetry (DPV, Figure S23, Supporting Information).

Insight into the redox properties of the P_Rotaxane_, P_stopper_, and 3‐NDI‐ring were obtained by cyclic voltammetry with the results summarized in Table 1. The reduction potential of P_Stopper_ (−0.89 vs NHE, **Figure** **4**a) is typical for P1‐derived dyes (P1 = −0.77 V vs NHE in MeCN).^[^ ^46^ ^]^ The small difference in potential is attributed to the respective acceptor strength (i.e., cyanoacrylate in P_Rotaxane_, and P_Stopper_ vs (dicyano)vinyl in P1). The electrochemistry of 3‐NDI‐ring includes four reduction events (Figure 4b), attributed to two, individual double‐reduction events at the NDI (at −0.35 and −0.71 V vs NHE) and pyromellitic electron‐accepting moieties within 3‐NDI‐ring (at −0.81 and −1.3 V vs NHE). The cyclic voltammogram of P_Rotaxane_ displays multiple reduction events between ‐0.5–1.4 V, ascribed to both the presence of the dye and the 3‐NDI‐ring. The reduction events observed for P_Rotaxane_ are clearly shifted in comparison to the free 3‐NDI‐ring. The reduction events of the 3‐NDI‐ring moiety within P_Rotaxane_ include double‐reduction events at the NDI (at −0.50 and −0.80 V vs NHE) and pyromellitic electron‐accepting moieties within 3‐NDI‐ring (at −0.92 V, fourth reduction not visible with a CV but is clearly demonstrated by DPV to appear at −1.4 V vs NHE, Figure S23, Supporting Information).

### 2.2 Ultrafast Spectroscopy Studies

Photoinduced charge propagation in P_Rotaxane_ that occurs upon installing a permanently‐bound redox mediator was further investigated by time‐resolved fluorescence and femtosecond transient absorption (fs‐TA) spectroscopy measurements using FTO glass substrates with layers of the P_Rotaxane_ and P_Stopper_ dyes on ZrO_2_ and NiO immersed in a supporting electrolyte (1.5 mL, 1 M LiTFSI valeronitrile/MeCN, v/v, 15:85).

**Figure 5:**
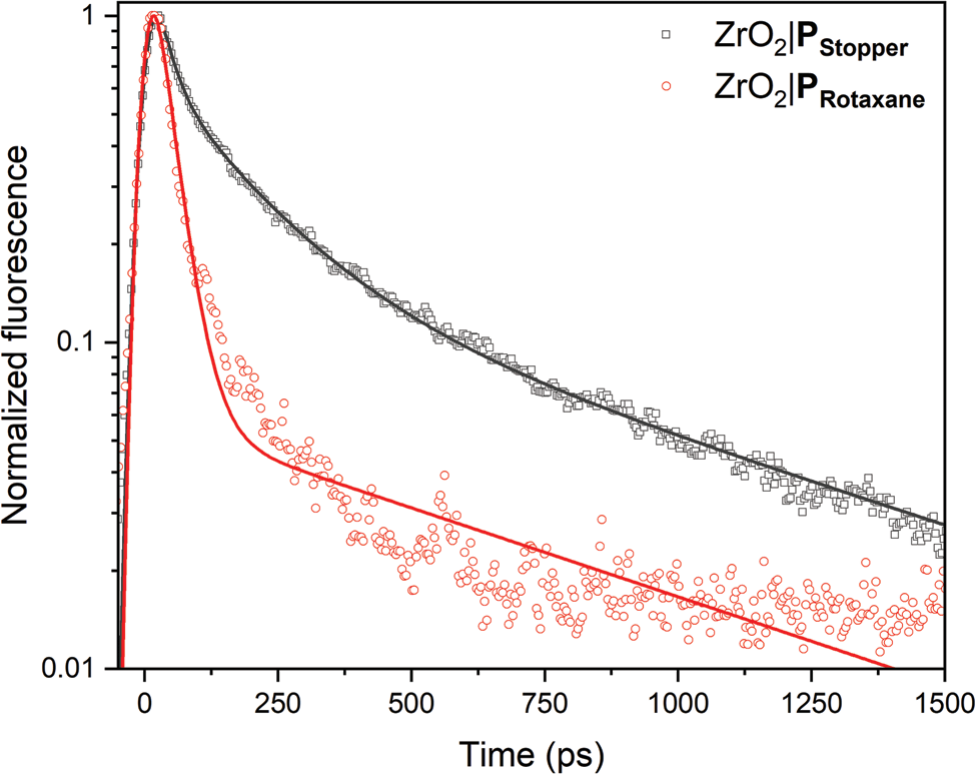
Decay of the normalized fluorescence signal at 615 nm (*λ*_exc._ = 532 nm) and fits for both ZrO_2_|P_Stopper_ and ZrO_2_|P_Rotaxane_. Note that the fluorescence of the latter is quenched, negatively affecting the signal‐to‐noise ratio and quality of the fit.

To study the occurrence of intramolecular electron transfer from P_Rotaxane_ to the interlocked 3‐NDI‐ring on solid substrates, we conducted time‐resolved fluorescence experiments (*λ*_exc._ = 532 nm) on ZrO_2_ **|**P_Rotaxane_ and ZrO_2_ **|**P_Stopper_. **Figure** **5** demonstrates the decay of the normalized fluorescence signal at 615 nm for both ZrO_2_|P_Stopper_ and ZrO_2_|P_Rotaxane_. Compared to ZrO_2_|P_stopper_, the fluorescence of ZrO_2_|P_rotaxane_ is quenched, demonstrating that fast electron transfer from the dye part of P_Rotaxane_ to the 3‐NDI‐ring indeed takes place. The associated time constant (listed in Figure S27 and Table S2, Supporting Information) for *τ*_1_  is ∼30 ps, while the small fluorescence signal that remains (Figure S27d, Supporting Information) is most likely due to the emission of P_Rotaxane_ that has not undergone electron transfer (*τ*_2_ ∼800 ps). In the case of ZrO_2_|P_Stopper_ **_,_** the slower fluorescence decay indicates that the fluorescence is not quenched and no fast photoinduced electron transfer takes place (*τ*_1_ = 25.2 ± 0.1 ps; *τ*_2_ = 166.6 ± 0.2 ps; *τ*_3_ = 823.6 ± 0.8 ps).

To further understand the electron propagation upon photoexcitation of P_Rotaxane_ and P_Stopper_ dyes on the metal oxide substrates, we also conducted fs‐TA experiments. Previously, we established that the transient behavior of P1 as a benchmark system under analogous conditions is consistent with reports from the literature.^[^ ^47^, ^51^, ^52^, ^53^ ^]^ In the case of NiO|P1, four distinct time constants are apparent from photophysical modeling, indicating that biphasic photoinduced hole injection (ultrafast, i.e., within a few hundred fs, and in 1–2 ps) is followed by a fast (≈5–10 ps) and a slow (>100 ps) charge recombination step.^[^ ^51^, ^52^, ^53^ ^]^ The fs‐TA data of P_Rotaxane_ and P_Stopper_ in solution (Figure S25, Supporting Information) and on ZrO_2_ (Figure S26, Supporting Information) are discussed in Section S2.2 of the Supporting Information.

**Figure 6:**
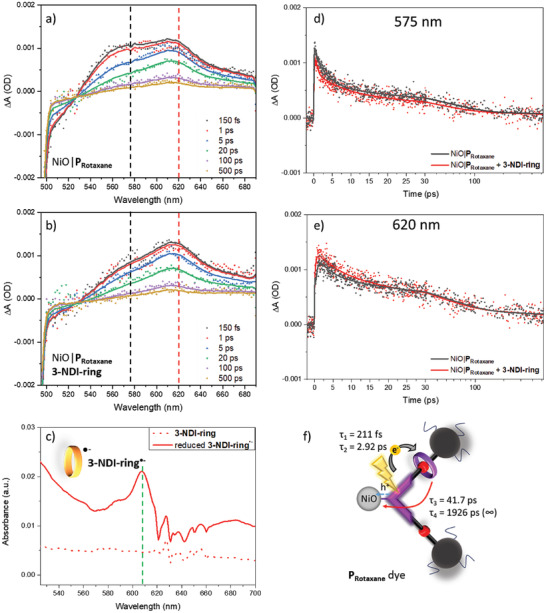
Transient absorption (TA, *λ*_exc._ = 480 nm) data and fits (see Section S2, Supporting Information for details) of P_Rotaxane_ on NiO in supporting electrolyte (1.5 mL, 1 M LiTFSI valeronitrile/MeCN, 15:85) in absence and presence of the 3‐NDI‐ring (5.9 mM). a) TA spectra at given time delays in the absence of the 3‐NDI‐ring. The photoinduced absorbance of NiO|P_Rotaxane_ around 575 nm is indicated with a black dashed line and indicative of a combination of P_Rotaxane_ ^*^ and P_Rotaxane_^•^ **^−^**; b) TA spectra at given time delays in the presence of the 3‐NDI‐ring**_;_** c) Spectroelectrochemistry of the 3‐NDI‐ring (dotted green line), which shows an absorption band around 608 nm when the 3‐NDI‐ring^•^ **^‐^** is formed (solid red line); d) Kinetic traces at 575 nm with (red) and without 3‐NDI‐ring present in the electrolyte (black); e) Kinetic traces at 620 nm with (red) and without 3‐NDI‐ring present in the electrolyte (black); f) Schematic representation of charge transfer processes following excitation of the P_Rotaxane_ dye in absence of free 3‐NDI‐ring.

The fs‐TA spectra of NiO|P_Rotaxane_ at various time delays after 480 nm excitation are presented in **Figure** **6**a, and further detailed in Section S2 of the Supporting Information. Analogous to the NiO|P1 benchmark system,^[^ ^51^, ^52^, ^53^ ^]^ the negative signal < 560 nm is due to ground state bleach, while the photoinduced absorbance around 575 nm (black dashed line) can be assigned to P_Rotaxane_^*^ and P_Rotaxane_^•−^_._ The latter likely has a slightly red‐shifted absorption band relative to P_Rotaxane_ ^*^ and is formed due to photoinduced hole injection into NiO, either within a few hundred fs or in 1–2 ps.^[^ ^51^, ^52^, ^53^ ^]^ This assignment is in line with the spectroelectrochemical measurements of P_Rotaxane_ that show a 22 nm red‐ shift of P_Rotaxane_^•^ **^−^** compared to P_Rotaxane_ (Figure S24, Supporting Information).

The fs‐TA spectra of NiO|P_Rotaxane_ in the presence of the 3‐NDI‐ring shown in Figure 6b diverge significantly from the data presented in Figure 6a, with a more intense photoinduced absorbance around 620 nm. Based on the fluorescence quenching demonstrating photoinduced reduction of the ring (Figure 5) and the spectroelectrochemistry data (3‐NDI‐ring^•^ **^−^** λ_max_ ≈608 nm, Figure 6c, green dashed line), we assign this additional TA signal to the 3‐NDI‐ring^•^ **^−^**. The observed ∼12 nm red‐ shift of the interlocked 3‐NDI‐ring^•^ **^−^** moiety in NiO|P_Rotaxane_^•^ **^−^** (with respect to free diffusing 3‐NDI‐ring^•^ **^−^**) is likely due to photoinduced charge separation with the NiO, and could also be a consequence of the interlocked 3‐NDI‐ring^•^ **^−^** (vs free 3‐NDI‐ring^•^ **^−^**).^[^ ^54^, ^55^, ^56^ ^]^

The TA signal at 620 nm fully develops within 150 fs after photoexcitation, indicating that ultrafast hole injection from the P_Rotaxane_ dye into the valence band of NiO coincides with the formation of the 3‐NDI‐ring^•^ **^−^**. Intramolecular charge transfer towards the ring is hence likely also an ultrafast process, photophysical modeling gives a time constant of 210 ± 7 fs. The intensity of the photoinduced absorbance at ≈575 nm decreases over time relative to the photoinduced absorption at ≈620 nm, which can be ascribed to slow (2.92 ± 0.05 ps) hole injection from P_Rotaxane_ into the NiO coupled to electron transfer to the 3‐NDI‐ring. The ground state bleach beyond these times indicates that not all excited complexes give both hole injection and electron transfer with these time constants, which may be a result of structural inhomogeneity of the P_Rotaxane_ dye, as further explained in Section S2.2, Supporting Information. Subsequent decay of the 575 and 620 nm photoinduced absorption bands, further illustrated by the kinetic traces in Figure 5d,e, indicates charge recombination. Details regarding the photophysical modeling, resulting in the fits included in Figure 6, are given in Section S2, Supporting Information. In this model, photoinduced hole injection partly occurs simultaneously with 3‐NDI‐ring reduction, while the other dye radical anions give slower ring reduction (time constants in Section S2.2, Supporting Information), possibly due to structural inhomogeneity. The obtained charge transfer times are illustrated in Figure 6f.

In the 3‐NDI‐ring containing electrolyte (Figure 6b), the fs‐TA spectra are nearly identical to those in the electrolyte without added 3‐NDI‐ring (Figure 6a), featuring two photoinduced absorbance bands centered around 575 and 620 nm. The comparison of kinetic traces indicates that in the 3‐NDI‐ring containing electrolyte the transient signals at 575 nm (Figure 6d) and 620 nm (Figure 6e) decay a little bit faster. Photophysical modeling seems to suggest slightly faster ring reduction and charge recombination (Table S4, Supporting Information, *τ*_2,_ *τ*_3_, *τ*_4_). Though the differences are small, they may originate from the 3‐NDI‐ring in the electrolyte acting as an electron acceptor, that is, the respective intra‐ and inter‐molecular electron transfer processes cannot be decoupled and direct transfer to the unbound redox mediator cannot be excluded.

### 2.3 Proposed Electron Propagation within NiO|P_Rotaxane_

**Figure 7:**
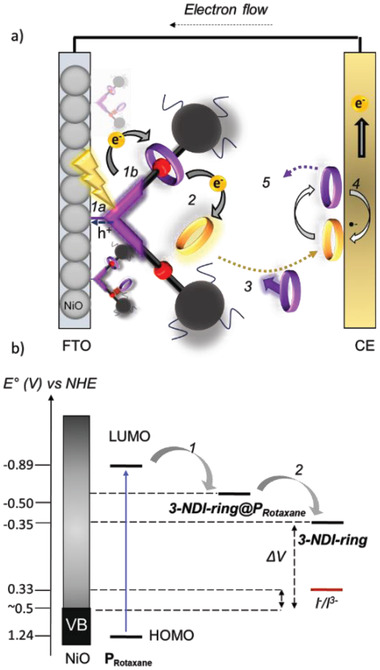
a) Schematic representation of charge propagation of the rotaxane‐based *p*‐DSSC. b) Schematic energy diagram for the *p*‐DSSC based on the P_Rotaxane_/P_Stopper_ dyes. Upon excitation of the P_Rotaxane_ hole injection takes place (process 1a) and simultaneously the mechanically bound 3‐NDI‐ring@P_Rotaxane_ is reduced (process 1b). Then, the reduced 3‐NDI‐ring^•−^@P_Rotaxane_ is able to reduce the 3‐NDI‐ring present in the bulk solution (process 2). Energy levels are represented in V versus NHE.

With the photophysical properties and electrochemical data in hand, we can construct an energy diagram and predict how the electrons propagate within the P_Rotaxane_ system (**Figure** **7**). The electrochemical studies reveal that the thermodynamics of the rotaxane‐dye support electron transfer from the reduced dye P_Rotaxane_^•^ **^−^** to the interlocked 3‐NDI‐ring. As the redox potential of the “free” 3‐NDI‐ring is less negative than of the interlocked 3‐NDI‐ring, electron transfer is exergonic from the anionic interlocked 3‐NDI‐ring^•^ **^−^** and freely diffusing 3‐NDI‐ring in the bulk electrolyte. Furthermore, fs‐TA results demonstrate the fast kinetics of electron transfer from the reduced dye P_Rotaxane_^•^ **^−^** to the interlocked 3‐NDI‐ring and the effect of a second “free” 3‐NDI‐ring possibly acting as an electron acceptor.

Figure 7a illustrates the proposed rotaxane‐based *p*‐DSSC consisting of the P_Rotaxane_ dye. The molecular structure of P_Rotaxane_ (Figure 2) contains one DNP‐arm that is mechanically bound to the 3‐NDI‐ring as the tris(4‐*t*‐butylphenyl)methyl stoppers prevent slippage of the macrocyclic 3‐NDI‐ring. The qualitative binding studies described in Section S1.5, Supporting Information estimated from UV–vis and ^1^H NMR binding data, suggest that the other DNP‐arm could be available for an interaction with a second 3‐NDI‐ring by an alternate binding mode (vide supra). The interaction of the second 3‐NDI‐ring might be based on π‐stacking between the DNP‐arm and the naphthalene diimide. According to our fs‐TA studies, upon photoexcitation of the dye, the electron is transferred very quickly to the proximal 3‐NDI‐ring within the P_Rotaxane_ structure (Figure 7a, step 1). This process likely occurs simultaneously with the hole transfer to the NiO (process 1a). The interlocked 3‐NDI‐ring^•^ **^−^** anion is able to reduce a 3‐NDI‐ring from solution, that may be preorganized via weak interactions with the adjacent DNP‐arm (Figure 7a, step 2). This free 3‐NDI‐ring^•^ **^−^** (Figure 7a, step 3) is subsequently regenerated at the counter electrode (Figure 7a, step 4). The P_Stopper_ reference dye features both DNP‐arms for binding with 3‐NDI‐ring via π–interactions but no rotaxane‐bound 3‐NDI‐ring allowing it to function as a reference to investigate the role of the mechanically‐bound mediator in P_Rotaxane_. Having established that both the thermodynamics and kinetics are in favor of forward electron propagation, the influence of the rotaxane design can be investigated in the *p*‐DSSC.

### 2.4 Photovoltaic Performance

Next, we explored the effect of the rotaxane topology on the performance of the *p*‐DSSC. The *p*‐DSSCs were prepared using screen‐printed NiO photocathodes (3.5 µm, 0.2 cm^−2^) that were sensitized with P_Stopper_ or P_Rotaxane_ in MeCN solution (0.15 mM) for 16 h. Dye uptake experiments (Section S1.6, Supporting Information.) revealed that the surface coverage of P_Stopper_ (*Γ* = 1.58 × 10^−7^ mol cm^−2^) is ≈50% higher than that of P_Rotaxane_ (*Γ* = P_Rotaxane_ 1.07 × 10^−7^ mol cm^−2^), which is expected based on the increase molecular size incurred by P_Rotaxane_ upon rotaxane formation with 3‐NDI‐ring (*r* = 1.1 nm for P_Stopper_ vs *r* = 1.3 nm for P_Rotaxane_, Figure S11, Supporting Information).

Initial assessment of *p*‐DSSC devices was performed using the I^−^/I_3_^−^ redox electrolyte (1 M LiI and 0.1 M I_2_ in MeCN). We also fabricated *p*‐DSSCs with the dye P1 as a benchmark system *p*‐DSSC affording a PCE (0.061% ± 0.002%), comparable to the best performing analogous devices literature (0.075%) (see Section 3.6, Supporting Information).^[^ ^57^ ^]^ The small discrepancies between our P1 cells and literature values are ascribed to the quality of the NiO, which is well known to vary based on several factors, such as nanoparticle size and Ni^3+^ impurities.^[^ ^57^, ^58^ ^]^

**Figure 8:**
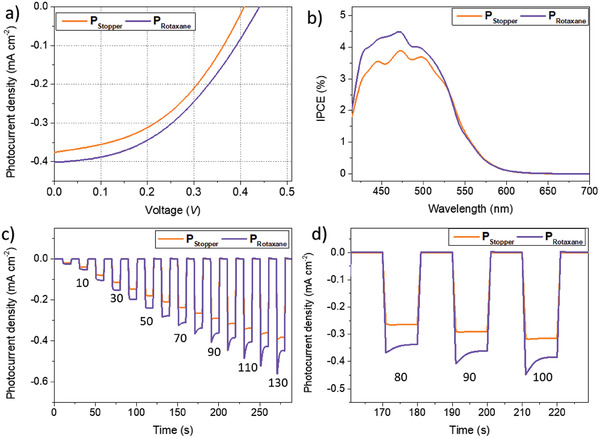
Photovoltaic performances of the devices based on the P_stopper_ (orange line) and the P_Rotaxane_ dye (violet line) with the 3‐NDI‐ring as redox mediator (25 mM 3‐NDI‐ring/3‐NDI‐ring^•^ **^−^** 1:1 in 1 M LiTFSI valeronitrile/MeCN, 15:85). a) *J*–*V* curves of the on the P_stopper_ (orange line) and the P_Rotaxane_ dye (violet line). b) Photocurrent action spectrum. c) Chopped light amperometry at different light flux varying from 0.05–1.3 W cm^−2^ with on/off cycles of 10 s. d) Zoom of chopped light amperometry at 80, 90, and 100 mW cm^−2^ clearly showing tailing behavior indicative of mass transfer limitation.

The *p*‐DSSCs were prepared employing the 3‐NDI‐ring electrolyte (25 mM 3‐NDI‐ring/3‐NDI‐ring^•^ **^−^,** 1:1 in 1 M LiTFSI valeronitrile/MeCN, 15:85) with a 60 µm thermoplast frame. Poly(3,4‐ethylenedioxythiophene) (PEDOT) counter electrode was employed instead of the typically used Pt, to take advantage of its lower charge transfer resistance against organic radical redox couples.^[^ ^59^ ^]^ The photocurrent–voltage (*J*–*V*) characteristics (**Figure** **8**a) of the *p*‐DSSCs based on P_Rotaxane_ and P_Stopper_ with the 3‐NDI‐ring electrolyte were measured under AM 1.5G illumination (100 mW cm^−2^), with the data summarized in **Table** **2**. The *p*‐DSSCs sensitized with P_Rotaxane_ demonstrate an increase in performance compared to the analogue based on the P_Stopper_ system demonstrating that the permanently present redox mediator as a dye design feature has an overall positive effect on the *p*‐DSSC performance. Not only does P_Rotaxane_ demonstrate a higher *V*_OC_ than P_Stopper_ (0.43 vs 0.36 V, respectively), accompanying improvements to *J*_SC_ (0.39vs 0.34 mA cm^−2^, respectively) result in a better PCE (0.07% vs 0.05% respectively). Furthermore, the incident photon‐to‐current efficiency (IPCE) of P_Rotaxane_‐based *p*‐DSSC (IPCE_max_ = 4.49% at 471 nm) is greater across the whole spectrum compared to P_Stopper_ analogue (IPCE_max_ = 3.88% at 474, Figure 8b). The difference between the *p*‐DSSCs was further characterized by their photocurrent response upon varying light intensity, through chopped‐light amperometry (Figure 8c,d). The *p*‐DSSCs based on the P_Rotaxane_ demonstrate a higher *J*_SC_ upon illumination at every light intensity from 414–700 compared to P_Stopper_, which is which is in line with the *J*–*V* characterization (Figure 8a). The shape of the photocurrent response exhibits tailing where the signal decays, especially at higher *J*_SC_, indicative of mass transfer limitations in the device from slow diffusion of the redox mediator hampering current generation.^[^ ^60^, ^61^ ^]^

**Table 2:** Summary of the photovoltaic performance data for *p*‐DSSC based on P_Stopper_ and P_Rotaxane_ under AM 1.5G illumination (0.1 W cm^−2^) employing the 3‐NDI‐ring electrolyte (25 mM) in 1 M LiTFSI valeronitrile/MeCN, (15:85). The average performance (*N* = 9) is provided with the best‐performing cell in brackets.

Dye	*V*_OC_ [V]	*J*_SC_ [mA cm^−2^]	FF	PCE [%]
P_Stopper_	0.36 ± 0.05 (0.41)	−0.34 ± 0.08 (−0.42)	0.41 ± 0.04 (0.45)	0.05 ± 0.02 (0.07)
P_Rotaxane_	0.43 ± 0.04 (0.47)	−0.39 ± 0.03 (−0.40)	0.40 ± 0.03 (0.43)	0.07 ± 0.01 (0.08)

As the association of the 3‐NDI‐ring‐mediator to P_Rotaxane_ is weak (i.e., < 10 M^−1^), diffusional processes dominate the behavior of the electrolyte, leading to a decrease in the amount of dye–mediator pre‐organization. This tailing behavior was also observed in a former study where P1 was combined with 3‐NDI‐ring, having no supramolecular interaction. In case a supramolecular interaction is established between the dye and 3‐NDI‐ring by means of pseudorotaxane formation, the tailing behavior disappears by pre‐organization of the mediator to the dye.^[^ ^47^ ^]^

### 2.5 Electrochemical Impedance Spectroscopy

To further understand the suppression of recombination in devices based on P_Rotaxane_ electrochemical impedance spectroscopic (EIS) measurements were carried out at different light intensities. The results were analyzed using the transmission line model with the addition of a Warburg element to simulate electrolyte diffusion (See Section S3.7, Supporting Information).^[^ ^62^ ^]^ The chemical capacitance C_µ_ reveals a slight upwards (28 mV) shift of the valance band for P_Rotaxane_ (Figure S35, Supporting Information), which can be assigned to the difference in dye loading, leaving less NiO sites exposed to interact with ions present in the electrolyte.^[^ ^62^, ^63^ ^]^ The recombination resistance (*R*_REC_) is double for P_Rotaxane_ devices (40.8 × 10^3^ Ω cm^−2^ at 100 mV) compared to those based on P_Stopper_ (21.8 × 10^3^ Ω cm^−2^ at 100 mV) implying less recombination occurs at the NiO–dye interface in the P_Rotaxane_ *p*‐DSSCs. This difference in R_REC_ (at any given voltage) translates into a 50% increase in hole lifetime for P_Rotaxane_ *p*‐DSSCs (811 ms at 100 mV) compared to devices based on P_Stopper_ (527 ms at 100 mV). The decrease in recombination in the P_Rotaxane_ system compared to P_Stopper_, translates into an extended hole lifetime, leading to a higher *V*_OC_, *J*_SC_, and PCE.

To demonstrate the effect of the rotaxane‐based dye design on the charge recombination, the hole lifetimes (at 100 mV) of the devices based on the different P1‐derived dyes P_Rotaxane_ and P_Stopper_ are summarized together with PCE and dye loading in **Table** **3**. The *p*‐DSSC devices using I^−^/I_3_^−^ electrolytes were also assessed to serve as a point of reference, being a system exhibiting no diffusional limitation.

**Table 3:** Overview of hole lifetimes (*τ*_h_) of different systems at 100 mV. The higher hole lifetime of the systems in this study originates from the suppression of charge recombination at the NiO|Dye interface.

Dye	Dye loading [Γ, × 10^−7^ mol cm^−2^]	Electrolyte	PCE [%]	τ_h_ [ms, at 100 mV]
P_Stopper_ ^a)^	1.07	25 mM 3‐NDI‐ring	0.05	527
		1 M I^−^/I_3_^−^	0.03	28.5
P_Rotaxane_ ^a)^	1.58	25 mM 3‐NDI‐ring	0.07	811
		1 M I^−^/I_3_^−^	0.04	49.7
P1^b)^	3.24	25 mM 3‐NDI‐ring	0.01	324
		1 M I^−^/I_3_^−^	0.06	113
		25 mM I^−^/I_3_^−^	0.01	N/A

^a)^ This work

^b)^ Former work.^[^ ^47^ ^]^

The benchmark *p*‐DSSC employing P1 and I^−^/I_3_^−^ electrolyte affords a τ_h_ of 113 ms, which is close to that reported in literature (356 ms in literature^[^ ^63^ ^]^), the difference ascribed to the quality of the NiO, which is known to vary based on several factors, such as nanoparticle size^[^ ^57^ ^]^). In comparison, the P_Rotaxane_ and P_Stopper_ dyes combined with I^−^/I_3_^−^ electrolytes demonstrate shorter hole lifetimes (50 and 29 ms respectively). These low τ_h_ values indicate severe recombination at the Dye–NiO interface, in line with the lower dye loading in these cells, leaving many NiO^+^‐sites exposed (*Γ* = 1.58 × 10^−7^ and 1.07 × 10^−7^ mol cm^−2^ for P_Rotaxane_ and P_Stopper_ respectively, compared to *Γ* = 3.24 × 10^−7^ mol cm^−2^ for P1). While these hole lifetimes are much lower compared to the electron lifetimes observed for typical *n*‐DSSCs (≈3500 ms^[^ ^63^ ^]^), the discrepancy in charge carrier lifetime is expected given the severe charge recombination in these *p*‐type DSSCs using I^−^/I_3_^−^ as an electrolyte.

Due to the limited solubility of the 3‐NDI‐ring, the *p*‐DSSCs based on this mediator were prepared with a 40 times lower redox mediator concentration compared to those based on I^−^/I_3_^−^ as electrolyte. The *p*‐DSSCs employing the 3‐NDI‐ring (25 mM) as electrolyte demonstrate much longer hole lifetimes compared to cells with the 1 M I^−^/I_3_^−^ as electrolyte. We observe that a short hole lifetime results in a lower PCE, consistent with the data of P1 devices using 25 mM I^−^/I_3_^−^ electrolyte.

A trend in the hole lifetime **P1 < P_Stopper_ < P_Rotaxane_** was observed in all devices, attributed to the supramolecular localization of the redox mediator in close proximity to the dye through a rotaxane‐topology, increasing the sensitizer regeneration rate during device operation. The hole lifetimes increase by a factor of 16–18 compared to the I^−^/I_3_^−^ cells, despite the lower electrolyte (25 mM for 3‐NDI‐ring vs 1 M for I^−^/I_3_^−^) concentration. A direct comparison between the 1 M I^−^/I_3_^−^ electrolyte and 3‐NDI‐ring (25 mM) is inadequate, because of the difference in electrolyte concentration and molecular size. However, we do observe a general trend when moving from 1 M I^−^/I_3_^−^ electrolyte to 3‐NDI‐ring (25 mM) in higher *τ*_h_, leading to improved *V*_OC_ and enhanced PCE (Table 3).

The diffusion (*D*_e_) of redox mediators in devices employing macrocycles (25 mM 3‐NDI‐ring, *D*_e_ = 1.4 **×** 10^−10^ m^2^ s^−1^) is approximately four times slower than for I^−^/I_3_^−^ (1 M, *D*_e_ = 4.85 **×** 10^−10^ m^2^ s^−1^) (see Section S3.11, Supporting Information). This difference in diffusion is in line with the mass‐transfer limitations observed in the chopped light experiments (Figure 8c). Furthermore, as the *r* of iodide (*r* = 0.2 nm)^[^ ^64^ ^]^ is 3.5 times smaller than 3‐NDI‐ring (*r* = 0.7 nm, Figure S11, Supporting Information) the former is expected to experience faster diffusion kinetics.

Despite the slow diffusion kinetics, the 3‐NDI‐ring‐based *p*‐DSSCs using the P_Rotaxane_ system show astonishing τ_h_ values of ≈1000 ms, which compares to electron lifetimes found in *n*‐DSSC. This trend in *τ*_h_ is also reflected in the differences in PCEs, with the P_Rotaxane_ 3‐NDI‐ring (25 mM) system showing an increased PCE compared to P1 p‐DSSCs using 1 M I^−^/I_3_^−^ (0.07% vs 0.06%). Hence, despite the 40× lower concentration of 3‐NDI‐ring the current device has an increased performance compared to that based on the benchmark I^−^/I_3_^−^ electrolyte. The increased PCE for the P_rotaxane_ 3‐ NDI‐ring p‐DSSC implies that recombination‐dominating losses in P1‐based *p*‐DSSC are addressable by invoking a supramolecular electronic approach. The results of the increased performance of P_Rotaxane_‐based *p*‐DSSC are consistent with the fs‐TA results that suggest that an additional 3‐NDI‐ring in the electrolyte possibly acts as an electron acceptor. Although the difference in time constants relative to the system in electrolytes without an additional 3‐NDI‐ring is small, the effects can be significant. Beneficial effects possibly arise from the 3‐NDI‐ring in the electrolyte promoting hole injection by P_Rotaxane_ ^*^ into the NiO or accepting an electron from P_Rotaxane_^•^ **^−^**. In all cases, the implementation of dye‐mediator interactions leads to extended hole lifetimes.

### 2.6 Inhibiting Recombination

To demonstrate that we inhibited the recombination, we are determining the theoretical maximum current that we can expect in our device (*J*_lim_) based on the redox mediator of choice and its concentration. From the concentration and diffusion coefficient of the redox mediator, we can determine the mass transfer. The *J*_lim_ is independent of the dye system and is solely dictated by the properties of the electrolyte, (thus *J*_lim_ is not photocurrent, just current). The calculated *J*_lim_ is compared with the photocurrents at short circuit that we observe (*J*_SC_) in our supramolecular dye‐system (3‐NDI‐ring/3‐NDI‐ring^•^ **^−^**) and traditional iodide‐systems (I^−^/I_3_^−^). (see Section S3.10, Supporting Information) based on the I^−^/I_3_^−^ and the 3‐NDI‐ring electrolyte.

By approximation, the solar cells based on the 3‐NDI‐ring electrolyte (12.5:12.5 mM, 3‐NDI‐ring/3‐NDI‐ring^•^ **^−^**) have maximum photocurrent densities of *J_lim(3‐NDI‐ring)_* = 0.6 mA cm^−2^ while that of the I^−^/I_3_^−^ cells are around *J_lim(I3‐)_* = 95 mA cm^−2^.^[^ ^65^ ^]^ This means that under these conditions the *p*‐DSSCs employing the 3‐NDI‐ring reach as much as 60–70% of the theoretical current for the devices based on P_Stopper_ and P_Rotaxane_ respectively, while for cells based on the I^−^/I_3_^−^ (0.9:0.1 M) this is only 1.6%. The fact that the obtained photocurrents approach the limited current, implies that recombination has been suppressed to a minimum and the system based on supramolecular interactions is mostly limited by slow diffusion of the large macrocyclic mediator. This improvement in maximum photocurrent densities is not just an improvement as a result of surface shielding by using a bigger electrolyte as P1 combined with 3‐NDI‐ring approaches 20% of the theoretically achievable current. Despite P_Rotaxane_ dye loading being 50% less than that of P1, the rotaxane system manages improvements in approaching the *J_lim_*. Both the measured τ_h_ and the calculated maximum photocurrent demonstrate that recombination is suppressed to a minimum, implying that the system is mostly limited by low solubility and slow diffusion of the large macrocyclic mediator.

## 3 Conclusion

In conclusion, we report a nanoengineered dye‐regeneration system P_Rotaxane_, where a macrocyclic electron acceptor is permanently bound via rotaxane formation in close proximity to the dye. Femtosecond transient absorption spectroscopy revealed ultrafast (211 ± 7 fs and 2.92 ± 0.05 ps) charge transfer from the P_Rotaxane_ dye to the permanently bound mediator (3‐NDI‐ring). Utilization of rotaxane topologies leads to *p*‐DSSCs with unprecedented hole lifetimes, suppressing charge recombination to a large extent, and providing photocurrents up to 70% of the theoretical maximum. We are currently pursuing the integration of this strategy with broader absorbing, high‐efficiency dyes, which should lead to a new generation of supramolecular *p–n* junctions that engender charge rectification in photovoltaics.

## Conflict of Interest

The authors declare no conflict of interest.

## Supporting information

Supporting Information

Supporting Information

Supporting Information

## Data Availability

The data that support the findings of this study are available from the corresponding author upon reasonable request.

## References

[1] H. H. Mcgarraugh , W. Liu , B. P. Matthews , B. D. Smith , Eur. J. Org. Chem. 2019, 2019, 3489. 10.1002/ejoc.201900082 PMC6774672 31579392

[2] T. H. Ngo , J. Labuta , G. N. Lim , W. A. Webre , F. D'souza , P. A. Karr , J. E. M. Lewis , J. P. Hill , K. Ariga , S. M. Goldup , Chem. Sci. 2017, 8, 6679. 30155230 10.1039/c7sc03165c PMC6103255

[3] E. Arunkumar , N. Fu , B. D. Smith , Chemistry 2006, 12, 4684. 16575935 10.1002/chem.200501541

[4] Y. Goto , T. Hisatomi , Q. Wang , T. Higashi , K. Ishikiriyama , T. Maeda , Y. Sakata , S. Okunaka , H. Tokudome , M. Katayama , S. Akiyama , H. Nishiyama , Y. Inoue , T. Takewaki , T. Setoyama , T. Minegishi , T. Takata , T. Yamada , K. Domen , Joule 2018, 2, 509.

[5] H. Li , A. C. Fahrenbach , A. Coskun , Z. Zhu , G. Barin , Y.‐L. Zhao , Y. Y. Botros , J.‐P. Sauvage , J. F. Stoddart , Angew. Chem., Int. Ed. 2011, 50, 6782. 10.1002/anie.201102510 21717550

[6] C. W. Fuller , P. S. Padayatti , H. Abderrahim , L. Adamiak , N. Alagar , N. Ananthapadmanabhan , J. Baek , S. Chinni , C. Choi , K. J. Delaney , R. Dubielzig , J. Frkanec , C. Garcia , C. Gardner , D. Gebhardt , T. Geiser , Z. Gutierrez , D. A. Hall , A. P. Hodges , G. Hou , S. Jain , T. Jones , R. Lobaton , Z. Majzik , A. Marte , P. Mohan , P. Mola , P. Mudondo , et al., Proc. Natl. Acad. Sci. USA 2022, 119, 2112812119. 10.1073/pnas.2112812119 PMC8812571 35074874

[7] V. K. Sangwan , R. P. Ortiz , J. M. P. Alaboson , J. D. Emery , M. J. Bedzyk , L. J. Lauhon , T. J. Marks , M. C. Hersam , ACS Nano 2012, 6, 7480. 22783918 10.1021/nn302768h

[8] C. Zheng , Y. Liao , S.‐T. Han , Y. Zhou , Adv. Electron. Mater. 2020, 6, 2000641.

[9] L. Wang , L. Wang , L. Zhang , D. Xiang , in Molecular‐Scale Electronics: Current Status and Perspectives (Ed: X. Guo ), Springer, Cham, Switzerland 2019, pp. 45–86.

[10] K. Wang , E. Meyhofer , P. Reddy , Adv. Funct. Mater. 2020, 30, 1904534.

[11] D. Xiang , X. Wang , C. Jia , T. Lee , X. Guo , Chem. Rev. 2016, 116, 4318. 26979510 10.1021/acs.chemrev.5b00680

[12] S. V. Aradhya , L. Venkataraman , Nat. Nanotechnol. 2013, 8, 399. 23736215 10.1038/nnano.2013.91

[13] N. Xin , J. Guan , C. Zhou , X. Chen , C. Gu , Y. Li , M. A. Ratner , A. Nitzan , J. F. Stoddart , X. Guo , Nat. Rev. Phys. 2019, 1, 211.

[14] Y. Liu , X. Qiu , S. Soni , R. C. Chiechi , Chem. Phys. Rev. 2021, 2, 021303.

[15] H. Chen , J. Fraser Stoddart , Nat. Rev. Mater. 2021, 6, 804.

[16] D. C. Milan , M. Krempe , A. K. Ismael , L. D. Movsisyan , M. Franz , I. Grace , R. J. Brooke , W. Schwarzacher , S. J. Higgins , H. L. Anderson , C. J. Lambert , R. R. Tykwinski , R. J. Nichols , Nanoscale 2017, 9, 355. 27924336 10.1039/c6nr06355a

[17] P. Lussis , T. Svaldo‐Lanero , A. Bertocco , C.‐A. Fustin , D. A. Leigh , A.‐S. Duwez , Nat. Nanotechnol. 2011, 6, 553. 21857685 10.1038/nnano.2011.132

[18] H. Wen , W. Li , J. Chen , G. He , L. Li , M. A. Olson , A. C.‐H. Sue , J. F. Stoddart , X. Guo , Sci. Adv. 2016, 2, 1601113. 10.1126/sciadv.1601113 PMC5262467 28138528

[19] Y. Luo , C. P. Collier , J. O. Jeppesen , K. A. Nielsen , E. Deionno , G. Ho , J. Perkins , H.‐R. Tseng , T. Yamamoto , J. F. Stoddart , J. R. Heath , ChemPhysChem 2002, 3, 519. 12465491 10.1002/1439-7641(20020617)3:6<519::AID-CPHC519>3.0.CO;2-2

[20] J.‐H. Tang , Y. Li , Q. Wu , Z. Wang , S. Hou , K. Tang , Y. Sun , H. Wang , H. Wang , C. Lu , X. Wang , X. Li , D. Wang , J. Yao , C. J. Lambert , N. Tao , Y.‐W. Zhong , P. J. Stang , Nat. Commun. 2019, 10, 4599. 31601813 10.1038/s41467-019-12534-6 PMC6787074

[21] C. Zhou , X. Li , Z. Gong , C. Jia , Y. Lin , C. Gu , G. He , Y. Zhong , J. Yang , X. Guo , Nat. Commun. 2018, 9, 807. 29476061 10.1038/s41467-018-03203-1 PMC5825177

[22] Y. Han , C. Nickle , Z. Zhang , H. P. A. G. Astier , T. J. Duffin , D. Qi , Z. Wang , E. Del Barco , D. Thompson , C. A. Nijhuis , Nat. Mater. 2020, 19, 843. 32483243 10.1038/s41563-020-0697-5

[23] B. H. Farnum , K.‐R. Wee , T. J. Meyer , Nat. Chem. 2016, 8, 845. 27554411 10.1038/nchem.2536

[24] H. Tian , J. Oscarsson , E. Gabrielsson , S. K. Eriksson , R. Lindblad , B. Xu , Y. Hao , G. Boschloo , E. M. J. Johansson , J. M. Gardner , A. Hagfeldt , H. Rensmo , L. Sun , Sci. Rep. 2014, 4, 4282. 24603319 10.1038/srep04282 PMC3945487

[25] B. O'regan , M. Grätzel , Nature 1991, 353, 737.

[26] Y. Ren , D. Zhang , J. Suo , Y. Cao , F. T. Eickemeyer , N. Vlachopoulos , S. M. Zakeeruddin , A. Hagfeldt , M. Grätzel , Nature 2023, 613, 60. 36288749 10.1038/s41586-022-05460-z

[27] E. Benazzi , J. Mallows , G. H. Summers , F. A. Black , E. A. Gibson , J. Mater. Chem. C 2019, 7, 10409.

[28] I. R. Perera , T. Daeneke , S. Makuta , Z. Yu , Y. Tachibana , A. Mishra , P. Bäuerle , C. A. Ohlin , U. Bach , L. Spiccia , Angew. Chem., Int. Ed. 2015, 54, 3758. 10.1002/anie.201409877 25631105

[29] T. Daeneke , Z. Yu , G. P. Lee , D. Fu , N. W. Duffy , S. Makuta , Y. Tachibana , L. Spiccia , A. Mishra , P. Bäuerle , U. Bach , Adv. Energy Mater. 2015, 5, 1401387.

[30] C. J. Wood , G. H. Summers , E. A. Gibson , Chem. Commun. 2015, 51, 3915. 10.1039/c4cc10230d 25658068

[31] J. J. Leung , J. Warnan , D. H. Nam , J. Z. Zhang , J. Willkomm , E. Reisner , Chem. Sci. 2017, 8, 5172. 28970903 10.1039/c7sc01277b PMC5618793

[32] T. E. Rosser , M. A. Gross , Y.‐H. Lai , E. Reisner , Chem. Sci. 2016, 7, 4024. 30155045 10.1039/c5sc04863j PMC6013811

[33] F. Li , K. Fan , B. Xu , E. Gabrielsson , Q. Daniel , L. Li , L. Sun , J. Am. Chem. Soc. 2015, 137, 9153. 26132113 10.1021/jacs.5b04856

[34] J. He , H. Lindström , A. Hagfeldt , S.‐E. Lindquist , J. Phys. Chem. B 1999, 103, 8940.

[35] L. D'Amario , L. J. Antila , B. Pettersson Rimgard , G. Boschloo , L. Hammarström , J. Phys. Chem. Lett. 2015, 6, 779. 26262652 10.1021/acs.jpclett.5b00048

[36] S. Mori , S. Fukuda , S. Sumikura , Y. Takeda , Y. Tamaki , E. Suzuki , T. Abe , J. Phys. Chem. C 2008, 112, 16134.

[37] S. Nakade , Y. Saito , W. Kubo , T. Kitamura , Y. Wada , S. Yanagida , J. Phys. Chem. B 2003, 107, 8607.

[38] F. Odobel , Y. Pellegrin , E. A. Gibson , A. Hagfeldt , A. L. Smeigh , L. Hammarström , Coord. Chem. Rev. 2012, 256, 2414.

[39] A. Morandeira , G. Boschloo , A. Hagfeldt , L. Hammarström , J. Phys. Chem. B 2005, 109, 19403. 16853506 10.1021/jp053230e

[40] A. Nattestad , M. Ferguson , R. Kerr , Y.‐B. Cheng , U. Bach , Nanotechnology 2008, 19, 295304. 21730603 10.1088/0957-4484/19/29/295304

[41] F. G. L. Parlane , C. Mustoe , C. W. Kellett , S. J. Simon , W. B. Swords , G. J. Meyer , P. Kennepohl , C. P. Berlinguette , Nat. Commun. 2017, 8, 1761. 29176734 10.1038/s41467-017-01726-7 PMC5701207

[42] S. J. C. Simon , F. G. L. Parlane , W. B. Swords , C. W. Kellett , C. Du , B. Lam , R. K. Dean , K. Hu , G. J. Meyer , C. P. Berlinguette , J. Am. Chem. Soc. 2016, 138, 10406. 27518595 10.1021/jacs.6b06288

[43] F. A. Black , C. A. Clark , G. H. Summers , I. P. Clark , M. Towrie , T. Penfold , M. W. George , E. A. Gibson , Phys. Chem. Chem. Phys. 2017, 19, 7877. 28262897 10.1039/c6cp05712h

[44] Y. Uemura , T. N. Murakami , N. Koumura , J. Phys. Chem. C 2014, 118, 16749.

[45] W. B. Swords , S. J. C. Simon , F. G. L. Parlane , R. K. Dean , C. W. Kellett , K. Hu , G. J. Meyer , C. P. Berlinguette , Angew. Chem., Int. Ed. 2016, 55, 5956. 10.1002/anie.201510641 27060916

[46] T. Bouwens , S. Mathew , J. N. H. Reek , Faraday Discuss. 2019, 215, 393. 30951057 10.1039/c8fd00169c

[47] T. Bouwens , T. M. A. Bakker , K. Zhu , J. Hasenack , M. Dieperink , A. M. Brouwer , A. Huijser , S. Mathew , J. N. H. Reek , Nat. Chem. 2023, 15, 213. 36302868 10.1038/s41557-022-01068-y

[48] V. V. Pavlishchuk , A. W. Addison , Inorg. Chim. Acta 2000, 298, 97.

[49] N. G. Connelly , W. E. Geiger , Chem. Rev. 1996, 96, 877. 11848774 10.1021/cr940053x

[50] P. Thordarson , Chem. Soc. Rev. 2011, 40, 1305. 21125111 10.1039/c0cs00062k

[51] P. Qin , J. Wiberg , E. A. Gibson , M. Linder , L. Li , T. Brinck , A. Hagfeldt , B. Albinsson , L. Sun , J. Phys. Chem. C 2010, 114, 4738.

[52] K. Zhu , S. K. Frehan , A. M. Jaros , D. B. O'neill , J. P. Korterik , K. Wenderich , G. Mul , A. Huijser , J. Phys. Chem. C 2021, 125, 16049. 10.1021/acs.jpcc.1c03553 PMC8411848 34484551

[53] K. Zhu , S. K. Frehan , G. Mul , A. Huijser , J. Am. Chem. Soc. 2022, 144, 11010. 35675488 10.1021/jacs.2c04301 PMC9228059

[54] Q. Pan , L. Freitag , T. Kowacs , J. C. Falgenhauer , J. P. Korterik , D. Schlettwein , W. R. Browne , M. T. Pryce , S. Rau , L. González , J. G. Vos , A. Huijser , Chem. Commun. 2016, 52, 9371. 10.1039/c6cc05222c 27367442

[55] A. Trabolsi , N. Khashab , A. C. Fahrenbach , D. C. Friedman , M. T. Colvin , K. K. Cotí , D. Benítez , E. Tkatchouk , J.‐C. Olsen , M. E. Belowich , R. Carmielli , H. A. Khatib , W. A. Goddard , M. R. Wasielewski , J. F. Stoddart , Nat. Chem. 2010, 2, 42. 21124379 10.1038/nchem.479

[56] K. Saito , A. W. Rutherford , H. Ishikita , Proc. Natl. Acad. Sci. USA 2013, 110, 954. 23277574 10.1073/pnas.1212957110 PMC3549079

[57] C. J. Wood , G. H. Summers , C. A. Clark , N. Kaeffer , M. Braeutigam , L. R. Carbone , L. D'amario , K. Fan , Y. Farré , S. Narbey , F. Oswald , L. A. Stevens , C. D. J. Parmenter , M. W. Fay , A. La Torre , C. E. Snape , B. Dietzek , D. Dini , L. Hammarström , Y. Pellegrin , F. Odobel , L. Sun , V. Artero , E. A. Gibson , Phys. Chem. Chem. Phys. 2016, 18, 10727. 26734947 10.1039/c5cp05326a

[58] L. D'amario , J. Föhlinger , G. Boschloo , L. Hammarström , Chem. Sci. 2018, 9, 223. 29629091 10.1039/c7sc03442c PMC5869301

[59] H. Tian , Z. Yu , A. Hagfeldt , L. Kloo , L. Sun , J. Am. Chem. Soc. 2011, 133, 9413. 21591709 10.1021/ja2030933

[60] A. Yella , S. Mathew , S. Aghazada , P. Comte , M. Grätzel , M. K. Nazeeruddin , J. Mater. Chem. C 2017, 5, 2833.

[61] J. J. Nelson , T. J. Amick , C. M. Elliott , J. Phys. Chem. C 2008, 112, 18255.

[62] F. Fabregat‐Santiago , G. Garcia‐Belmonte , I. Mora‐Seró , J. Bisquert , Phys. Chem. Chem. Phys. 2011, 13, 9083. 21468446 10.1039/c0cp02249g

[63] Z. Huang , G. Natu , Z. Ji , P. Hasin , Y. Wu , J. Phys. Chem. C 2011, 115, 25109.

[64] R. D. Shannon , Acta Crystallogr. A 1976, 32, 751.

[65] J. Halme , P. Vahermaa , K. Miettunen , P. Lund , Adv. Mater. 2010, 22, E210. 20717984 10.1002/adma.201000726

