# Supplementary material for: Rotaxane‐Functionalized Dyes for Charge‐Rectification in p‐Type Photoelectrochemical Devices
Source: Adv Sci (Weinh). 2023 Dec 18;11(9):2306032. doi: 10.1002/advs.202306032 (PMC10916627; doi:10.1002/advs.202306032)
Supplement: Supplementary file 1 — Supporting Information [file ADVS-11-2306032-s002.pdf]

ADVANCED SCIENCE
Open Access

Supporting Information

for *Adv. Sci.*, DOI 10.1002/advs.202306032

Rotaxane-Functionalized Dyes for Charge-Rectification in *p*-Type Photoelectrochemical Devices

*Tessel Bouwens, Tijmen M. A. Bakker, Kaijian Zhu, Annemarie Huijser, Simon Mathew and Joost N. H. Reek**

Supplementary Materials for

Rotaxane-functionalized Dyes for Charge-Rectification in p-Type photoelectrochemical Devices

T. Bouwens[1], T. M. A. Bakker[1], K. Zhu[2], A. Huijser[2], S. Mathew[1], J. N. H. Reek[1]*

[1] *van 't Hoff Institute for Molecular Sciences, Universiteit van Amsterdam, Science Park 904, 1098 XH Amsterdam, The Netherlands*

[2] *PhotoCatalytic Synthesis Group, MESA+ Institute for Nanotechnology, University of Twente, P.O. Box 217, 7500 AE Enschede, The Netherlands*

Email: j.n.h.reek@uva.nl

Contents

S1. Materials and methods 3

S1.1. General 3

S1.2. Synthesis 4

S1.3. NMR spectra 10

S1.4. ESI–HRMS spectra 16

S1.5. Binding studies 21

S1.6. Dye adsorption onto NiO 25

S1.7. Differential Pulse Voltammogram $P_{Rotaxane}$+ $P_{Stopper}$ 27

S1.8. Spectroelectrochemistry 28

S2. Ultrafast spectroscopy experiments 29

S2.1. Experimental details 29

S2.2. Ultrafast spectroscopy results and discussion 31

S3. Device Fabrication and Characterization 42

S3.1. DSSC device Assembly 42

S3.2. DSSC Assembly Employing I^-/I_3^- 42

S3.3. DSSC Assembly Employing 3-NDI-ring Electrolyte 42

S3.4. DSSC Assembly Employing 3-NDI-thread Electrolyte 43

S3.5. DSSC Characterization 43

S3.6. Photovoltaic Performance Employing I^-/I_3^-***Electrolyte*** 44

S3.7. Electrochemical Impedance Spectroscopy on DSSCs employing I^-/I_3^- 48

S3.8. Characterization DSSCs Employing 3-NDI-ring 50

S3.9. Electrochemical Impedance Spectroscopy on DSSCs Employing 3-NDI-ring 51

S3.10. Calculation of limiting photocurrent 53

S3.11. Investigation of diffusion limitation with the 3-NDI-based electrolyte 55

S3.12. Comparison IPCE set-up and solar simulator 58

S4. References 59

S1. Materials and methods

S1.1. General

Commercially reagents and solvents were obtained Sigma-Aldrich and Fluorochem and were used without purification. Compounds: **3-NDI-ring**,[1] **DNP** precursor **3**,[2] dye precursor **5**,[3] and stopper **6**[4] were synthesized according to literature procedures**.** Conducting FTO glass was obtained from Sigma-Aldrich (L × W × D 300 × 300 × 2.3 mm, surface resistivity 15 Ω/square). All reactions were carried out under nitrogen in flame dried glassware. MeCN, DCM, and THF were dried by a solvent purification system. Thin layer chromatography was performed on Merck KGaA aluminum plates pre-coated TLC silica gel 60 F_{254} and analyzed with UV light (254 nm and 365 nm). Column chromatography was performed using silica gel (SiliCycle, SiliaFlash P60, 40–63 μm, 230–400 mesh). NMR analysis was performed on a Bruker AV300, AV400 and AV500 spectrometer and are reported in ppm using a solvent residual signal as internal standard (7.26 ppm for $CDCl_3$; 5.32 ppm for CD_2Cl_2 and 2.05 ppm for $(CD_3)_2CO$). Exact mass of the compounds was obtained on an AccuTOF GC v 4g, JMS-T100GCV Mass spectrometer (JEOL, Japan) equipped with an FD Emitter, Carbotec or Linden (Germany), FD 13 μm. Current rate 51.2 mA min^{-1} over 1.2 min machine using field desorption (FD) as ionization method. UV–Vis measurements were performed on a single beam Hewlett Packard 8453 spectrometer in a 10 mm path length quartz cuvette using MeCN as background. Electrochemistry experiments were performed on a PGSTAT302N potentiostat from Autolab with a glassy carbon working electrode (MetrOhm, diameter 3 mm), a leakless Ag/AgCl reference electrode (eDAQ, ET069) and a Pt wire counter electrode. The 0.5 mM analyte solution contained 100 mM $TBAPF_6$ as supporting electrolyte and was prepared in absence of air. To all samples, ferrocene/ferrocenium (Fc/Fc^+) was added as an internal redox standard to determine the redox potentials versus NHE, $E_{1/2}$ Fc/Fc^+ = 630 mV in MeCN[5] and 700 mV in DCM[6].

S1.2. Synthesis

The terminal alkyne is introduced via a Knoevenagel condensation to install the rotaxane stoppers through click chemistry with stopper-azide **2**. The subsequent click reaction to obtain $\mathbf{P_{Stopper}}$ was performed with $Cu^{I}(OAc)(PPh_3)_2$ as a catalyst (20 mol%; 2 μmol) in DCM/THF at ambient temperature in 58.1% yield. The disappearance of the characteristic alkyne peak at 2.9 ppm confirmed the reaction with the reactive azide-group. The synthesis of $\mathbf{P_{Rotaxane}}$ was performed similarly in a 40.7% yield, with additional cooling of the reaction mixture to -15°C for 10 minutes to enforce complexation of the **3-NDI-ring** to the **DNP**-arm of compound **1** prior to the initiation of the click reaction by addition of $Cu(OAc)(PPh_3)_2$. We observed that the sole rotaxane product isolated featured one **3-NDI-ring** based on ^{1}H NMR integration (Figure S3) and HRMS (ESI–MS, Figures S16–17, both positive and negative modes). No HRMS signal at *m/z* = 3739.47 was observed (i.e., correlating to the 2×**3-NDI-ring** bisrotaxane). While the 2 ring bisrotaxane product could be present in the crude reaction mixture, we solely isolated $\mathbf{P_{Rotaxane}}$ by column chromatography as its solubility differs greatly from the parent dye upon rotaxane formation.

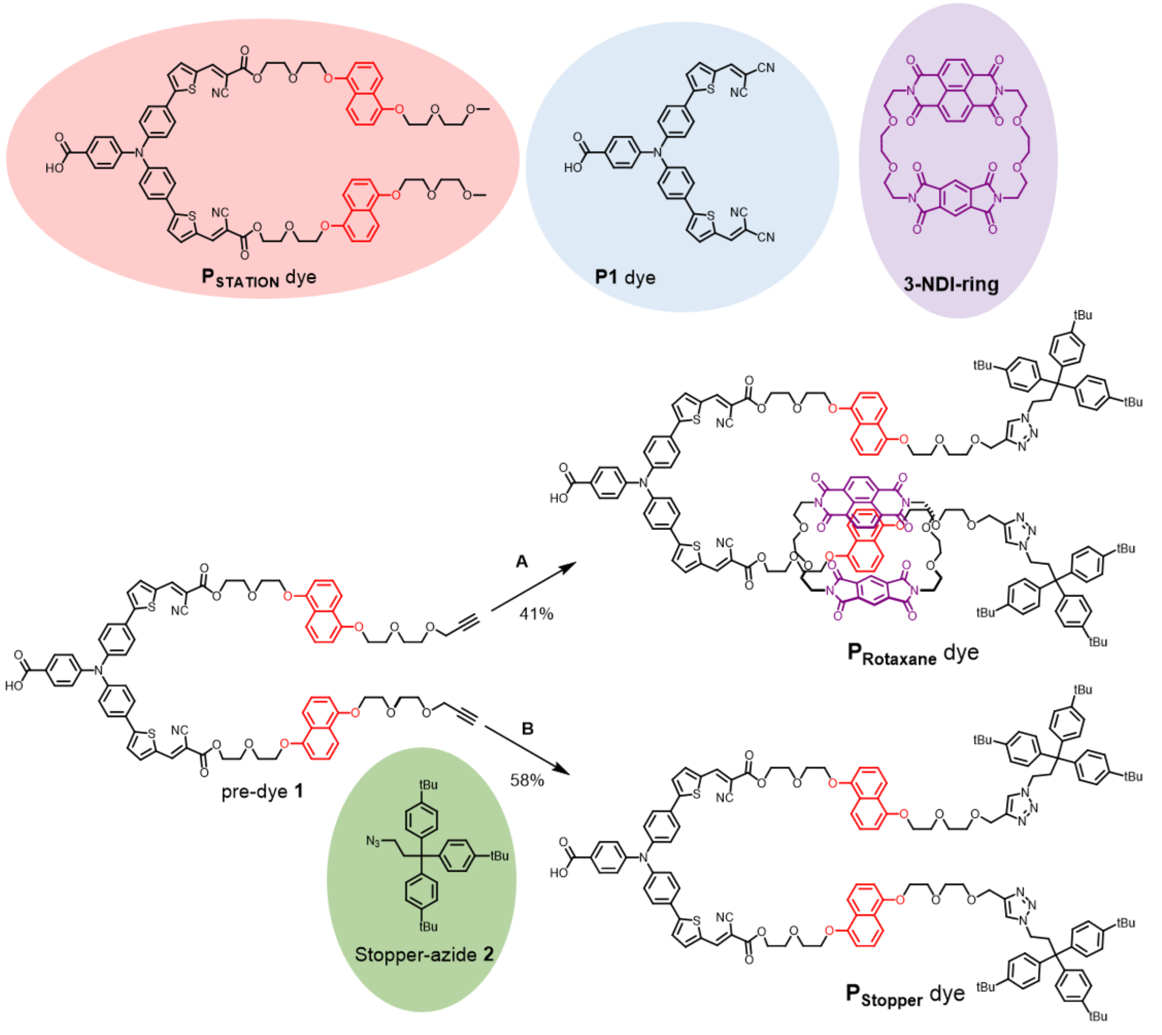

Scheme S1. *Molecular structures of the benchmark dye* ***P1****, the macrocyclic redox mediator* ***3-NDI-ring*** *and the* ***P$_{STATION}$*** *used in previous studies. On the right the synthesis of* ***P$_{Rotaxane}$*** *and* ***P$_{Stopper}$*** *via click chemistry from compound* ***1****. A = 1)* ***3-NDI-ring*** *(6.2 equiv.), Stopper-azide* ***2*** *(2.3 equiv.), 2) Lutidine Cu(OAc)(PPh$_3$)$_2$, in DCM/THF (2:3) at -15°C. B) Stopper-azide* ***2*** *(2.1 equiv.), Lutidine, Cu(OAc)(PPh$_3$)$_2$, in DCM/THF (2:3) at ambient temperature (20°C).*

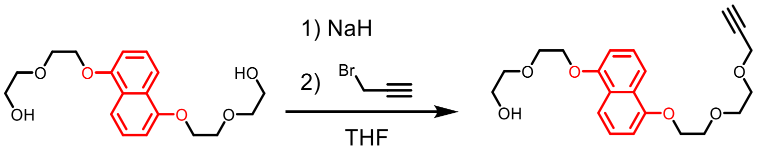

Compound ***4***. Compound **4** was synthesized based on a synthesis procedure by Stoddart and coworkers.[7] Compound **3** (1.00 g, 2.97 mmol, 1 equiv.) was dissolved in dry THF (20 mL). Then, NaH in mineral oil was added (60% w/w, 131mg, 3.27 mmol, 1.1 equiv.) and bubbles emerged. The mixture was heated at reflux for 1 hour and a white precipitate was obtained. The mixture was cooled to 45°C and propagyl bromide was added (80% w/w in toluene, 364 mL, 0.389 mg propagyl bromide, 1.1 equiv.). The mixture was stirred for 16 hours at 45°C. MeOH was added until most precipitate was dissolved. The mixture was filtered to remove NaBr and organic solvents were removed by rotary evaporation. The yellow was dissolved in ethyl acetate (100 mL) and washed with water (3 × 75 mL) to remove remaining salts. The organic layer was dried with $MgSO_4$. After filtration the ethyl acetate was removed. The yellow oil was purified by column chromatography (silica, DCM/Ethyl acetate, 1:2). Compound **4** was obtained as the second fraction as a yellow waxy solid (0.478 mg, 43%). ^{1}H NMR (300 MHz, $(CD_3)_2CO$) δ 7.84 (dd, 2H), 7.37 (dt, 2H), 6.99 (dd, 2H), 4.35 – 4.27 (m, 4H), 4.20 (d, J = 2.4 Hz, 2H), 4.02 – 3.92 (m, 4H), 3.81 – 3.74 (m, 2H), 3.73 – 3.63 (m, 6H), 2.93 (t, J = 2.4 Hz, 1H), . ^{13}C NMR (75 MHz, $(CD_3)_2CO$) δ 155.32, 127.60, 126.03, 115.11, 106.56, 106.52, 75.79, 73.78, 71.30, 70.36, 70.31, 69.85, 68.84, 61.89, 58.61, 31.52, 31.26, 31.00, 30.61, 30.35, 30.10, 29.84, 29.58, 29.50, 29.33, 29.07, 28.78, 28.52. HRMS (FD–MS) (*m/z*): $[M]^+$ $C_{24}H_{27}N_1O_7$ calculated 374.1729; found 374.1737.

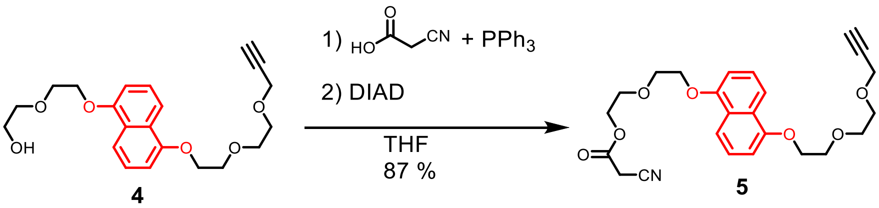

Compound ***5***. Compound **4** (406 mg, 1.09 mmol 1 equiv.) and cyanoacetic acid (85.06, 96.9 mmol, 1.05 equiv.) and catalytic amount of DMAP (~2 mg) were dissolved in dry DCM (15 mL). Then, DCC (235 mg, 1.14 mmol, 1.05 equiv.) was added and formation of DCU was observed by formation of a precipitate. The mixture was stirred over night at ambient temperature. The DCU solids were filtered off and the organic solvent was removed by rotary evaporation. The yellow oil was purified by column chromatography (silica, DCM/EtOAc, 4:1). Compound **5** was obtained as an off-white waxy solid (419 mg, 87%). ^{1}H NMR (400 MHz,

$CDCl_3$) δ 7.88 (ddt, J = 19.3, 8.5, 0.9 Hz, 2H), 7.38 (ddd, J = 8.5, 7.6, 3.0 Hz, 2H), 6.88 (ddd, J = 7.7, 2.7, 0.9 Hz, 2H), 4.46 – 4.39 (m, 2H), 4.37 – 4.28 (m, 4H), 4.25 (d, J = 2.4 Hz, 2H), 4.03 (q, J = 4.8 Hz, 4H), 3.95 – 3.80 (m, 4H), 3.83 – 3.66 (m, 2H), 3.41 (s, 2H), 2.45 (t, J = 2.4 Hz, 1H). ^{13}C NMR (126 MHz, CD_2Cl_2) δ 163.85, 157.63, 154.97, 154.83, 125.81, 125.71, 115.06, 114.79, 113.72, 106.30, 106.27, 80.36, 74.67, 71.25, 70.37, 70.32, 70.27, 69.85, 69.40, 68.62, 68.56, 66.40, 58.85, 54.43, 54.22, 54.07, 54.00, 53.78, 53.57, 49.87, 34.29, 26.23, 26.14, 25.50, 25.30. HRMS (FD–MS) (*m/z*): $[M]^+$ $C_{24}H_{27}N_1O_7$ calculated 441.1788; found 441.1781.

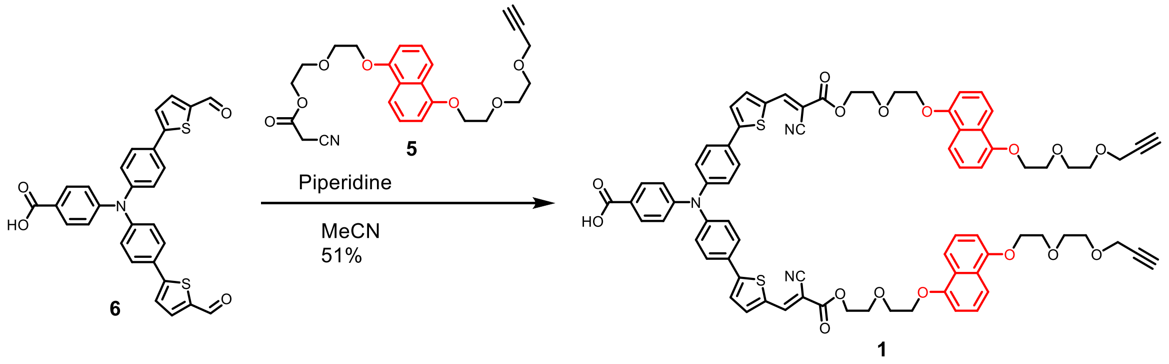

Precursor dye ***1***. Compound **6** (425 mg; 0.962 mmol; 3.5 equiv.) and compound **5** (140 mg; 0.275 mmol; 1 equiv.) and piperidine (5 drops) were dissolved in MeCN (50 mL) and heated at reflux for 4 hours. The MeCN was evaporated, and water was added. The water was acidified with HCl (1 M) until pH 3.5 was obtained. The organic compounds were extracted with DCM (3 × 100 mL). After drying the organic layers with Na_2SO_4 and filtration, the product was purified by column chromatography (silica, DCM/MeOH/AcOH, 98:2:0.002). The red fractions containing product were checked for their purity by MS and pure fractions were combined to yield the product **1** as a waxy red solid (191 mg, 51%). TLC (DCM/MeOH, 95:5): R_f = 0.58; ^{1}H NMR (300 MHz, $CDCl_3$) δ 8.21 (s, 1H), 8.00 (d, J = 8.7 Hz, 1H), 7.85 (t, J = 7.7 Hz, 3H), 7.71 – 7.61 (m, 3H), 7.43 – 7.28 (m, 4H), 7.18 (dd, J = 10.8, 8.6 Hz, 3H), 6.83 (dd, J = 15.1, 7.7 Hz, 2H), 4.50 (t, J = 4.7 Hz, 2H), 4.29 (dt, J = 14.3, 4.8 Hz, 5H), 4.21 (d, J = 2.4 Hz, 2H), 4.05 (t, J = 4.7 Hz, 2H), 3.97 (q, J = 4.2, 3.5 Hz, 5H), 3.87 – 3.78 (m, 2H), 3.77 – 3.71 (m, 3H), 3.49 (s, 1H), 2.42 (t, J = 2.3 Hz, 1H). ^{13}C NMR (75 MHz, $(CD_3)_2CO$) δ 167.05, 163.35, 155.32, 155.29, 154.27, 151.53, 148.52, 147.34, 141.67, 135.43, 132.17, 129.44, 128.67, 127.60, 126.24, 126.11, 126.02, 125.86, 125.33, 123.70, 116.55, 115.18, 115.14, 106.57, 106.53, 98.34, 80.93, 75.79, 71.32, 70.38, 69.88, 69.69, 68.83, 68.79, 66.11, 58.64, 29.84. HRMS (ESI–MS) (*m/z*): $[(\mathbf{1})Na]^+$ calculated 1378.4011; found 1378.4027, $[(\mathbf{1})K]^+$ calculated 1394.3751, found 1394.3779.

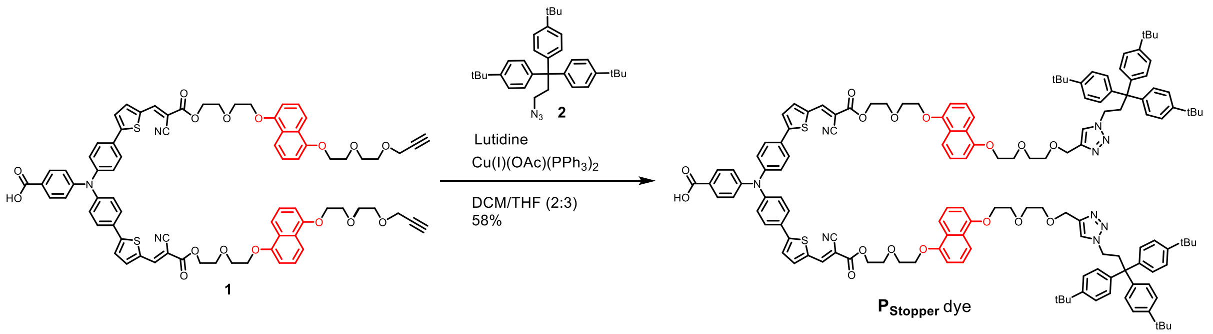

P

***P$_{Stopper}$* *dye*.** Compound **1** (36.5 mg, 26.9 µmol, 1 equiv.), and Stopper-azide **2** (27.2 mg, 56.5 µmol 2.1 equiv.) are dissolved in DCM (4.5 mL, degassed, not dry) and THF (3 mL, degassed, not dry). Then the Cu(I)(OAc)(PPh_3)$_2$ catalyst (20 mol%; 2.7 µmol, 3.5 mg) with lutidine (3 drops) was added and was stirred overnight at room temperature. After 1 night the TLC (DCM/MeOH, 97.5:2.5) shows a new spot. After removing the organic solvents. 20 mL DCM was added to redissolve the red film. To remove the Cu(I)(OAc)(PPh_3)$_2$ catalyst EDTA solution (20 mL, 0.5 M, pH 8) was added and the biphasic mixture was stirred for 1 hour. This EDTA solution (0.5 M, 1 L) consisted of EDTA·2H_2O (186.1 g) and NaOH (~20 g) was added until pH 8 was obtained. The aqueous layer was removed and the EDTA washing step was repeated. The organic layer was dried (Na_2SO_4), filtered, the solvents removed, and the mixture purified by column chromatography (silica, DCM/MeOH/AcOH, 98:2:0.002). Red fractions containing product were checked for their purity by MS and pure fractions were combined to yield the product **P$_{Stopper}$** as a fine red solid (36.3 mg, 58.1%). TLC (DCM/MeOH, 95:5): R_f = 0.5; ^{1}H NMR (500 MHz, CD_2Cl_2) δ 8.24 (s, 2H), 7.99 (d, *J* = 8.4 Hz, 2H), 7.83 (dd, *J* = 18.9, 9.4 Hz, 4H), 7.71 – 7.63 (m, 8H), 7.42 – 7.38 (m, 2H), 7.38 – 7.33 (m, 4H), 7.32 (d, *J* = 8.4 Hz, 12H) 7.24 (d, *J* = 8.3, 12H), 7.23 – 7.08 (m, 6H), 6.91 – 6.77 (m, 4H), 4.63 (s, 2H), 4.49 (dt, *J* = 6.4, 3.2 Hz, 4H), 4.31 (dt, *J* = 12.2, 4.6 Hz, 4H), 4.24 (dt, *J* = 8.5, 4.6 Hz, 4H), 4.19 (d, *J* = 2.4 Hz, 2H), 4.03 (q, *J* = 9.8, 7.6 Hz, 8H), 3.95 (p, *J* = 3.9, 3.4 Hz, 8H), 3.76 (q, *J* = 4.8 Hz, 4H), 3.70 (dd, *J* = 5.8, 3.1 Hz, 4H), 1.31 (s, 54H). ^{13}C NMR (101 MHz, $(CD_3)_2CO$) δ 167.43, 163.72, 155.68, 155.66, 154.60, 151.89, 149.95, 148.85, 147.67, 145.92, 144.86, 142.01, 135.77, 132.56, 129.79, 129.05, 127.97, 126.59, 126.49, 126.41, 126.30, 126.18, 125.72, 124.24, 124.09, 116.94, 115.57, 115.55, 106.95, 106.92, 98.71, 71.89, 70.79, 70.74, 70.06, 69.21, 69.18, 66.48, 65.51, 55.49, 48.65, 41.59, 35.26, 32.03. 1.41. HRMS (ESI–MS) (*m/z*): = [(**P$_{stopper}$**)Na]$^+$ calculated 2342.0958; found 2342.0996, [(**P$_{Stopper}$**)K]$^+$ calculated 2358.0697; found 2358.0784, [(**P$_{stopper}$**)CH_3CNNa]$^+$ calculated 2383.1223, found 2383.0408.

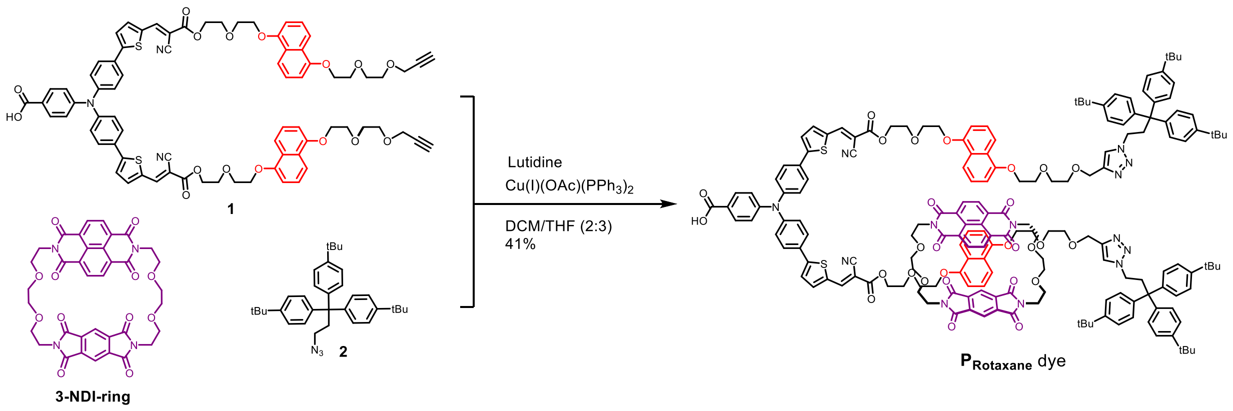

P

$P_{Rotaxane}$ dye. Compound **1** (13 mg, 9.6 µmol, 1 equiv.), **3-NDI-ring** (42.5 mg, 60 µmol 6.2 equiv.) and stopper **2** (11 mg, 22 µmol, 2.3 equiv.) are dissolved in DCM (1.5 mL, degassed, not dry) mL THF (1 mL, degassed, not dry). The mixture was cooled down to -15°C and after 10 minutes the Cu(I)(OAc)(PPh_3)$_2$ catalyst (20 mol%; 2 µmol, 1.3 mg) with of lutidine (1 drop) was added. The dark red mixture was stirred overnight while the salt-ice bath melted. After 16 hours, the TLC (DCM:MeOH, 95:5 v/v) shows a new spot (R_f= 0.44). After removing the organic solvents, DCM (25 mL) was added to redissolve the red film. To remove the Cu(I)(OAc)(PPh_3)$_2$ catalyst EDTA solution (25 mL, 0.5 M, pH 8) was added and the biphasic mixture was stirred for 1 hour. The aqueous layer was removed and the EDTA washing step was repeated. The organic layer was dried (Na_2SO_4), filtered, the solvents removed, and the mixture purified by column chromatography (silica, DCM/MeOH/AcOH, 98%:2%:0.002%). The red fractions containing product were checked for their purity by MS and pure fractions were combined to yield the product **$P_{Rotaxane}$** as a red, flaky solid (11.8 mg,40.7%). TLC (DCM:MeOH, 95:5 v/v): R_f = 0.44; ^{1}H NMR (500 MHz, CD_2Cl_2) δ 8.34 (s, 1H), 8.24 (s, 2H), 8.20 (d, *J* = 15.3 Hz, 4H), 7.98 (d, *J* = 8.3 Hz, 2H), 7.82 (dd, *J* = 19.8, 8.8 Hz, 2H), 7.76 – 7.69 (m, 2H), 7.69 – 7.61 (m, 2H), 7.60 – 7.50 (m, 2H), 7.44 –7.27 (m, 14H), 7.26 – 7.10 (m, 22H), 7.07 (d, *J* = 4.6 Hz, 2H), 6.83 (dd, *J* = 19.5, 7.7 Hz, 2H), 6.80 – 6.73 (m, 2H), 6.63 (d, *J* = 18.6 Hz, 2H), 6.31 – 6.23 (m, 2H), 4.74 (m, 4H), 4.62 (s, 2H), 4.50 – 4.44 (m, 2H), 4.29 (t, *J* = 4.7 Hz, 2H), 4.23 (t, *J* = 4.9 Hz, 2H), 4.12 – 4.10 (m, 4H), 4.07 – 3.98 (m, 8H), 3.98 – 3.86 (m, 20H), 3.86 – 3.72 (m, 16H), 3.71 – 3.58 (m, 8H), 1.29 (s, 54H). ^{13}C NMR (126 MHz, CD_2Cl_2) δ 166.71, 149.67, 143.90, 134.99, 134.05, 133.96, 130.38, 130.22, 130.12, 129.02, 128.90, 125.82, 125.79, 125.62, 125.59, 125.29, 114.78, 71.44, 70.70, 70.28, 68.54, 54.00, 40.94, 34.80, 34.71, 31.64, 31.61. HRMS (ESI–MS) (*m/z*): = [(**$P_{Rotaxane}$**)Na]$^+$ calculated 3053.2845; found 3053.2826, [(**$P_{Rotaxane}$**-H]$^-$ calculated 3029.2869, found 3029.2895, [(**$P_{Rotaxane}$**-H)CH_2Cl_2]$^-$ calculated 3065.2625; found 3065.2632.

S1.3. NMR spectra

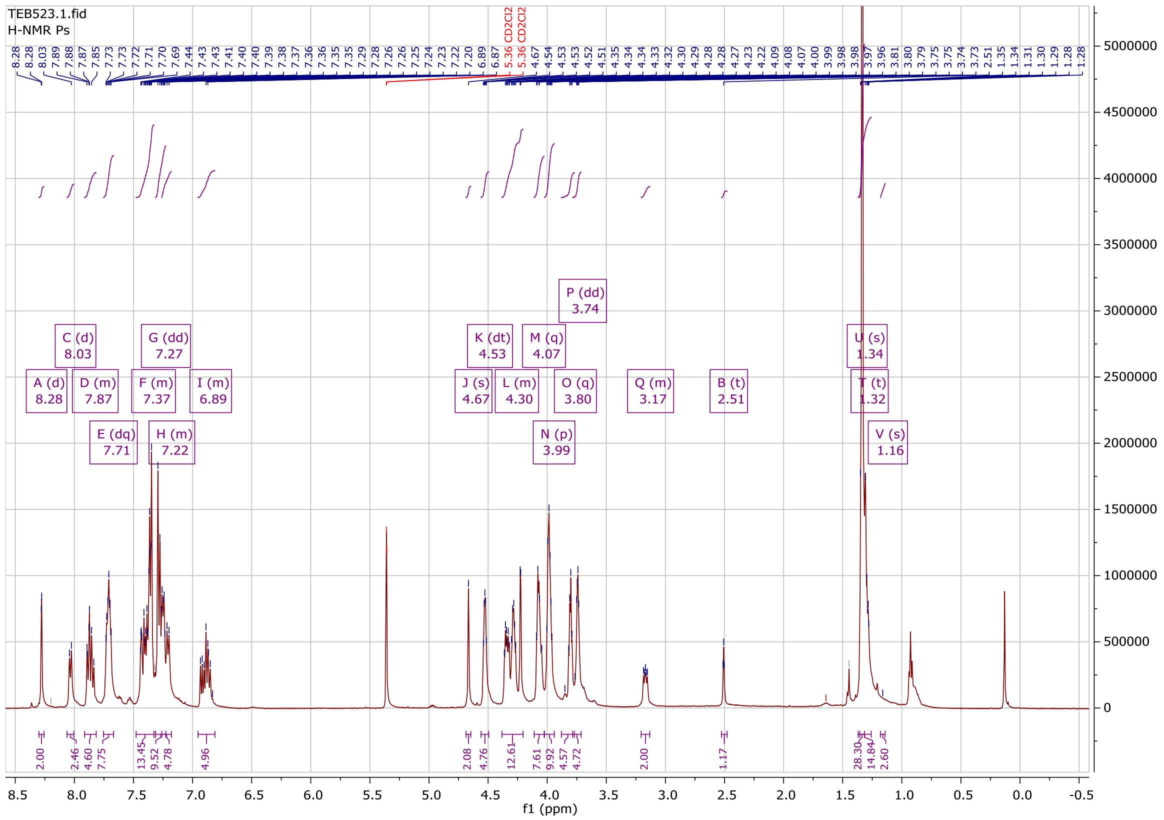

Figure S1. *^{1}H NMR spectrum of final product* $\boldsymbol{P_{Stopper}}$ *(400 MHz, 298 K) in* $CDCl_3$*.*

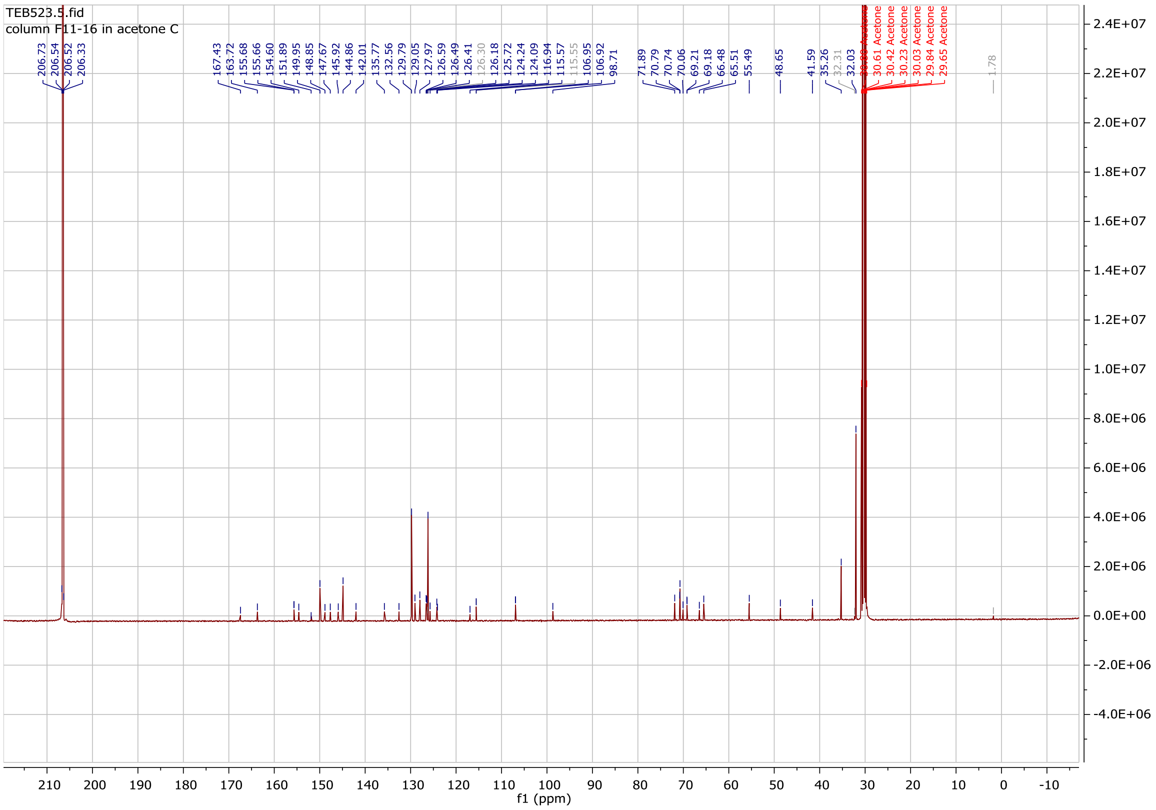

Figure S2. *^{13}C NMR spectrum of final product* $\boldsymbol{P_{Stopper}}$ *(500 MHz, 298 K) in $(CD_3)_2CO$.*

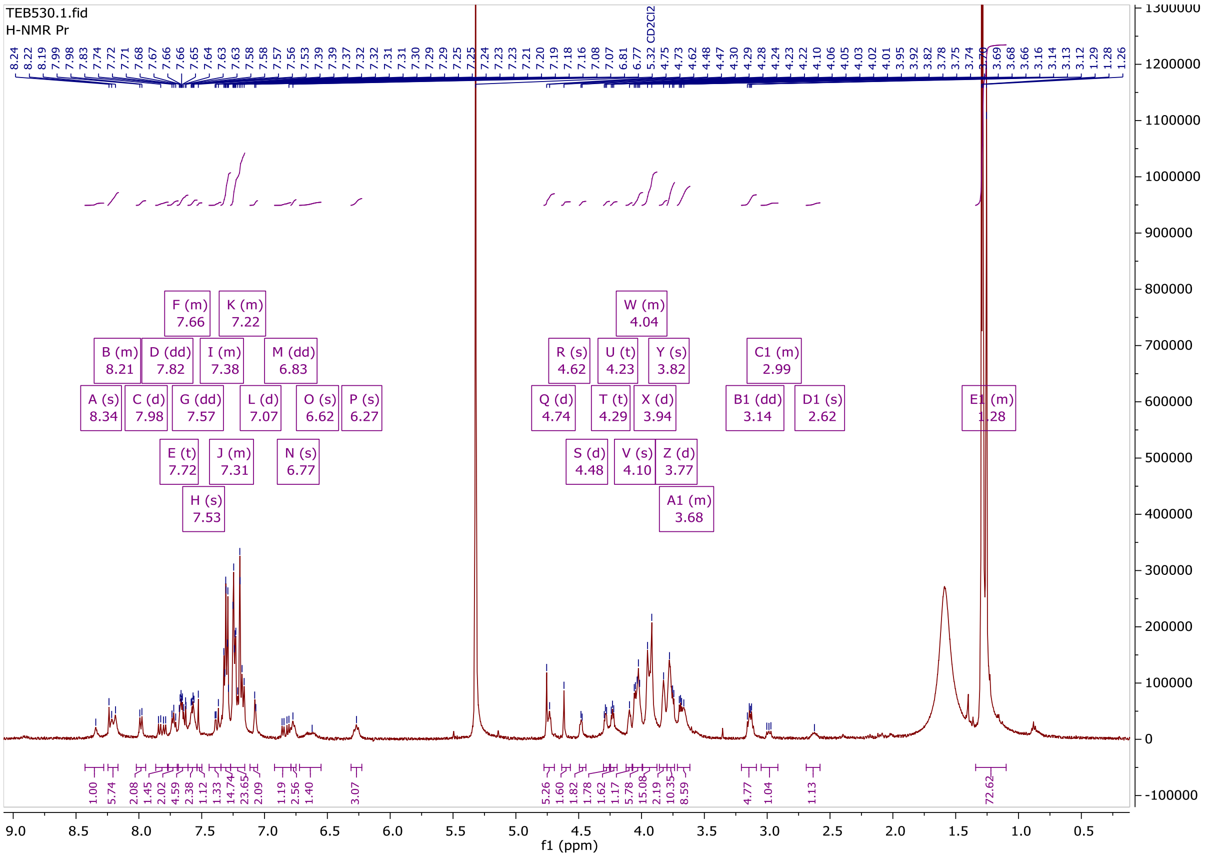

Figure S3. *^{1}H NMR spectrum of final product* $\boldsymbol{P_{Rotaxane}}$ *(500 MHz, 298 K) in CD_2Cl_2.*

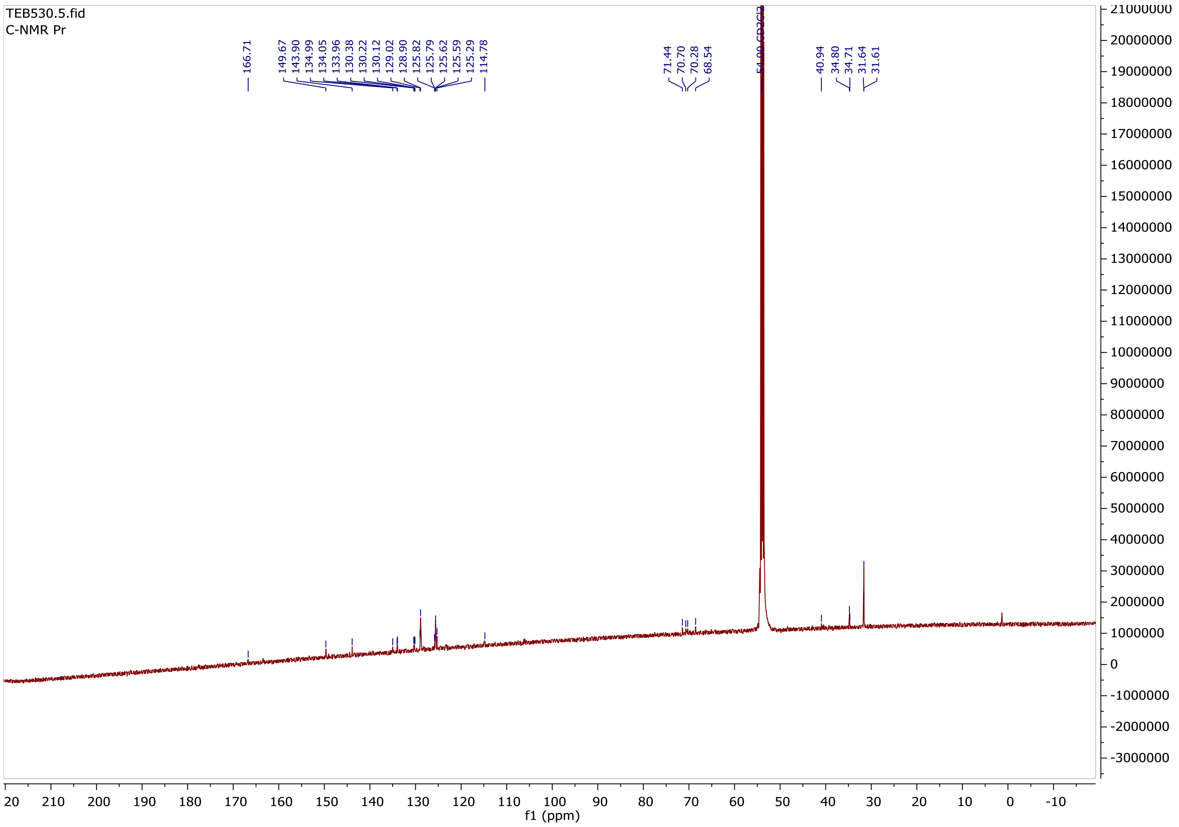

Figure S4. *^{13}C NMR spectrum of final product* ***P*****Rotaxane** *(500 MHz, 298 K) in CD_2Cl_2.*

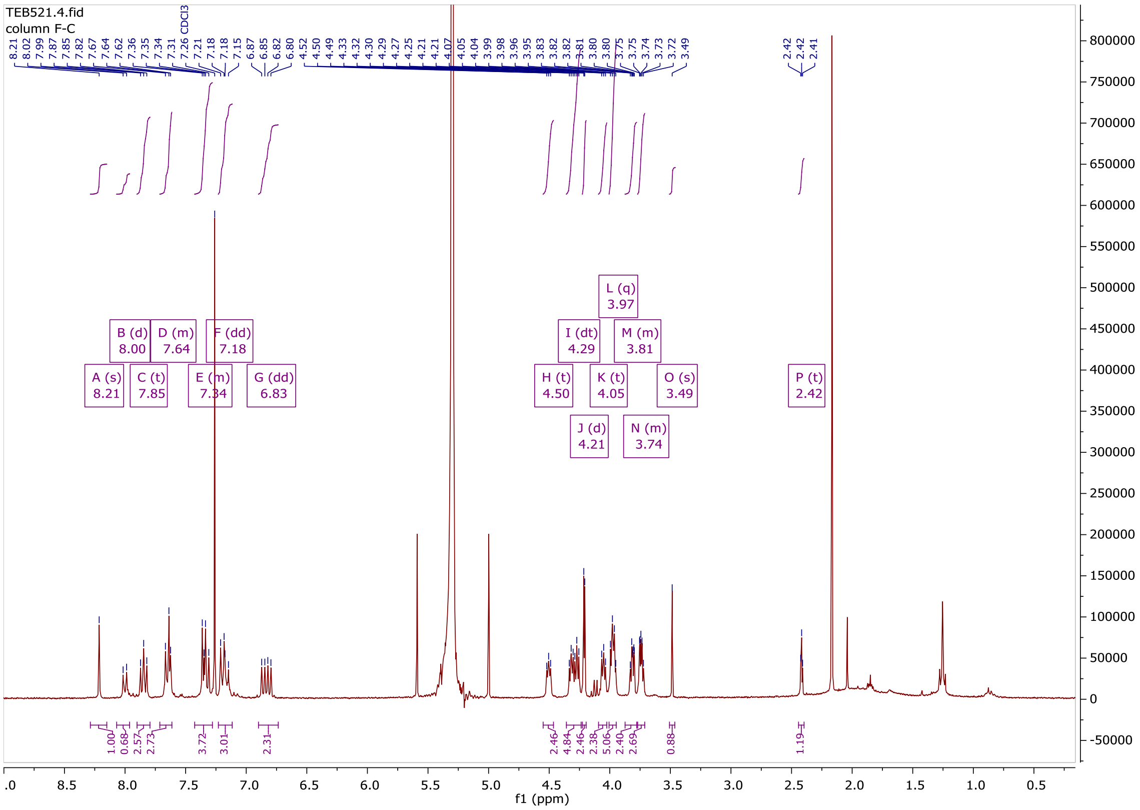

Figure S5. *^{1}H NMR spectrum of intermediate* ***1*** *(300 MHz, 298 K) in CD_2Cl_2.*

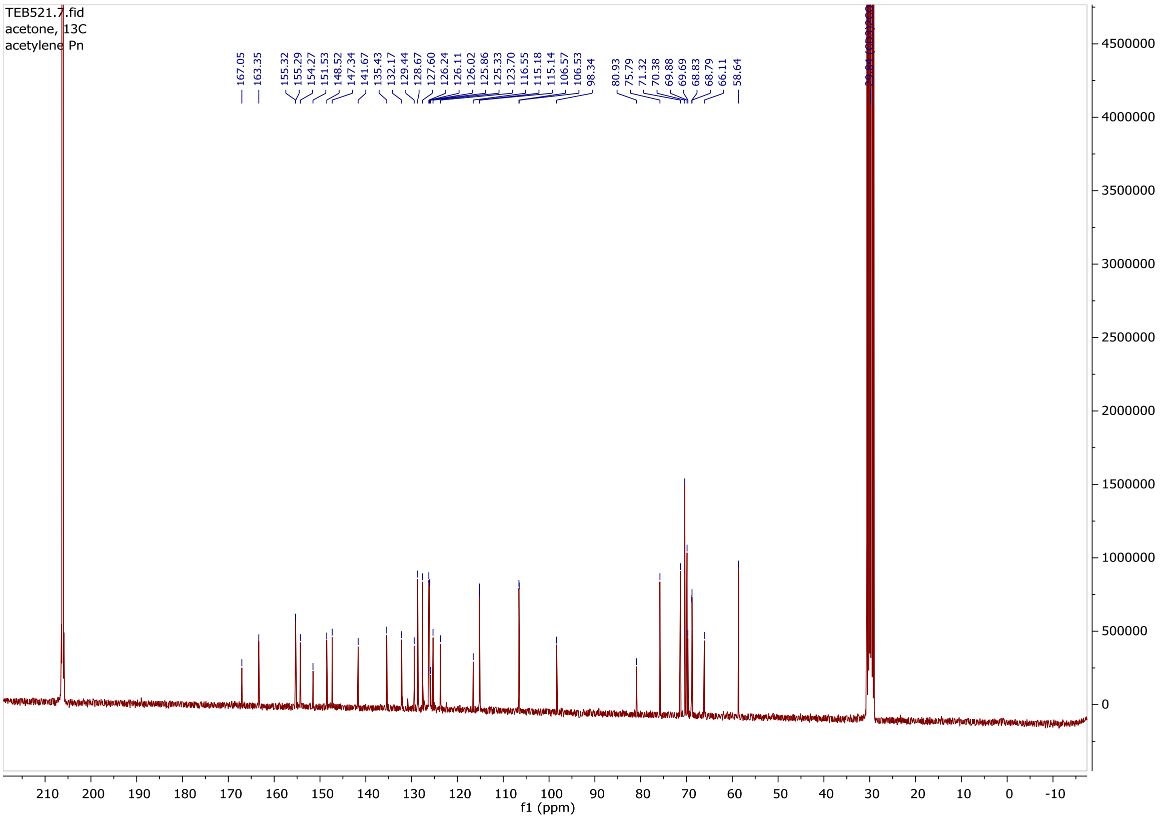

Figure S6. *^{13}C NMR spectrum of intermediate* ***1*** *(300 MHz, 298 K) in CD_2Cl_2.*

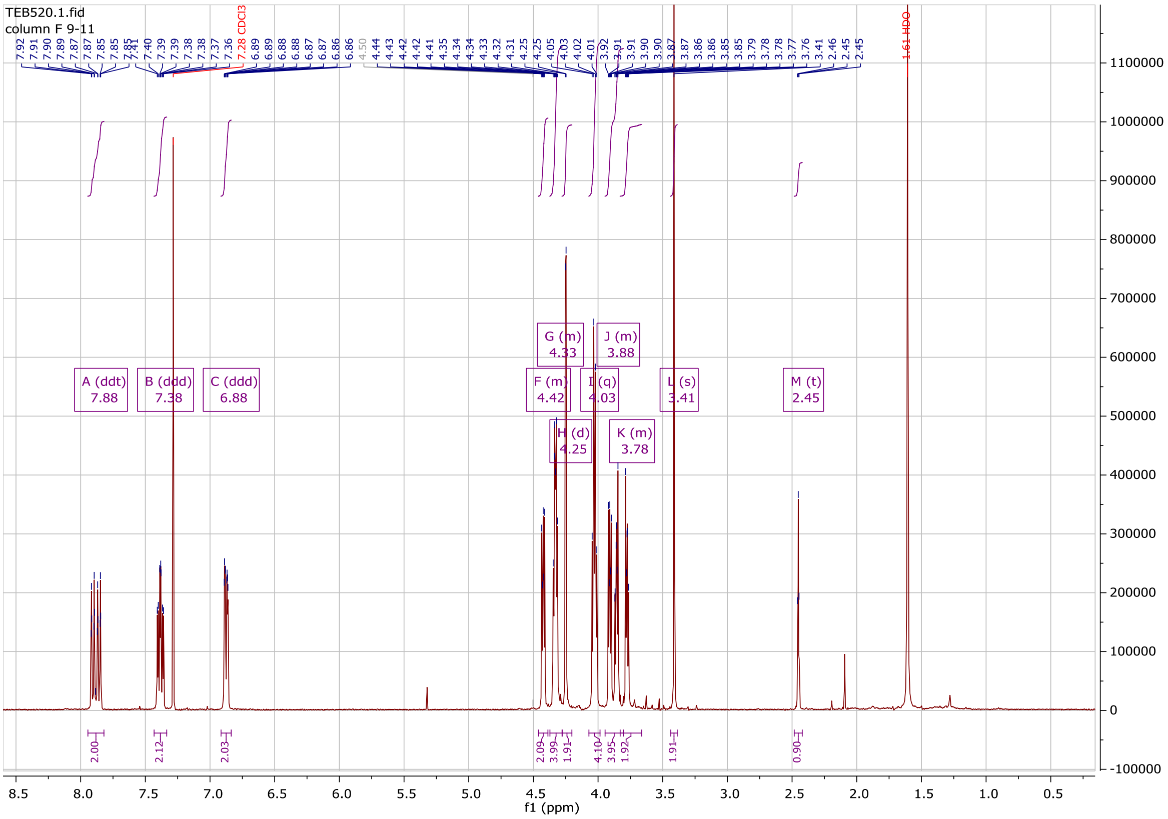

Figure S7. *^{1}H NMR spectrum of intermediate* ***5*** *(400 MHz, 298 K) in $CDCl_3$.*

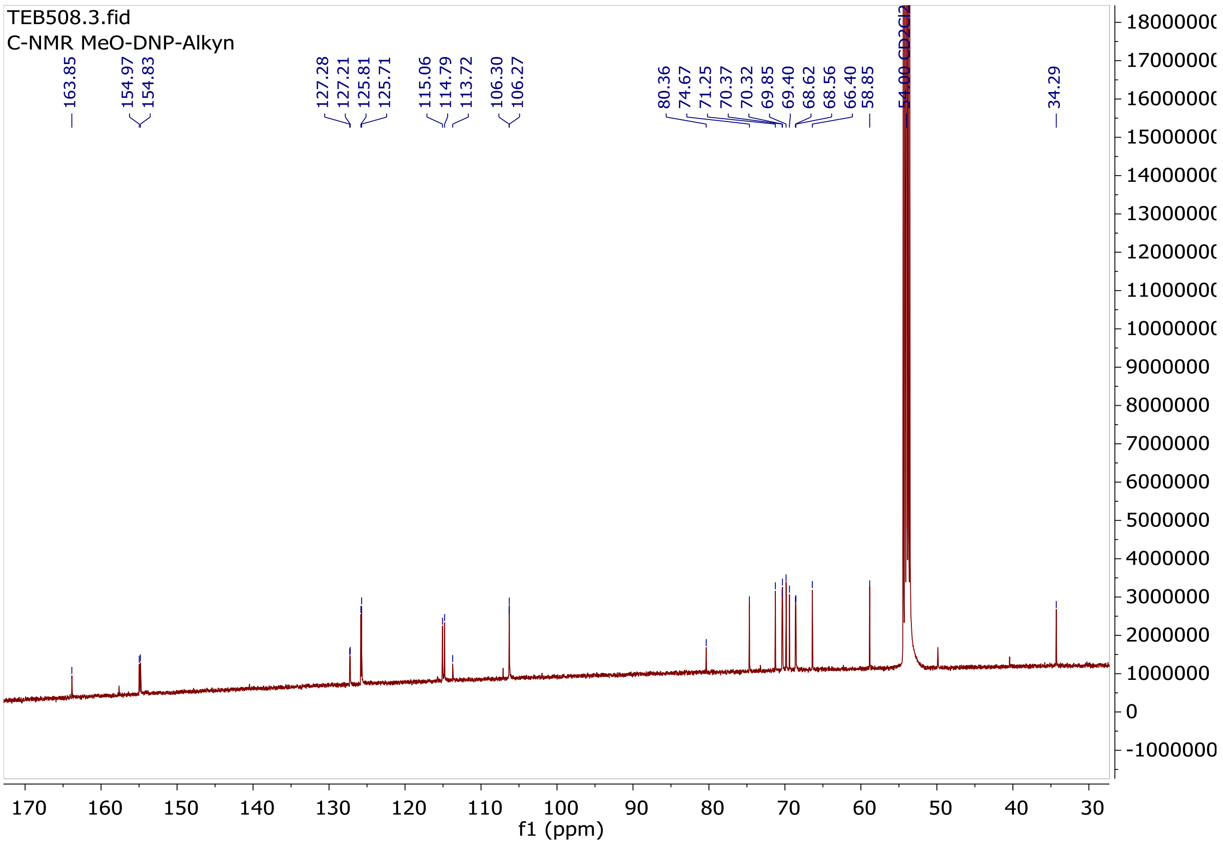

Figure S8. *^{13}C NMR spectrum of intermediate **5** (500 MHz, 298 K) in CD_2Cl_2*

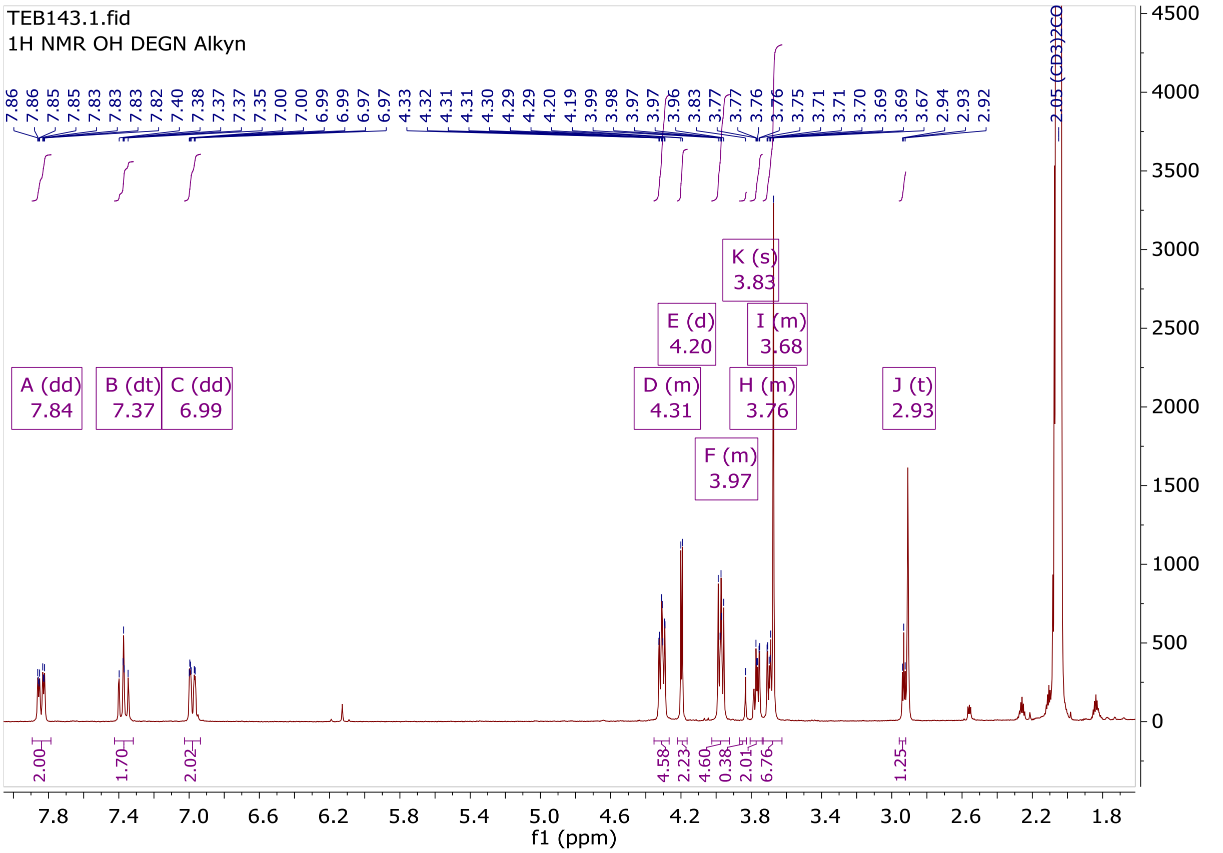

Figure S9. *^{1}H NMR spectrum of intermediate **4** (300 MHz, 298 K) in $(CD_3)_2CO$.*

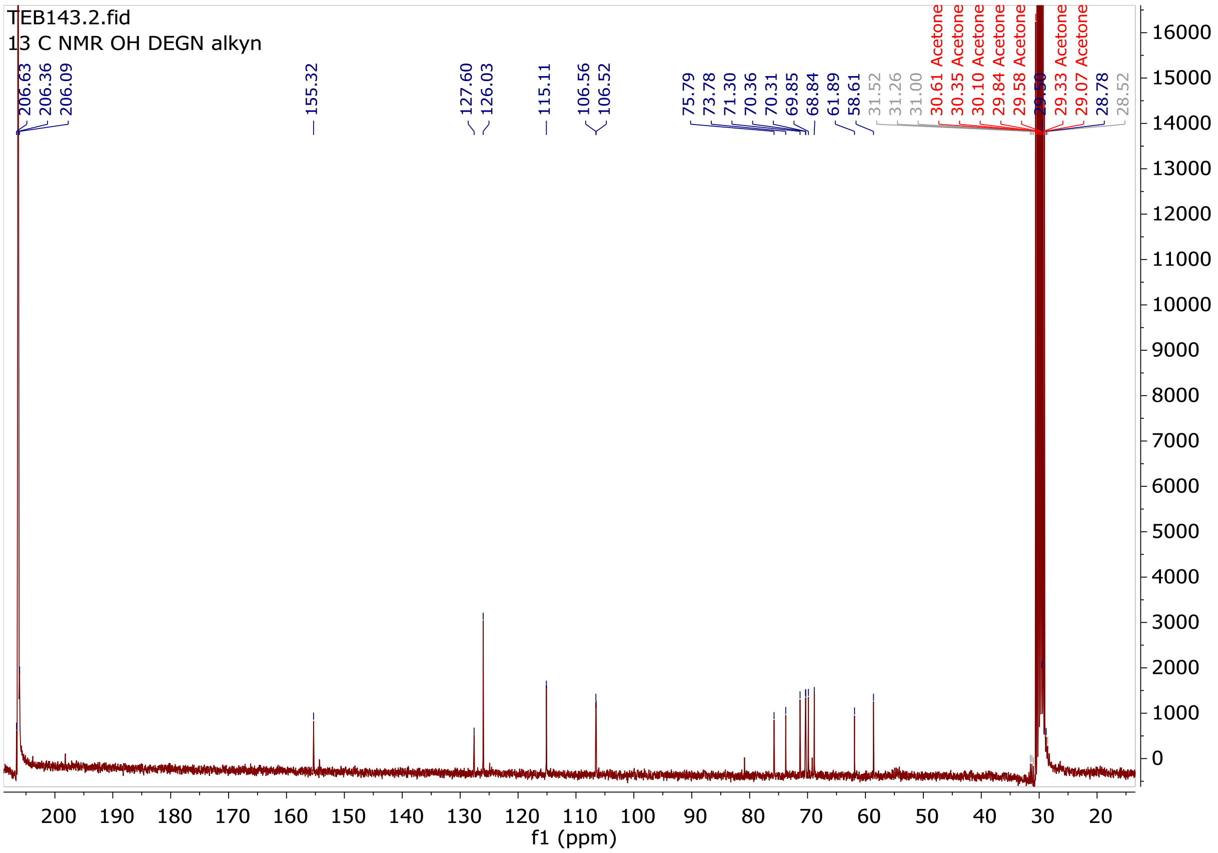

Figure S10. *^{1}H NMR spectrum of intermediate* ***4*** *(300 MHz, 298 K) in $(CD_3)_2CO$.*

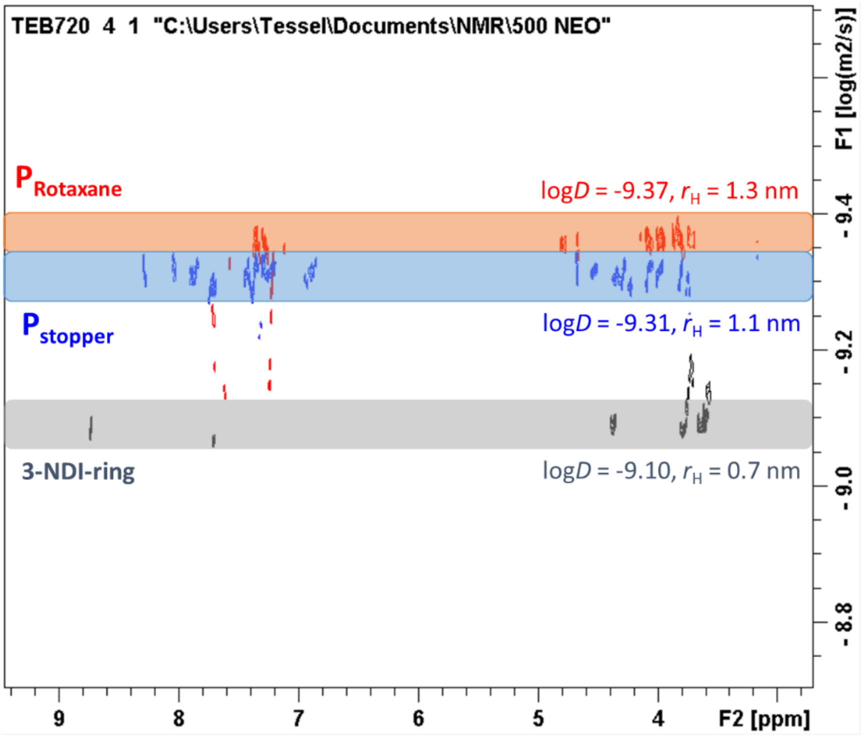

Figure S11. *DOSY ^{1}H NMR spectrum of* $\mathbf{P}_{\mathbf{Station}}$ *(red) and* $\mathbf{P}_{\mathbf{Rotaxane}}$ *(blue) dye (2 mM in CD_2Cl_2).*

S1.4. ESI–HRMS spectra

ElectroSpray Ionization High Resolution Mass Spectrometry (ESI–HRMS)

Mass spectra were collected on a HR–ToF Bruker Daltonik GmbH (Bremen, Germany) Impact II, an ESI–ToF MS capable of resolution of at least 40000 FWHM. Detection was in positive-ion mode and the source voltage was between 4 and 6 kV. The sample was introduced with a syringe pump at a flow rate of 18 µl hr^{-1}. The drying gas (N_2) was held at 180°C. The machine was calibrated prior to every experiment via direct infusion of a TFA-Na solution, which provided a *m/z* range of singly charged peaks up to 3500 Da in both ion modes. Software acquisition Compass 2.0 for Otof series. Software processing Compass DataAnalysis 4.0 sri (x64). Processing of the displayed spectra was further performed using the mMass software, all spectra were averaged and baseline corrected.

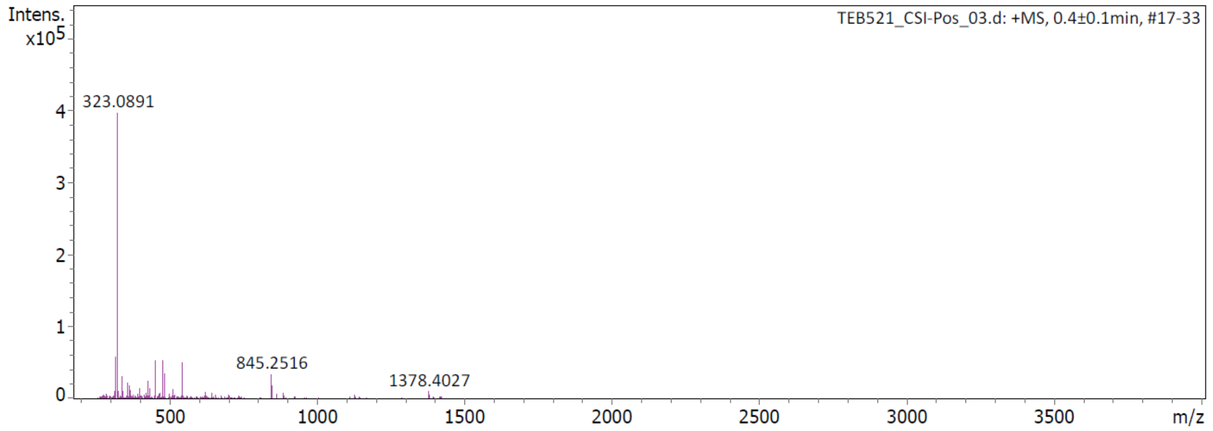

Figure S12. *Full ESI–HRMS for compound* ***1*** *in positive mode. Zoom-ins of m/z~1370–1400 can be found in Figure S13–14.*

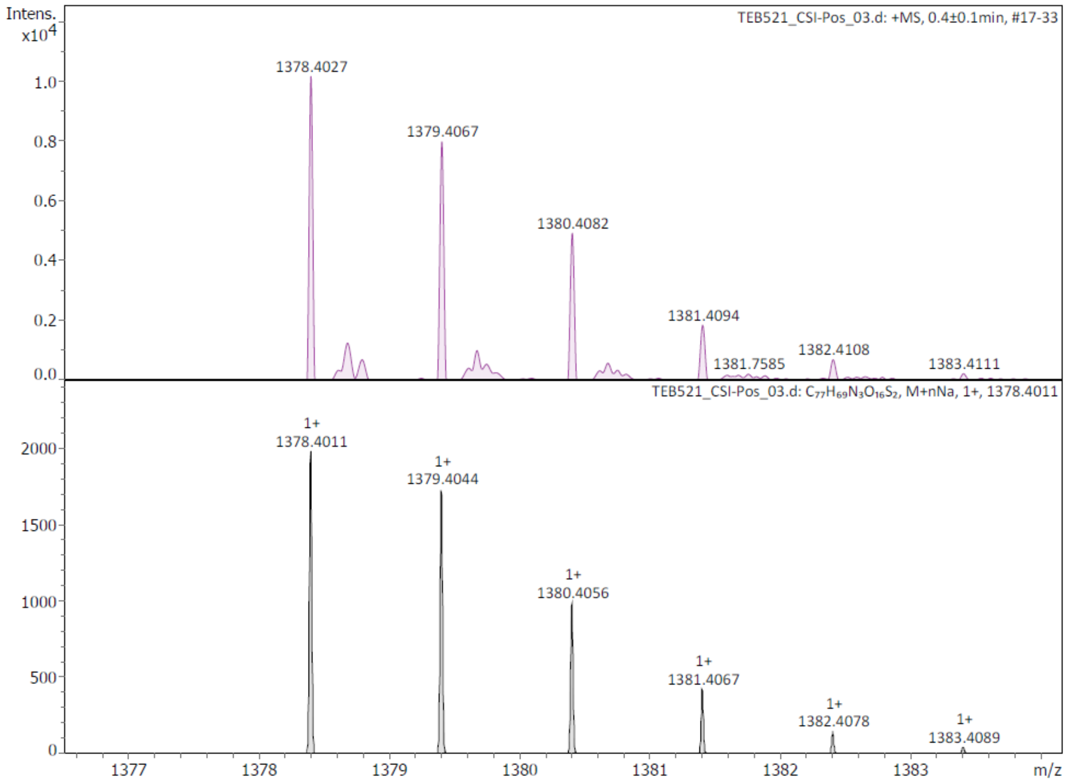

Figure S13. *ESI–HRMS peak found at m/z = 1378.4027 (top, purple) calculated for [(**1**)Na]$^{+}$ 1378.4011 (black, bottom).*

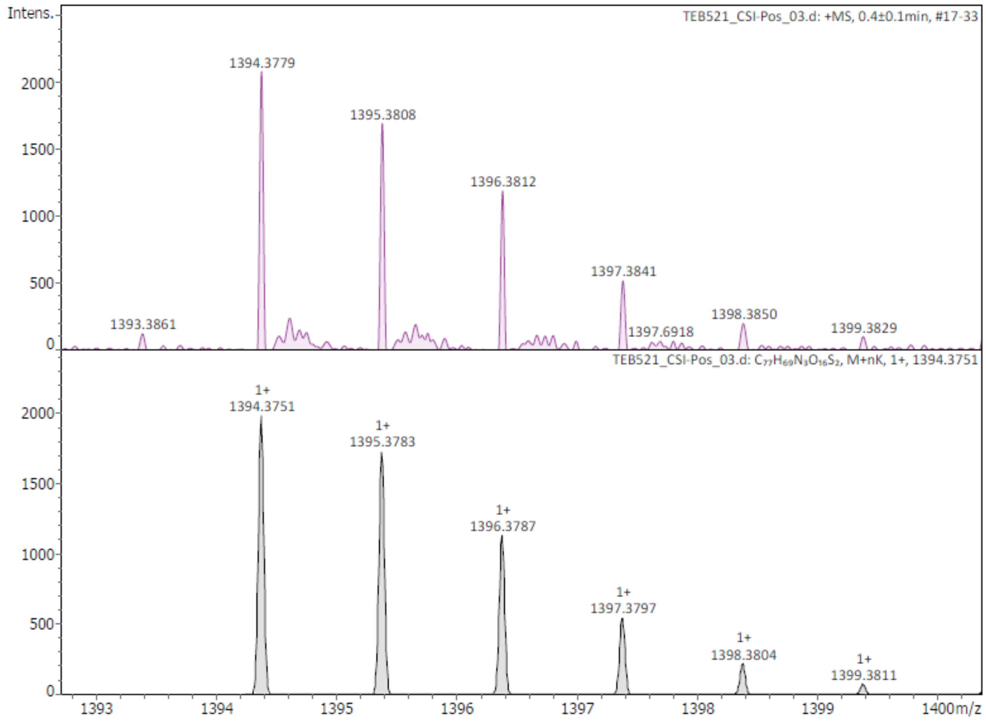

Figure S14. *ESI–HRMS peak found at m/z 1394.3779 (top, purple) calculated for [(**1**)K]$^{+}$ 1394.3751 (bottom, black).*

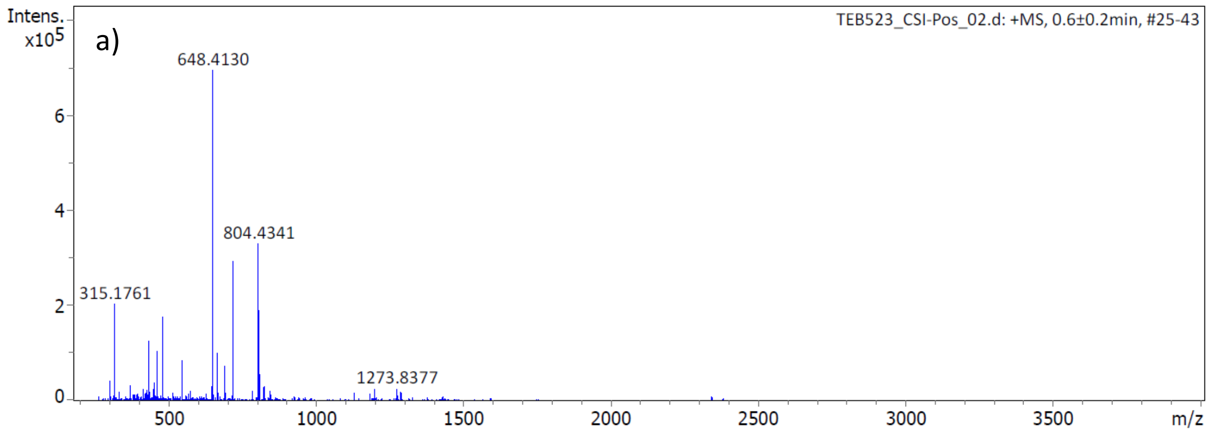

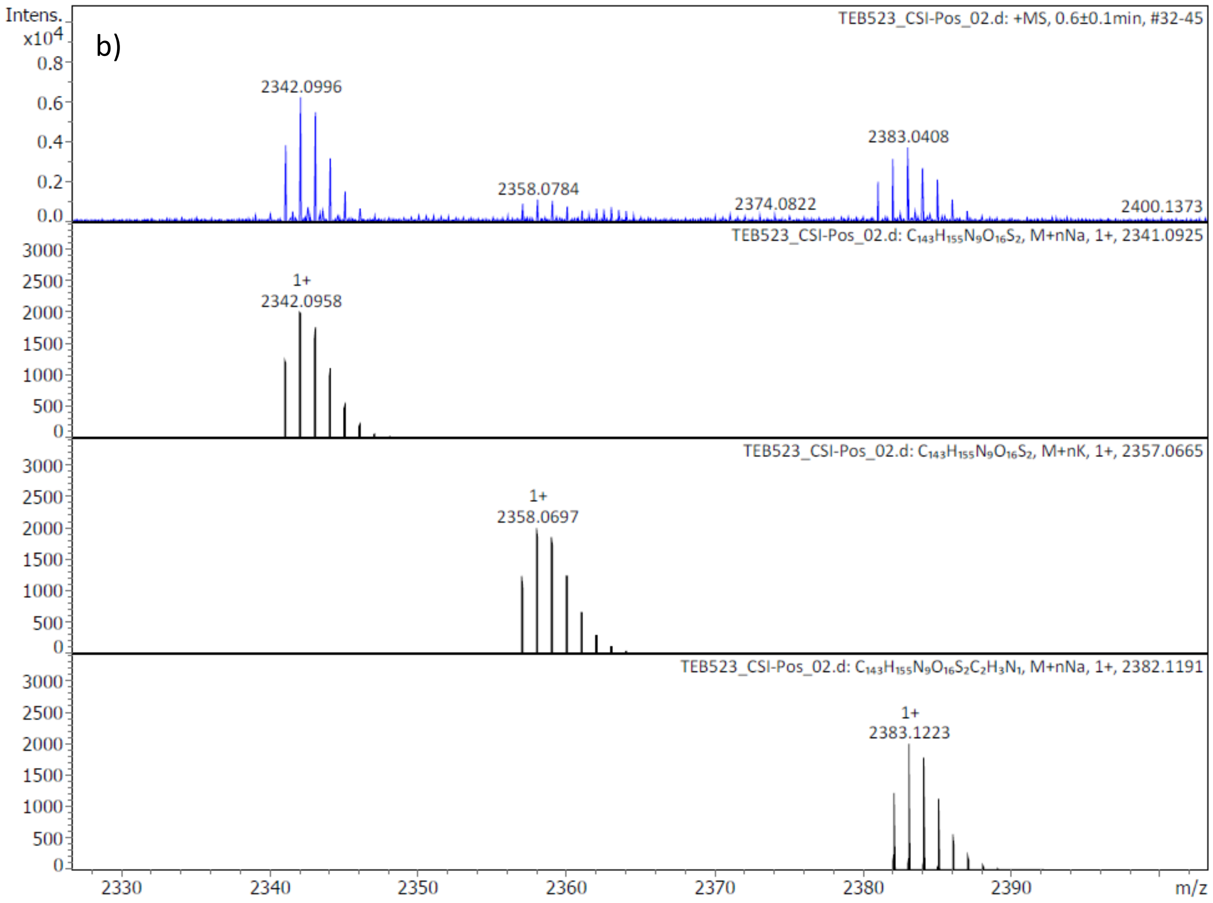

Figure S15. *ESI–HRMS for* ***P****$_{Stopper}$* *dye in positive mode a) Full spectrum b) Zoom (experimental at top, blue) and calculated spectra (black). [(**P**$_{stopper}$)Na]$^+$ calculated 2342.0958; found 2342.0996, [(**P**$_{Stopper}$)K]$^+$ calculated 2358.0697; found 2358.0784, [(**P**$_{stopper}$)CH_3CNNa]$^+$ calculated 2383.1223, found 2383.0408*

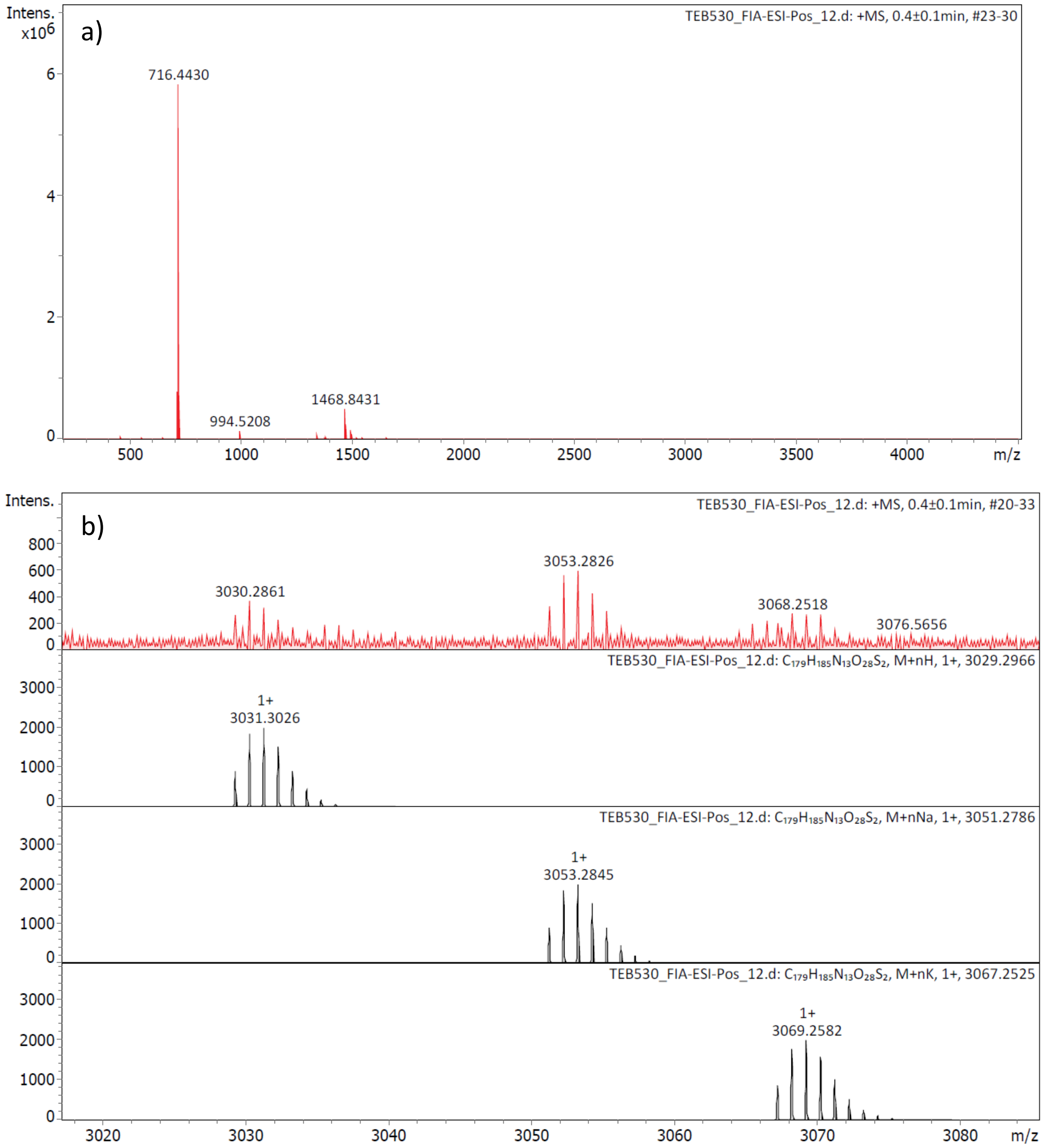

Figure S16. *ESI–HRMS spectrum for* $\mathbf{P}_{\mathbf{Rotaxane}}$ *dye in positive mode a) Full spectrum b) Zoom (experimental, top, red) and calculated spectra (black). [(*$\mathbf{P}_{\mathbf{Rotaxane}}$*)Na]*$^{+}$ *calculated 3053.2845; found 3053.2826, [(*$\mathbf{P}_{\mathbf{Rotaxane}}$*-H]*$^{-}$ *calculated 3029.2869, found 3029.2895, [(*$\mathbf{P}_{\mathbf{Rotaxane}}$*-H)CH*$_2$*Cl*$_2$*]*$^{-}$ *calculated 3065.2625; found 3065.2632.*

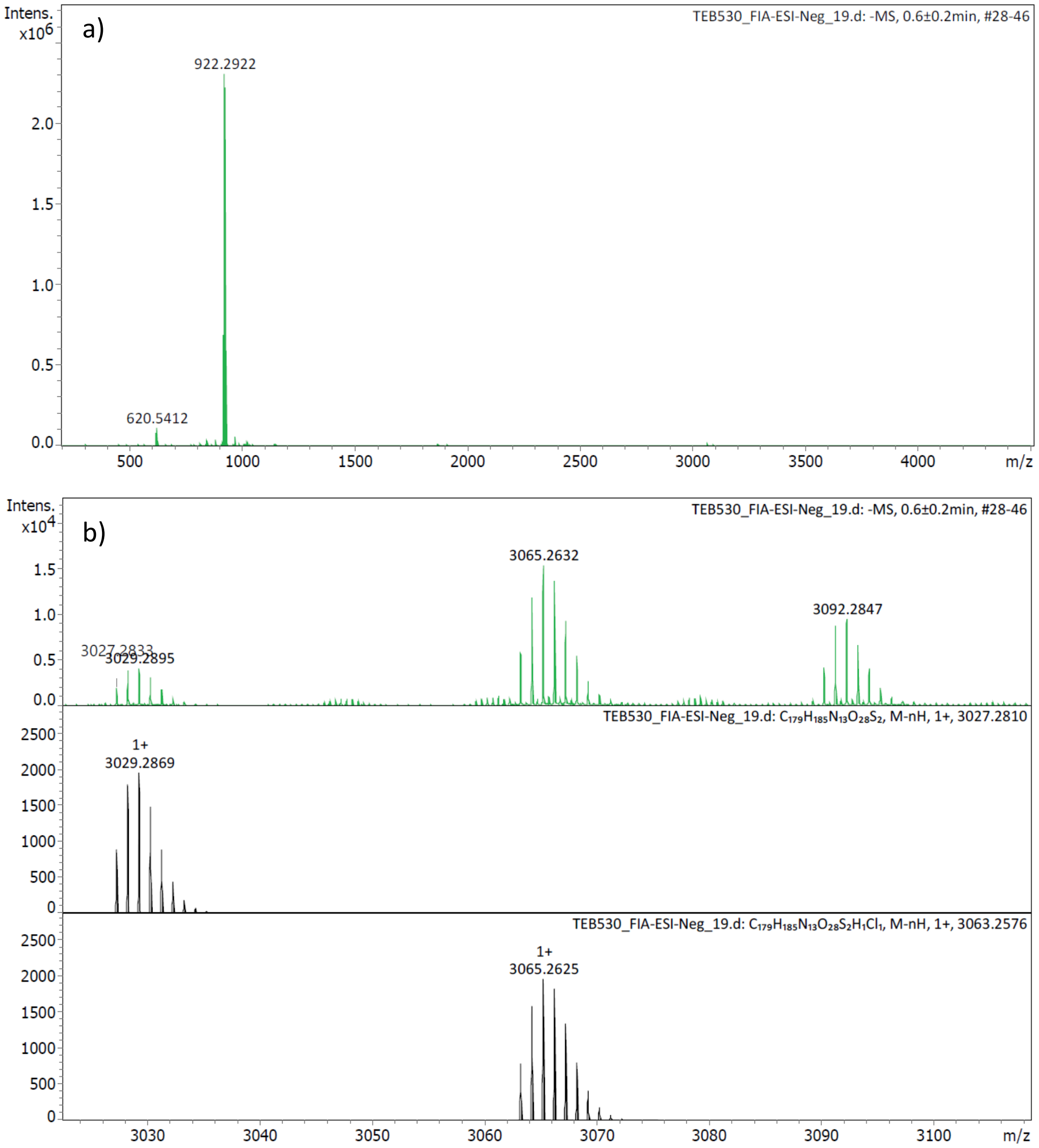

Figure S17. *ESI–HRMS spectrum for* ***P****$_{\textbf{Rotaxane}}$ dye in negative mode a) Full spectrum b) Zoom (experimental, top, green) and calculated spectra (black). [(***P****$_{\textbf{stopper}}$)Na]$^+$ calculated 2342.0958; found 2342.0996, [(***P****$_{\textbf{stopper}}$)K]$^+$ calculated 2358.0697; found 2358.0784, [(***P****$_{\textbf{stopper}}$)CH_3CNNa]$^+$ calculated 2383.1223, found 2383.0408.*

S1.5. Binding studies

Experimental details

$P_{Station}$ (1.4 mg, 0.6 µmol, 1 equiv.) and **3-NDI-ring** (0.9 mg, 1.2 µmol, 2 equiv.) were dissolved in CD_3CN (0.6 mL). ^{1}H NMR and DOSY ^{1}H NMR were recorded. Exact determination of binding of **3-NDI-ring** to the **DNP**-arm within **$P_{Stopper}$** and **$P_{Rotaxane}$** were prohibited by the limited solubility of both the dye and **3-NDI-ring**.

Results Qualitative binding experiment.

Qualitative binding was demonstrated by ^{1}H NMR by comparing the spectra of the 1:2 mixture **$P_{Stopper}$/3-NDI-ring** to that of individual **$P_{Stopper}$** and **3-NDI-ring** solutions. These ^{1}H NMR spectra are given in Figure S18, showing signal broadening and an upfield shift of the **3-NDI-ring** signals for both the naphthalene diimide signal and pyromellitic signal ($\Delta\delta$ = 0.04 ppm). The resonance from the 1,5-dioxonapthalene protons and the thiophene protons of **$P_{Stopper}$** also undergo an upfield shift ($\Delta\delta$ = 0.02 ppm), while the other aromatic protons are unaffected. Unfortunately, the binding constant of the **$P_{Stopper}$/3-NDI-ring** complex could not be determined because of the limited solubility of the dye and **3-NDI-ring**. From these ^{1}H NMR shifts, we estimated that the K_{ass} is 50 M^{-1} by approximation based on the shifts obtained for titrating **DNP-fragment** to **3-NDI-ring** (Figure S19–20).[1] The association strength of **$P_{Rotaxane}$** when only one **DNP**-arm is available was approximated with the **3-NDI-thread** and **DNP-fragment** model compounds. Supramolecular complex formation between **3-NDI-thread** and **DNP-fragment** was measured by ^{1}H NMR titration providing K_{ass} (5 M^{-1}) (Figure S21). Based on these binding approximations we expect that the interaction of **3-NDI-ring** is an order of magnitude stronger for **$P_{Stopper}$** (approximately 50 M^{-1}) in comparison to **$P_{Rotaxane}$** (approximately 5 M^{-1}). This difference in binding strength presumably arises from the cooperative binding of two **DNP**-arms in **$P_{Stopper}$** instead of one **DNP**-arm in **$P_{Rotaxane}$**.

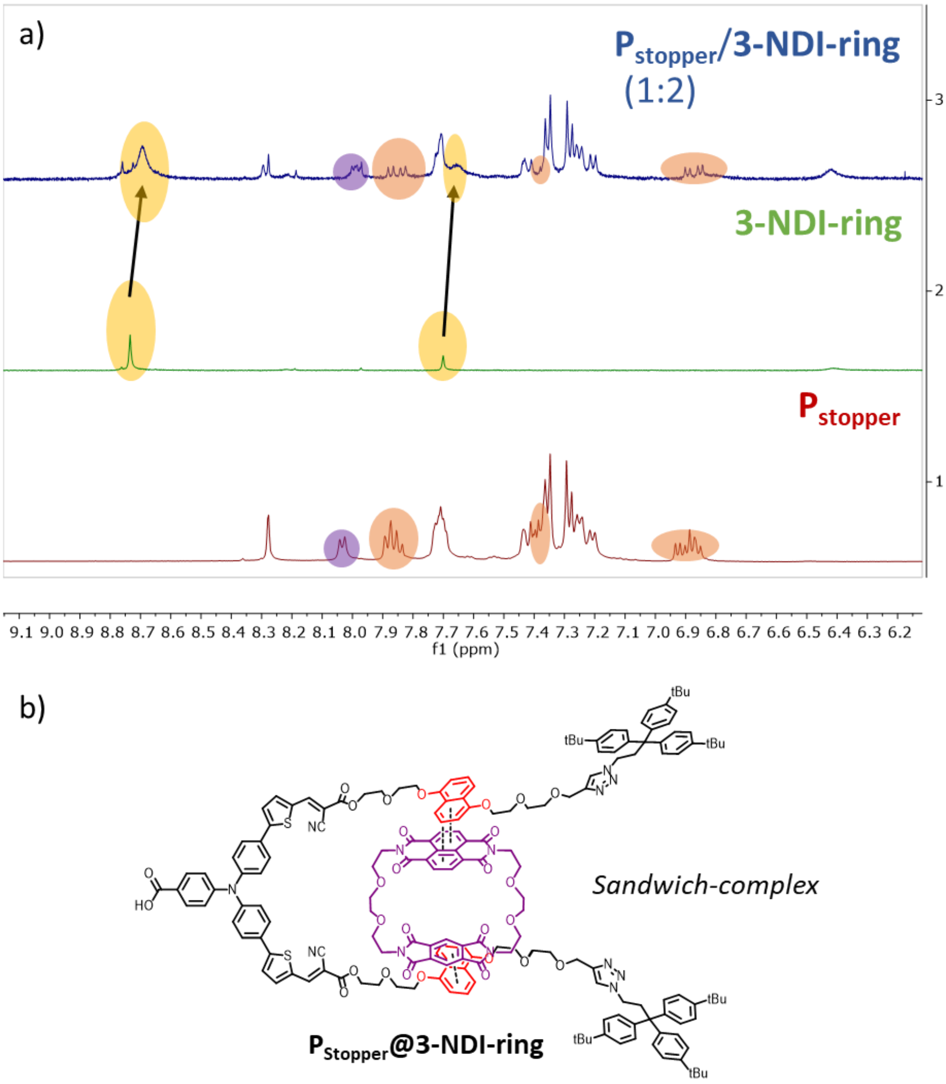

Figure S18. *a)* ^{1}*H NMR spectra of* **$P_{Stopper}$, 3-NDI-ring** *and a sample containing both* **$P_{Stopper}$/3-NDI-ring** *1:2 showing the broadening and upfield shift of the* **3-NDI-ring** *signals for both the naphthalene diimide signal and pyromellitic signal ($\Delta\delta$ = 0.04 ppm, yellow). The signals of the 1,5-dioxonapthalene protons (red) and the thiophene protons (purple) of* **$P_{Stopper}$** *also undergo an upfield shift ($\Delta\delta$ = 0.02 ppm). b) Proposed sandwich-complex between* **3-NDI-ring** *and* **$P_{Stopper}$**.

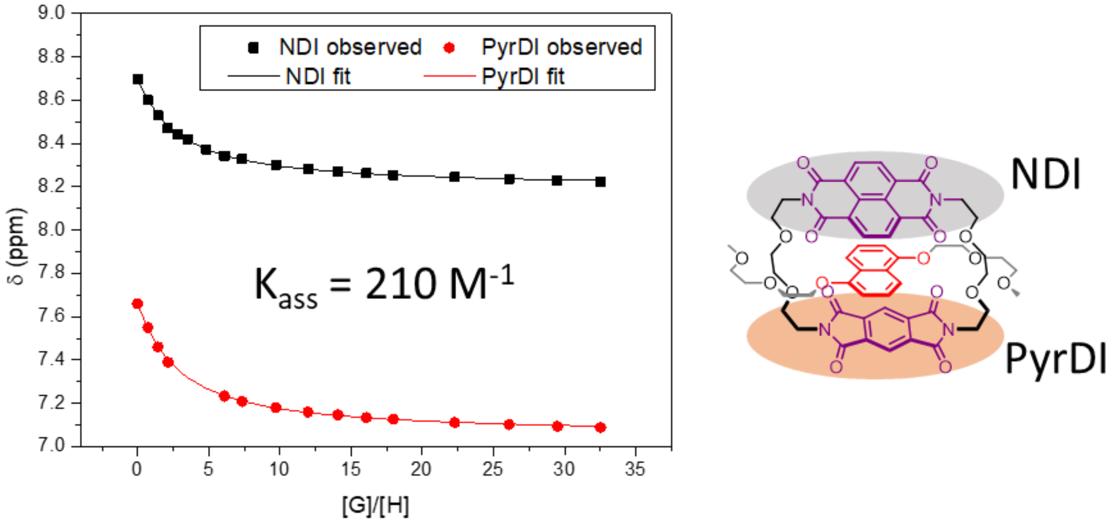

Figure S19. *The titration based on Figure S20 was used to determine the association constant K_{ass}.*[1]

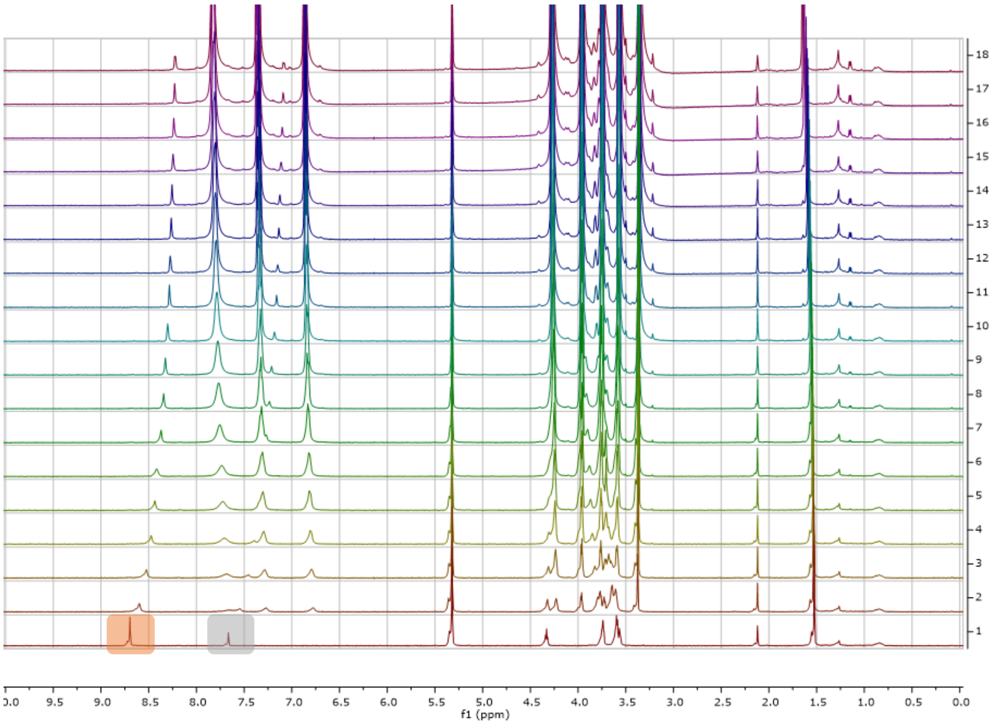

Figure S20. *^{1}H NMR spectrum of* ***3-NDI-ring*** *(2 mM) titrated with increasing amounts of* ***DNP-fragment*** *(175 mM) (500 MHz, 298 K) in CD_2Cl_2. Both aromatic signals of the* ***3-NDI-ring*** *at 8.68 ppm (Naphthalic proton, purple) and 7.65 ppm (phenyl proton, yellow) show an upfield shift upon pseudorotaxane formation.*[1]

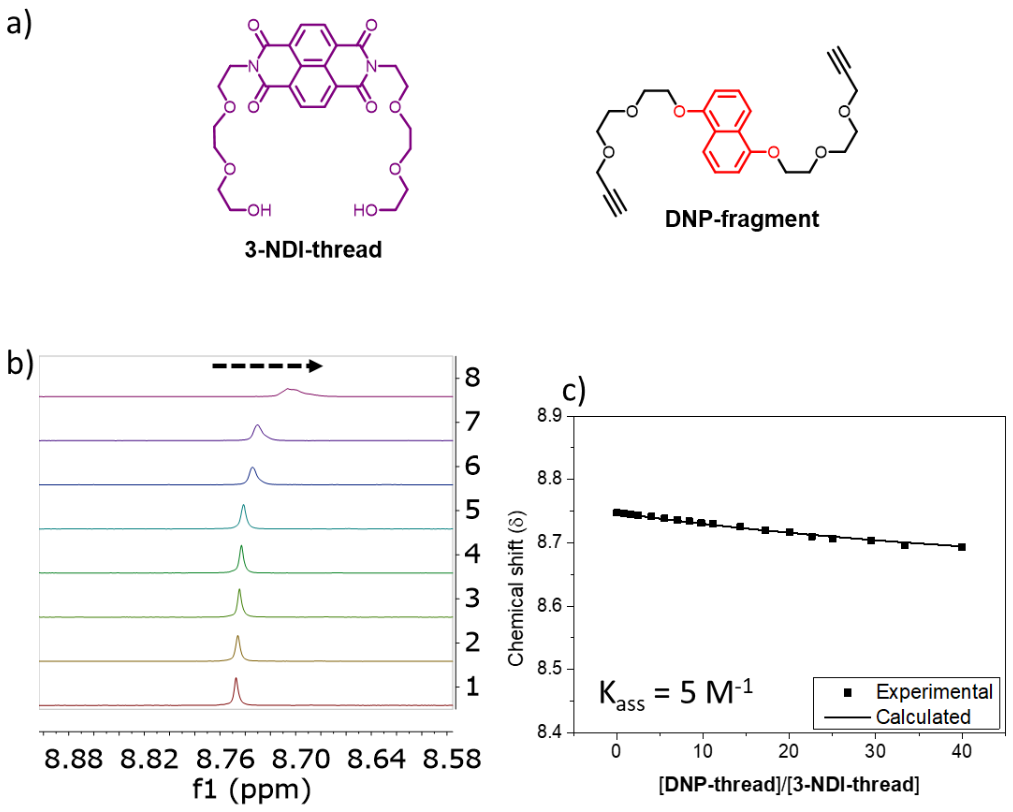

Figure S21. *a) Schematic representation of interaction between **3-NDI-thread** as redox mediator and the **P$_{Stopper}$** dye and **NDP-thread**. b) Binding study of **3-NDI-thread** and **DNP-fragment**. ^{1}H NMR spectrum of **3-NDI-thread** (2 mM) titrated with increasing amounts of **DNP-fragment** (200 mM) (300 MHz, 298 K) in CD_2Cl_2. c) The aromatic signal of the **3-NDI-thread** at 8.75 ppm show an upfield shift upon complex formation. The binding data was fitted with a Matlab script.*[8]

S1.6. Dye adsorption onto NiO

This procedure was performed according to a previous report.[1]

Method for Determination of dye loading

The dye loading of the freshly prepared NiO working electrodes was determined by performing a dye uptake experiment. A solution of dye was prepared (~0.15 mM in MeCN) and the UV–Vis spectrum was recorded in a 1 cm path length cuvette. The freshly prepared NiO working electrode was added to this cuvette. Importantly, this working electrode was cut (~0.7×0.7 cm) such that complete insertion into the cuvette was possible. The cuvette was capped to prevent evaporation of MeCN. After 24 hours the UV–Vis spectrum was measured to determine the decrease in absorbance of the dye solution. This decrease in absorbance translates into how much dye has been adsorbed onto the NiO.

The raw data was baseline corrected and the difference in absorbance was determined from this data at λ_{max}. The amount of adsorbed dye on NiO (*n*, mol) was calculated using Equation 1, where the difference in absorbance (ΔA) is divided by the extinction coefficient (ε, M^{-1} cm^{-1}) the volume of dye solution in the cuvette (*V*, L) and path length of the cuvette (*l* in cm, typically 1 cm).

$$n = \frac{\Delta A}{\varepsilon\, l\, V} \qquad (1)$$

Equation 2 was used to determine the dye loading (Γ in mol cm^{-2}), where the amount of adsorbed dye (*n*, mol) is divided by the surface area of NiO (*S*, cm^2), determined with BET measurements described in Supplementary Information Section 2.8. The unsensitized photocathodes are comprised of 0.72 mg NiO, with the surface area per NiO working electrode determined to be 4.26×10^{-2} cm^2.

$$\Gamma = \frac{n}{S} \qquad (2)$$

The porosity of the NiO nanoparticles was estimated by nitrogen absorption via Brunauer–Emmett–Teller (BET) analysis on a Thermo Scientific Surfer Analyzer. The NiO paste was applied and sintered according to the method described in Supplementary Table 3. The surface area NiO nanoparticles under study was determined to be 59.1 cm^2 g^{-1} calculated from the adsorption isotherm.[1] The spots comprise of 0.72 mg NiO and the surface area per NiO working electrode was determined to be 4.26×10^{-2} cm^2.

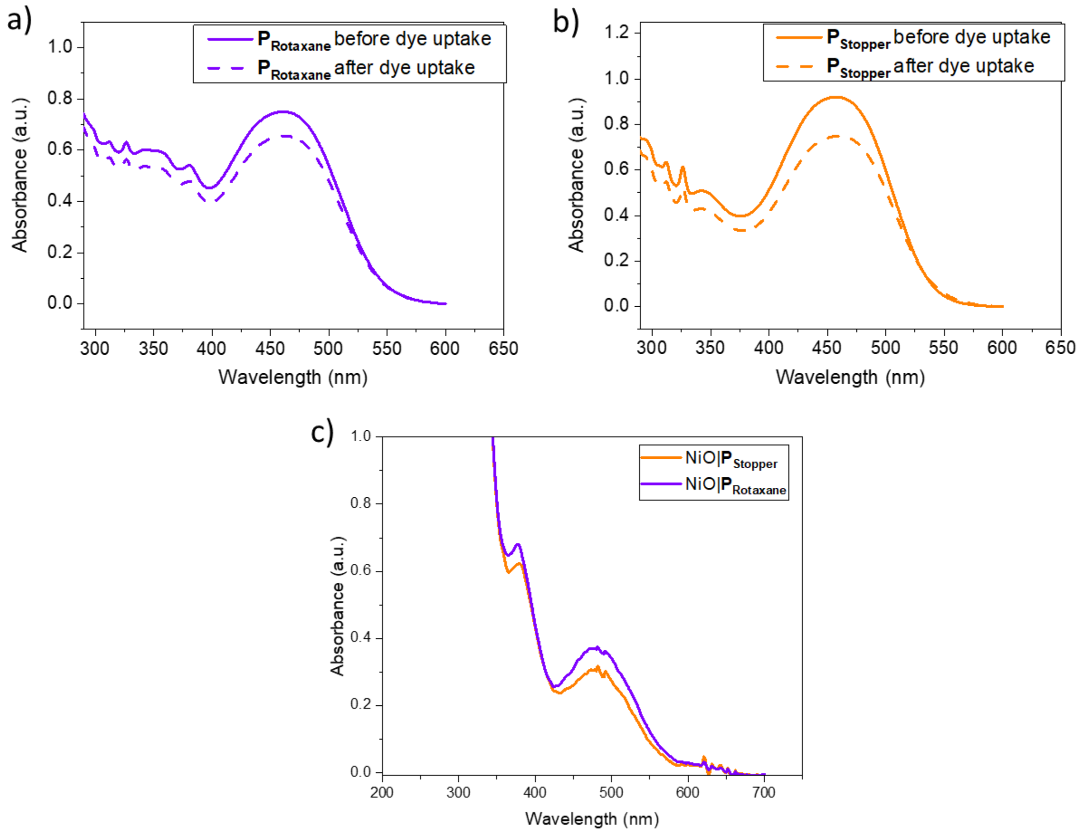

Figure S22. *Dye-uptake experiments. The spots comprise of 0.72 mg NiO and the surface area per NiO working electrode was determined to be 4.26×10^{-2} cm^2 via BET analysis.[1] a)* $\mathbf{P_{Rotaxane}}$ *Dye loading experiment showing the decrease in* $\mathbf{P_{Rotaxane}}$ *after 16 h starting at t=0, 20 µM, 1.5 mL. This difference in absorption corresponds to 1.07×10^{-7} mol cm^{-2}* $\mathbf{P_{Rotaxane}}$ *on NiO. b)* $\mathbf{P_{Stopper}}$ *Dye loading experiment showing the decrease in* $\mathbf{P_{Stopper}}$ *after 16 h starting at t=0, 20 µM, 1.5 mL. This difference in absorption corresponds to 1.58×10^{-7} mol cm^{-2}* $\mathbf{P_{Stopper}}$ *on NiO. c) UV–Vis absorption spectra of thin films of* $\mathbf{P_{Rotaxane}}$ *and* $\mathbf{P_{Stopper}}$ *on NiO.*

S1.7 Differential Pulse Voltammogram $P_{Rotaxane}$ + $P_{Stopper}$

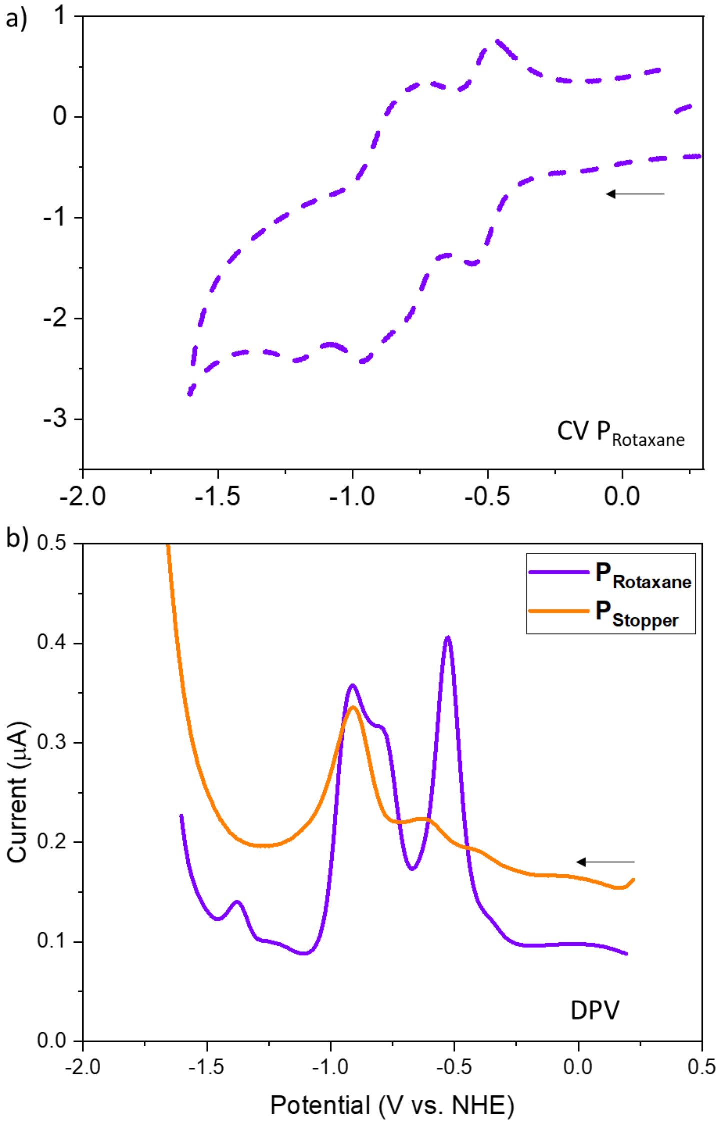

Figure S23. *a) Cyclic voltammogram and b) Differential pulse voltammogram (DPV) of* $\mathbf{P}_{Rotaxane}$ *and* $\mathbf{P}_{Stopper}$ *(0.5 mM) recorded in 0.1 M TBAPF*$_6$ *in DCM with 5 mV s*$^{-1}$ *scan rate starting from +0.2 V to -1.6 V vs. NHE using glassy carbon as working electrode, leakless Ag/AgCl as reference electrode and Pt wire as counter electrode together with cyclic voltammograms of* $\mathbf{P}_{Rotaxane}$ *(dashed line).*

S1.8 Spectroelectrochemistry

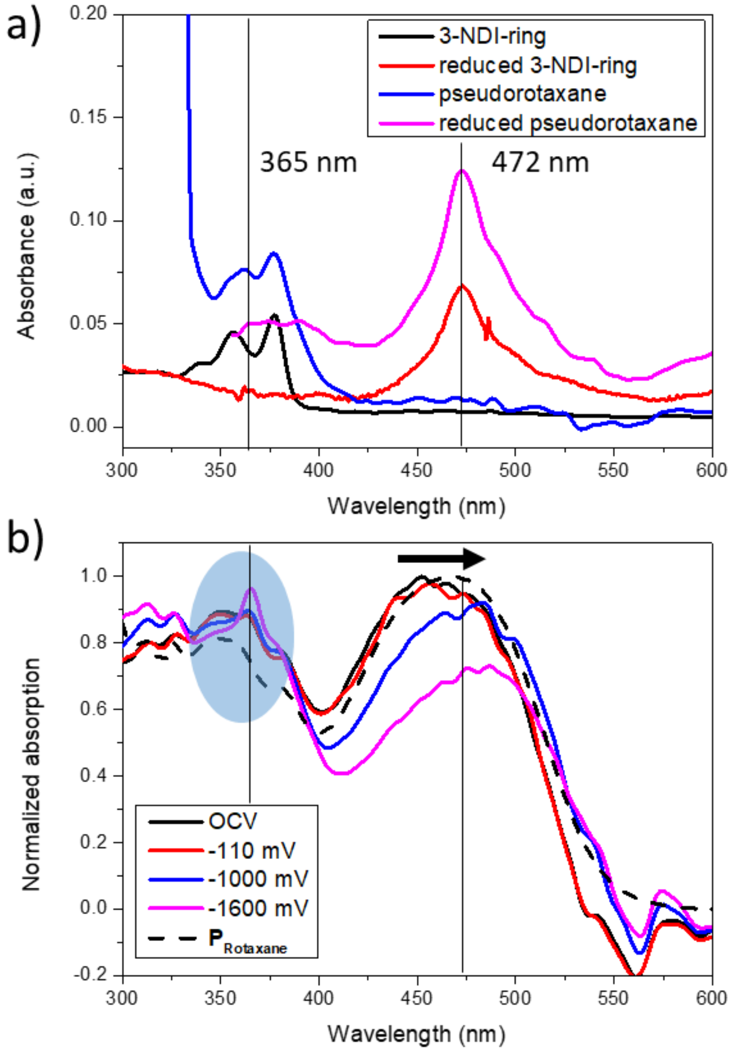

Figure S24. *Spectroelectrochemistry recorded in an OTTLE cell with a Pt grid working electrode, Pt counter electrode and a Ag wire reference electrode, coupled to an Autolab PGSTAT302N potentiostat. a)* ***3-NDI-ring*** *(1 mM) in TBAPF$_6$ (100 mM in MeCN) and the* ***DNP-thread⊂3-NDI-ring*** *(0.5 mM) pseudorotaxane. b)* ***P$_{Rotaxane}$*** *(0.2 mM) in TBAPF$_6$ (100 mM in MeCN).*

S2. Ultrafast spectroscopy experiments

S2.1. Experimental details

Experimental set-up fs-TA measurements

Femtosecond (fs) time-resolved measurements were done by means of transient absorption (TA) spectroscopy. A detailed description of the experimental setup can be found in work by Huijser and co-workers.[9] Briefly, the fs TA experiments were performed on thin-film substrates in an electrolyte (1.5 mL, 1 M LiTFSI valeronitrile/MeCN, v/v, 15:85) in absence and in presence of the **3-NDI-ring** (5.9 mM), housed in a quartz cuvette (Hellma QS, 10.00 mm path length). A Ti:Sapphire amplifier (Coherent Legend Elite) was used to generate ca. 800 nm pulses (@5.0 kHz repetition rate) with a pulse duration of 38 ± 1 fs (FWHM). The 800 nm output was split into two beams (85:15) via a beam splitter. The 480 nm pump beam was generated by directing the main component of the 800 nm beam into an optical parametric amplifier (Coherent Opera). The white-light continuum-probe was generated by guiding a further attenuated fraction of the 800 nm beam through a mechanical delay stage prior to focusing into a CaF_2 crystal (Newlight Photonics, 3 mm thick, continuously moved to avoid thermal damage). The samples were mounted on a translational stage continuously moving with a velocity of ca. 1 mm s^{-1}, thereby refreshing the measurement area regularly to avoid any charge accumulation and photodegradation phenomena. The data were corrected for chirp using Matlab and analyzed using the open-source program Glotaran.[10]

Experimental set-up time-resolved fluorescence

The time-resolved fluorescence was measured by a streak camera setup (Hamamatsu, C10910), using the attenuated output of a Fianium laser (FP-532-1-s, center wavelength 532 nm, pulse duration of 300 fs, 80.37 MHz repetition rate). The laser beam was focused using a quartz lens with 50 mm focal length onto the samples in a quartz cuvette (Hellma, 10 mm optical path length). The fluorescence was collected using two 2-inch diameter 50 mm focal length glass lenses and focused on the input of a spectrograph (Acton SP2300, Princeton Instruments, slit width set at 100 mm) using a grating with 50 lines/mm blazed at 600 nm. The output of the spectrograph was sent to the photocathode of the streak camera. Before each series of experiments, the spectral calibration was checked using a Hg/Ar calibration lamp (Oriel,

LSP035) and adapted if necessary. The measured fluorescence spectra were corrected for the spectral sensitivity of the setup by comparison of the measured and provided spectrum of a black body calibration lamp (Ocean Optics, HL-2000).The spectral calibration was checked and adapted if necessary using a Hg/Ar calibration lamp (Oriel, LSP035).The spectral sensitivity of the fluorescence spectra was corrected by the Equation 3 and 4 below, which was determined by measuring the spectrum of a black body lamp (Ocean Optics, HL-2000) with a calibrated spectrum:

$$Real\ fluoresence\ spectrum = \frac{measured\ fluorescence\ spectrum}{Y} \quad (3)$$

$$Y = 110 - 0.0012\ (wavelength\ in\ nm - 600)^2 \quad (4)$$

Preparation of ZrO_2

This preparation was copied from a former procedure.[1]

FTO (25 × 50 × 2.3 mm) was covered with two pieces of Scotch tape (50 mm) leaving an exposed area of 50 × 10 mm. The ZrO_2 paste was applied onto the FTO via doctor blading. The samples were annealed at 500°C for 30 minutes with a heating ramp of 15°C min^{-1}. The ZrO_2 samples were removed from the oven directly after 30 minutes and placed on a heating plate of 160°C for 10 minutes. The samples were removed from the heating plate to cool to room temperature and cut into 7–9 mm × 25 mm electrodes. The ZrO_2 films were photosensitized in **$P_{Stopper}$** (0.3 mM in MeCN) **$P_{Rotaxane}$** (0.3 mM in MeCN) or **P1** solution (0.3 mM in MeCN) for 16 hours and washed with MeCN.

Preparation of the NiO

The FTO (fluorine-doped tin oxide, Sigma-Aldrich) was cleaned by sonication for 30 minutes in acetone, isopropanol, and ethanol. The precursor solution for NiO consisted of $Ni(NO_3)_2$·6 H_2O (0.3 M) (99.999%, Sigma-Aldrich) urea (0.16 M) (Sigma-Aldrich, >99%) and ethanolamine (0.6 M) (Sigma-Aldrich, >99%) by sequentially dissolving these components in Milli-Q water. Cleaned fluorine-doped tin oxide (FTO) substrates were submerged into the precursor solution for NiO, and films were grown on the FTO for 3 hours at 90 °C. The plates were rinsed with water.

The film was finally calcined at 450 °C in air for 60 min. The as-prepared NiO film was sensitized in the subsequent dye solution in MeCN (P1) (~16 h) and rinsed with MeCN. These plates were dipped in dye solution **P1** (0.3 mM in MeCN), $\mathbf{P_{Stopper}}$ or $\mathbf{P_{Rotaxane}}$ (0.1 mM in MeCN). After 16 hours the plates are taken out of the solution and rinsed with MeCN to remove non-adsorbed dye. The plates changed color red for **P1** and orange for $\mathbf{P_{Stopper}}$ or $\mathbf{P_{Rotaxane}}$.

S2.2. Ultrafast spectroscopy results and discussion

- ***$P_{Rotaxane}$ and $P_{Stopper}$ in solution***

In order to understand the behavior of $\mathbf{P_{Rotaxane}}$ and $\mathbf{P_{Stopper}}$ on the NiO semiconductor, we first study the dyes in solution with fs-TA. In case of $\mathbf{P_{Stopper}}$, we expected similar behavior to the **P1** dye. The **P1** dye has been studied thoroughly in literature,[9,11,12] and we know that oxidation of the **P1** dye in solution leads to the triphenylamine cation, which has a strong absorption around 625 nm.[13] Upon excitation of **P1** in solution, a ground state bleach is observed with a concomitant excited state absorption by **P1***. Due to internal conversion (charge transfer), a red shift is obtained where charge density is transferred from the triphenylamine moiety to the thiophene- and dicyanovinyl groups. The cationic triphenylamine (TPA^+) can contribute to the TA spectra having an absorption at 625 nm.[9,12]

The TA spectra of $\mathbf{P_{Rotaxane}}$ and $\mathbf{P_{Stopper}}$ in solution (1 M LiTFSI valeronitrile/MeCN, 15:85) are represented in Figure S25. The corresponding time constants can be found in Table S1. After excitation of $\mathbf{P_{Stopper}}$ with 480 nm, the TA spectra (Figure S25a) reveal a ground state bleach partly overlapping with the excited state absorption of $\mathbf{P_{Stopper}}$***,** with a concomitant red shift to $\lambda \approx 620$ nm, likely due to internal conversion. Furthermore, some stimulated emission is observed at early times around 615 nm.

In case of $\mathbf{P_{Rotaxane}}$, the TA spectra demonstrate a slightly different feature (Figure S25c). After excitation of $\mathbf{P_{Rotaxane}}$ with 480 nm, the TA spectra (Figure S25a) reveal a ground state bleach partly overlapping with the excited state absorption of $\mathbf{P_{Rotaxane}}$*****. After 1 ps we can see a sharp feature emerging at 620 nm, which is a lot more intense than the red shift observed for $\mathbf{P_{Stopper}}$. At this wavelength (620 nm) the **3-NDI-ring**$^{\bullet-}$ absorbs as is demonstrated by the spectroelectrochemical measurements (Figure 5c). This could indicate that the internal conversion taken place in $\mathbf{P_{Rotaxane}}$***** also involves electron transfer from the dye part to the interlocked **3-NDI-ring**¸ leading to a TPA^+ unit and an interlocked **3-NDI-ring**$^{\bullet-}$ species. This

observation is reflected by comparing the ΔA of the feature emerging at 620 nm for the **P$_{Rotaxane}$** compared to **P$_{Stopper}$**. We can see that in case of **P$_{Stopper}$** both features at 575 nm and 620 nm have the same ΔA, while in case of **P$_{Rotaxane}$** the feature at 620 nm is more intense (Figure S25e,f). The increased ΔA of the feature at 620 nm could imply the emergence of an interlocked **3-NDI-ring$^{•-}$** species.

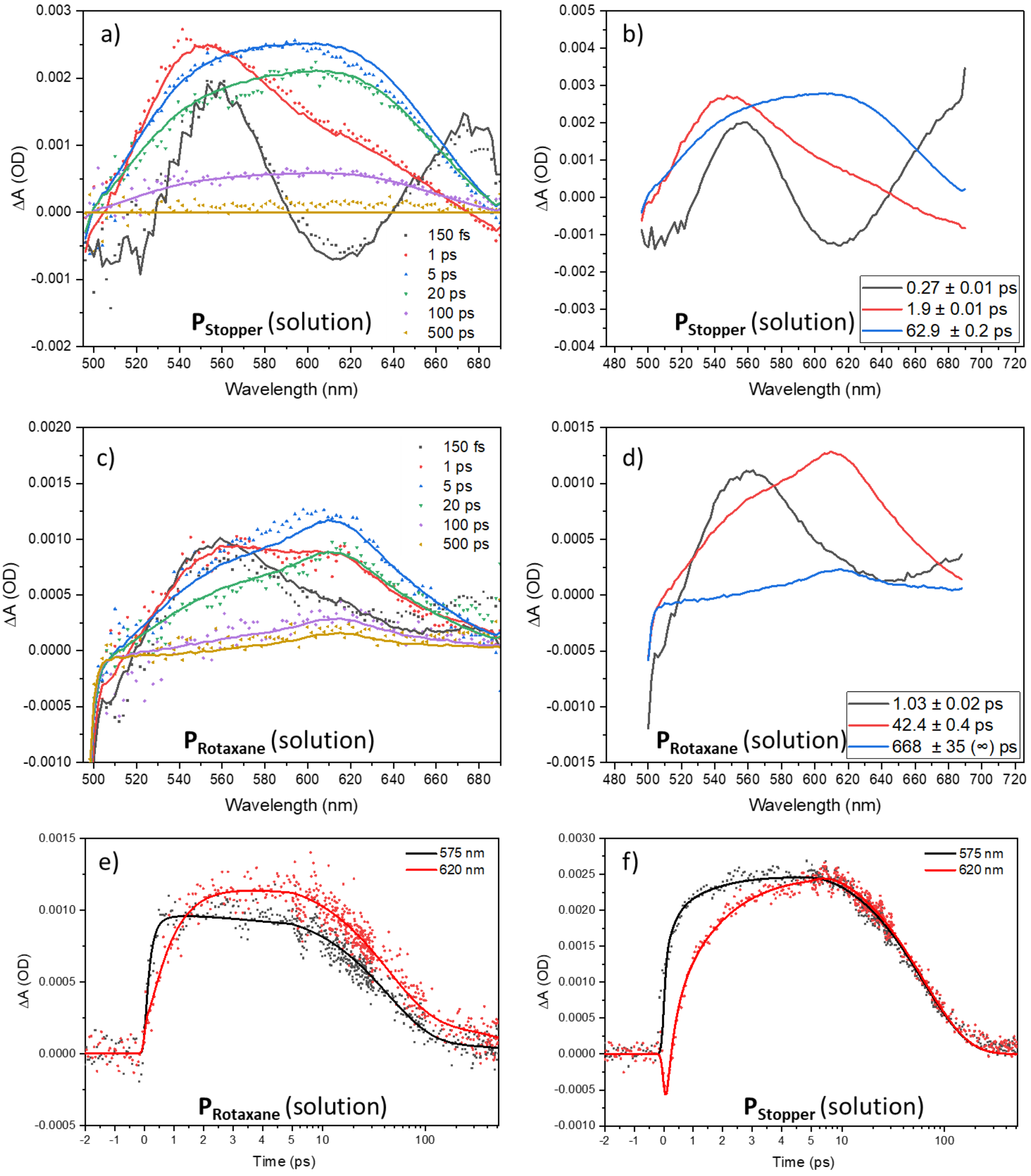

***Figure S25**. Transient absorption (TA, $\lambda_{exc.}$ = 480 nm) data and fits of **P$_{Rotaxane}$** and **P$_{Stopper}$** in supporting electrolyte (1.5 mL, 1 M LiTFSI valeronitrile/MeCN, 15:85) a) Transient absorption spectra at given time delays for **P$_{Rotaxane}$** and b) evolution associated spectra for **P$_{Rotaxane}$**. c) Transient absorption spectra at given time delays for **P$_{Stopper}$** and d) evolution associated spectra for **P$_{Stopper}$**. e) Kinetic traces for **P$_{Rotaxane}$** at 575nm (black) and 620 nm with (red). f) Kinetic traces for **P$_{Stopper}$** at 575nm (black) and 620 nm with (red).*

Table S1. *Time constants obtained from TA using a sequential model. Values indicated with ∞ are classified as infinity, because they are not substantially shorter than the experimental time range (500 ps). Dyes were measured in supporting electrolyte (1.5 mL, 1 M LiTFSI valeronitrile/MeCN,15:85 electrolyte).*

	**τ_1 (ps)**	**τ_2 (ps)**	**τ_3 (ps)**	**Model**
$\mathbf{P_{Stopper}}$ (solution)	0.27 ± 0.01	1.9 ± 0.01	62.9 ± 0.2	Sequential
$\mathbf{P_{Rotaxane}}$ (solution)	1.03 ± 0.02	42.4 ± 0.4	668 ± 35 (∞)	Sequential

- ***$P_{Rotaxane}$ and $P_{Stopper}$ on ZrO_2***

The TA spectra for the dyes on ZrO_2 are shown Figure S26. The data are well described by a sequential model with 3 components (see Tables S3 and S4 for lifetimes), the obtained evolution associated spectra are presented in Figure S26b and d. Figure S26a shows the TA spectra at various time delays for ZrO_2|$\mathbf{P_{Stopper}}$. The negative signal <550 nm is due to ground state bleach, while the positive photoinduced absorption signal >550 nm is due to $\mathbf{P_{Stopper}}$*****. The red shift with time indicates internal conversion, which may also explain the fast decay of the excited state absorption band around 680 nm.

Figure S26c shows the TA spectra at various time delays for ZrO_2|$\mathbf{P_{Rotaxane}}$. The negative signal <550 nm is due to ground state bleach, while the positive photoinduced absorption signal >550 nm is due to $\mathbf{P_{Rotaxane}}$*****. The red shift with time indicates intramolecular electron transfer (internal conversion), likely towards the **3-NDI-ring**. The transient signals decay due to intramolecular charge recombination.

To understand the difference in the TA signal around 620 nm observed for ZrO_2|$\mathbf{P_{Rotaxane}}$ and ZrO_2|$\mathbf{P_{Stopper}}$, we conducted time-resolved fluorescence experiments (TRPL, $\lambda_{exc.}$ = 532 nm). The results can be found in Figure S27 and Table S2. The signal for ZrO_2|$\mathbf{P_{Stopper}}$ is long-lived. The spectra for ZrO_2|$\mathbf{P_{Stopper}}$ red shift with time, likely due to structural relaxation of the dye. As compared to ZrO_2|$\mathbf{P_{Stopper}}$, the signal of ZrO_2|$\mathbf{P_{Rotaxane}}$ is short-lived, indicating intramolecular electron transfer from the dye part to the **3-NDI-ring**. The slow fluorescence decay component around 615 nm (Figure S27d) indicates that electron transfer from the dye moiety to the rotaxane-bound **3-NDI-ring** is hampered for a portion of the ZrO_2|$\mathbf{P_{Rotaxane}}$***** population. Structural inhomogeneity of $\mathbf{P_{Rotaxane}}$ might be the reason behind the fast and slow decay components around 615 nm. We envision that some $\mathbf{P_{Rotaxane}}$***** featuring the **3-NDI-ring** moiety is perfectly pre-organized for fast electron transfer, with the remaining $\mathbf{P_{Rotaxane}}$***** has conformations (rotaxane translation, rocking, circumrotating and pirouetting[14–16]) that hamper fast electron transfer from the dye part of ZrO_2|$\mathbf{P_{Rotaxane}}$***** to the **3-NDI-ring** part.

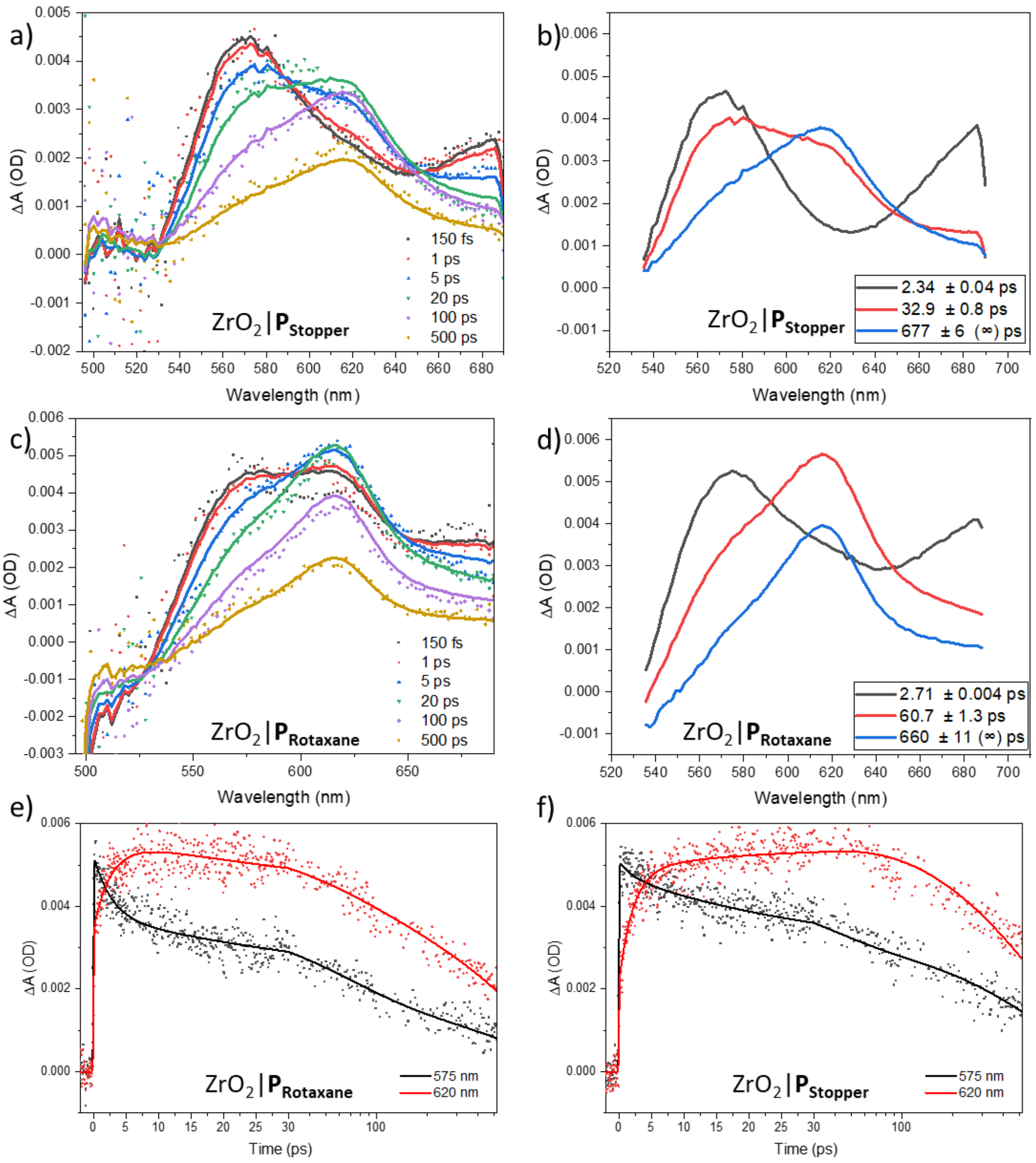

Figure S26. *Transient absorption (TA, $\lambda_{exc.}$ = 480 nm) data and fits for ZrO_2|**$P_{Stopper}$** and ZrO_2|**$P_{Rotaxane}$** in supporting electrolyte (1.5 mL, 1 M LiTFSI valeronitrile/MeCN, 15:85). a) Transient absorption spectra at given time delays for ZrO_2|**$P_{Stopper}$** and b) evolution associated spectra for ZrO_2|**$P_{Stopper}$**. c) Transient absorption spectra at given time delays for ZrO_2|**$P_{Rotaxane}$** and d) evolution associated spectra for ZrO_2|**$P_{Rotaxane}$** e) Kinetic traces for **$P_{Rotaxane}$** at 575nm (black) and 620 nm with (red). f) Kinetic traces for **$P_{Stopper}$** at 575nm (black) and 620 nm with (red).*

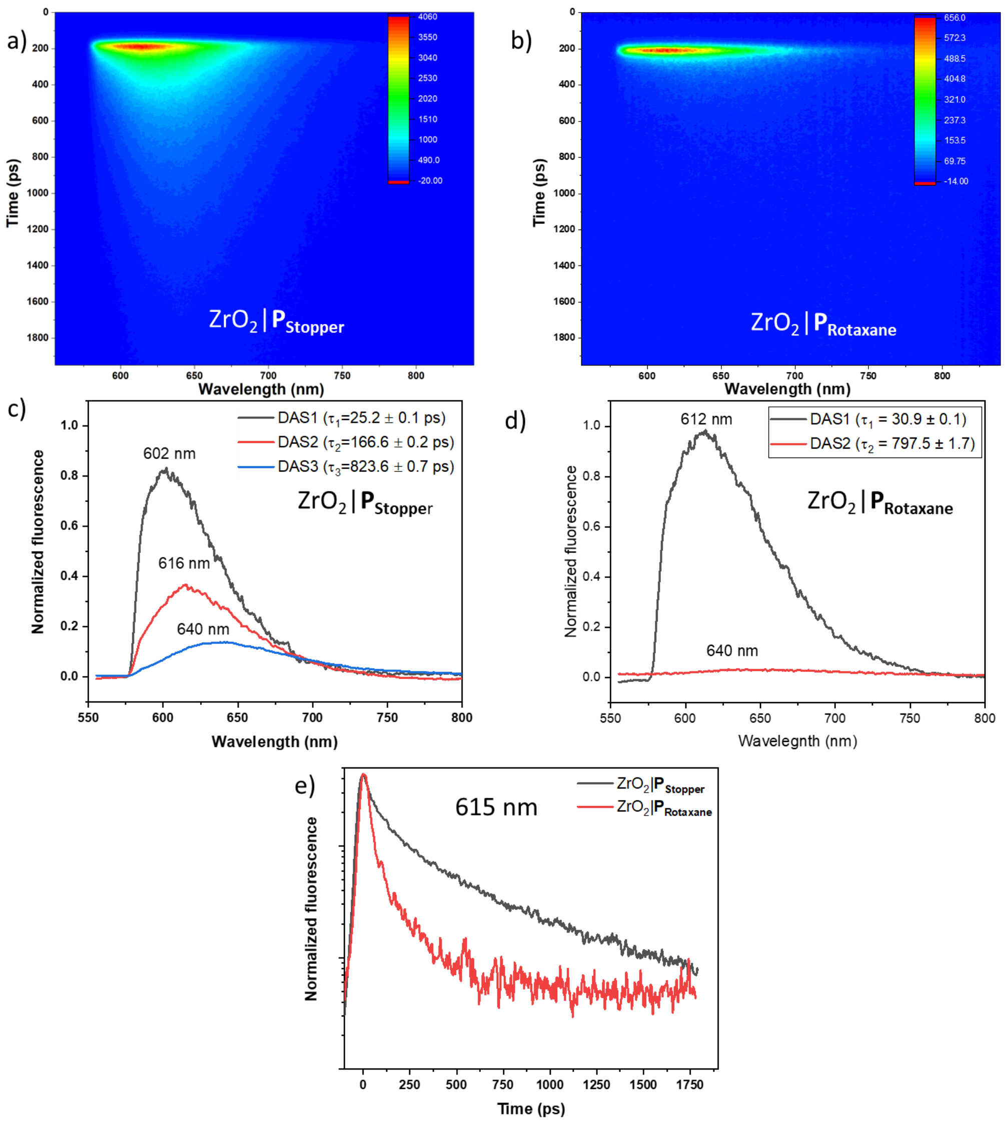

Figure S27. *Time-resolved fluorescence measurements ($\lambda_{exc.}$ = 532 nm) data of a)* ***$P_{Stopper}$*** *and b)* ***$P_{Rotaxane}$*** on *ZrO_2 in supporting electrolyte (1.5 mL, 1 M LiTFSI valeronitrile/MeCN, 15:85) (no additional* ***3-NDI-ring****). c) DAS for the ZrO_2|**$P_{Stopper}$**. d) DAS for the ZrO_2|**$P_{Rotaxane}$**. e) Decay of the fluoresence signal at 615 nm for both ZrO_2|**$P_{Stopper}$** and ZrO_2|**$P_{Rotaxane}$**.*

Table S2. *Time constants obtained from time-resolved fluorescence using a parallel model. The ZrO_2|**$P_{Stopper}$** and ZrO_2|**$P_{Rotaxane}$** were measured in supporting electrolyte (1 M LiTFSI valeronitrile/MeCN,15:85 electrolyte).*

	**τ_1 (ps)**	**τ_2 (ps)**	**τ_3 (ps)**	**Model**
ZrO_2|**$P_{Stopper}$**	25.2 ± 0.1	166.6 ± 0.2	823.6 ± 0.8	Parallel model
ZrO_2|**$P_{Rotaxane}$**	30.9 ± 0.1	797.5 ± 1.7		Parallel model

- ***$P_{Rotaxane}$ and $P_{Stopper}$ on NiO***

Figure S28c shows the TA spectra at various time delays for NiO|**$P_{Stopper}$**, which are discussed in detail in the main text. The data are well described by the photophysical model shown in Figure S31, the obtained lifetimes are presented in Table S3 and S4 and the decay associated spectra are shown in Figure S28d. Similarly, Figure S28e shows TA spectra at various time delays for NiO|**$P_{Stopper}$** in the presence of additional **3-NDI-ring** and Figure S28f shows the decay associated spectra from photophysical modeling for NiO| **$P_{Stopper}$**.

Figure S29c shows the TA spectra at various time delays for NiO|**$P_{Rotaxane}$**, which are discussed in detail in the main text. The data are well described by the photophysical model shown in Figure S32, the obtained lifetimes are presented in Table S3 and 4 and the decay associated spectra are shown in Figure S29d. Similarly, Figure S29e shows TA spectra at various time delays for NiO|**$P_{Rotaxane}$** in the presence of additional **3-NDI-ring** and Figure S29f shows the decay associated spectra from photophysical modeling for NiO|**$P_{Rotaxane}$**.

The photophysical model used for target analysis for NiO| **$P_{Stopper}$** + **3-NDI-ring** (Figure S31) NiO|**$P_{Rotaxane}$** (Figure S32) and NiO|**$P_{Rotaxane}$** + **3-NDI-ring** (Figure S32) deviates from the model in ref. [1] because the latter model did not give sensible time constants. Therefore, we decided to continue with the models represented in Figure S31 and Figure S32.

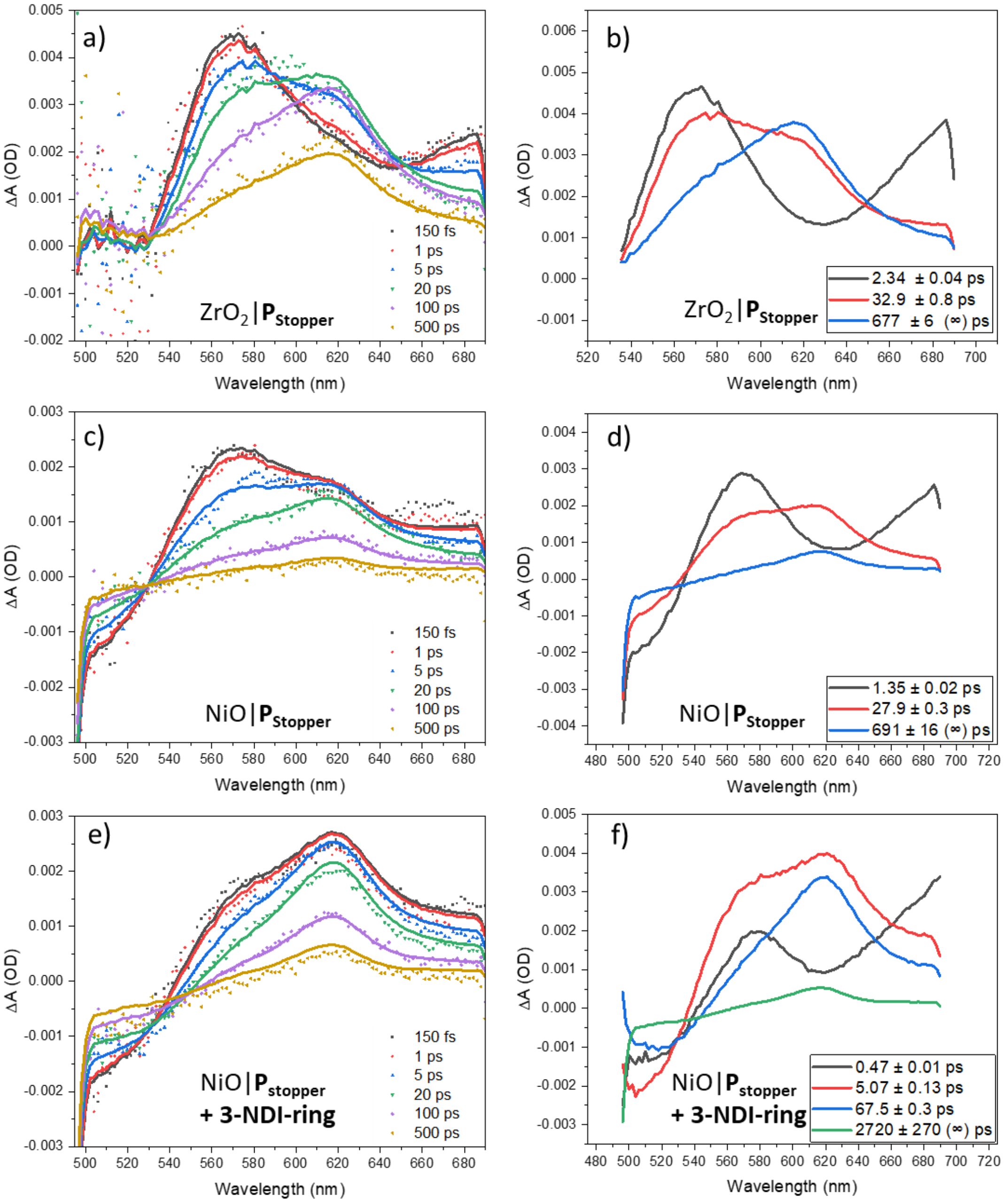

Figure S28. *Transient absorption (TA, $\lambda_{exc.}$ = 480 nm) data and fits for* **P**$_{Stopper}$ *on* ZrO_2 *and NiO in supporting electrolyte (1.5 mL, 1 M LiTFSI valeronitrile/MeCN, 15:85). a) Transient absorption spectra at given time delays for* ZrO_2|**P**$_{Stopper}$ *and b) evolution associated spectra for* ZrO_2|**P**$_{Stopper}$. *c) Transient absorption spectra at given time delays for NiO|***P**$_{Stopper}$ *and d) decay associated spectra for NiO|***P**$_{Stopper}$. *e) Transient absorption spectra at given time delays for NiO|***P**$_{Stopper}$ *with additional* ***3-NDI-ring*** *(5.9 mM) and f) decay associated spectra for NiO|***P**$_{Stopper}$ *with additional* ***3-NDI-ring****. Spectra are acquired in the presence of a 1 M LiTFSI valeronitrile/MeCN, 15:85 electrolyte without (a–d) and with* ***3-NDI-ring*** *redox mediator (5.9 mM) (e, f). The solid lines in the left panels indicate fits from photophysical modeling.*

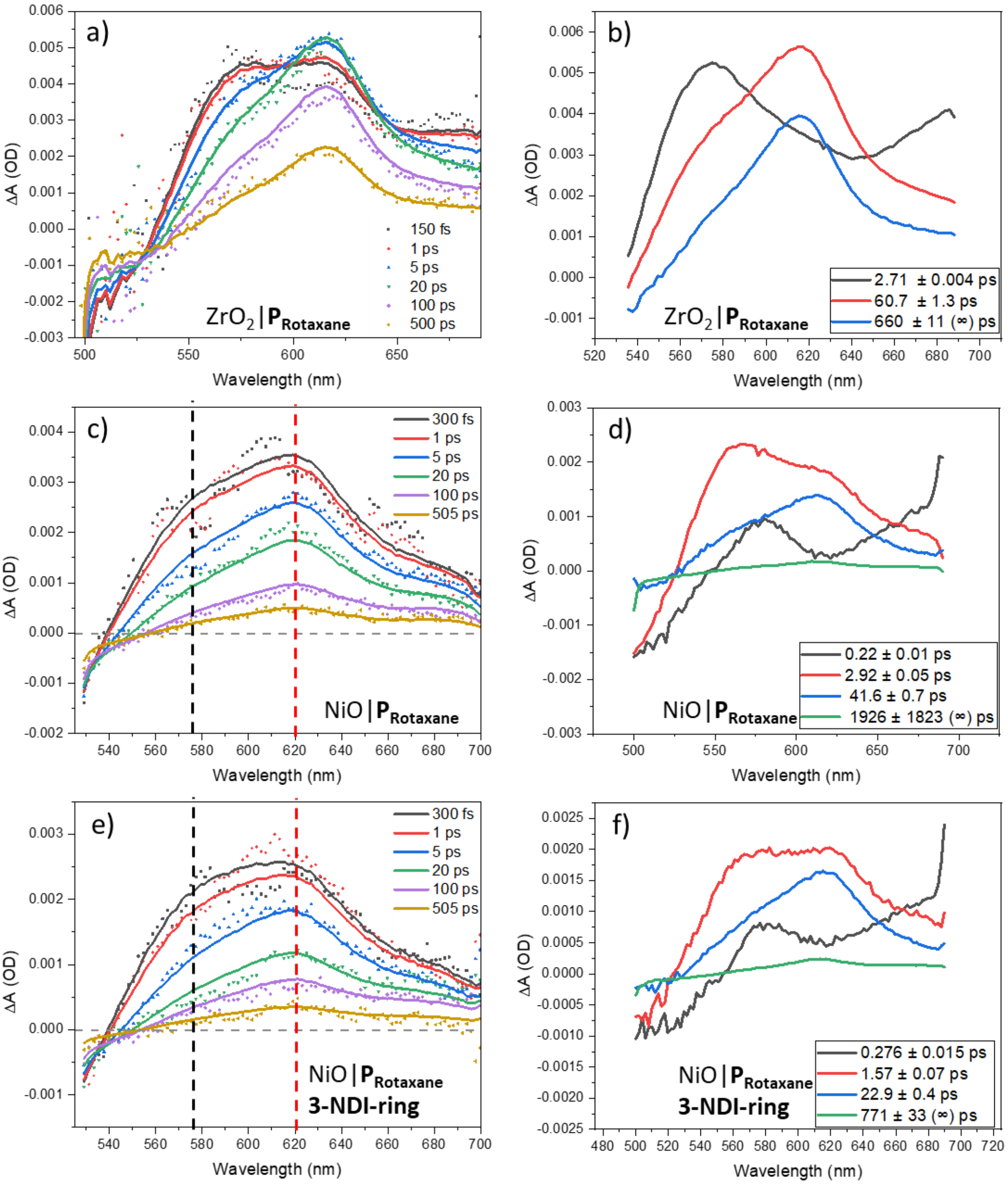

Figure S29. *Transient absorption (TA, $\lambda_{exc.}$ = 480 nm) data and fits for* $\mathbf{P}_{\mathbf{Rotaxane}}$ *on* ZrO_2 *and NiO in supporting electrolyte (1.5 mL, 1 M LiTFSI valeronitrile/MeCN, 15:85). a) Transient absorption spectra at given time delays for* $ZrO_2|\mathbf{P}_{\mathbf{Rotaxane}}$ *and b) evolution associated spectra for* $ZrO_2|\mathbf{P}_{\mathbf{Rotaxane}}$*. c) Transient absorption spectra at given time delays for NiO|*$\mathbf{P}_{\mathbf{Rotaxane}}$ *and d) decay associated spectra for NiO|*$\mathbf{P}_{\mathbf{Rotaxane}}$ *e) Transient absorption spectra at given time delays for NiO|*$\mathbf{P}_{\mathbf{Rotaxane}}$ *with additional* ***3-NDI-ring*** *(5.9 mM) and f) decay associated spectra for NiO|*$\mathbf{P}_{\mathbf{Rotaxane}}$ *with additional* ***3-NDI-ring****. Spectra are acquired in the presence of a 1 M LiTFSI valeronitrile/MeCN, 15:85 electrolyte without (a–d) and with* ***3-NDI-ring*** *redox mediator (5.9 mM) (e, f). The solid lines in the left panels indicate fits from photophysical modeling.*

Dye–mediator pre-organization effect

Figure 30 compares the presence of **3-NDI-ring** in case of **P1** and **P$_{Rotaxane}$**. The spectra indicate that there is no significant reduction of redox mediator observed in fs-TA if the mediator is not pre-organized to the dye as is the case for **P1**.

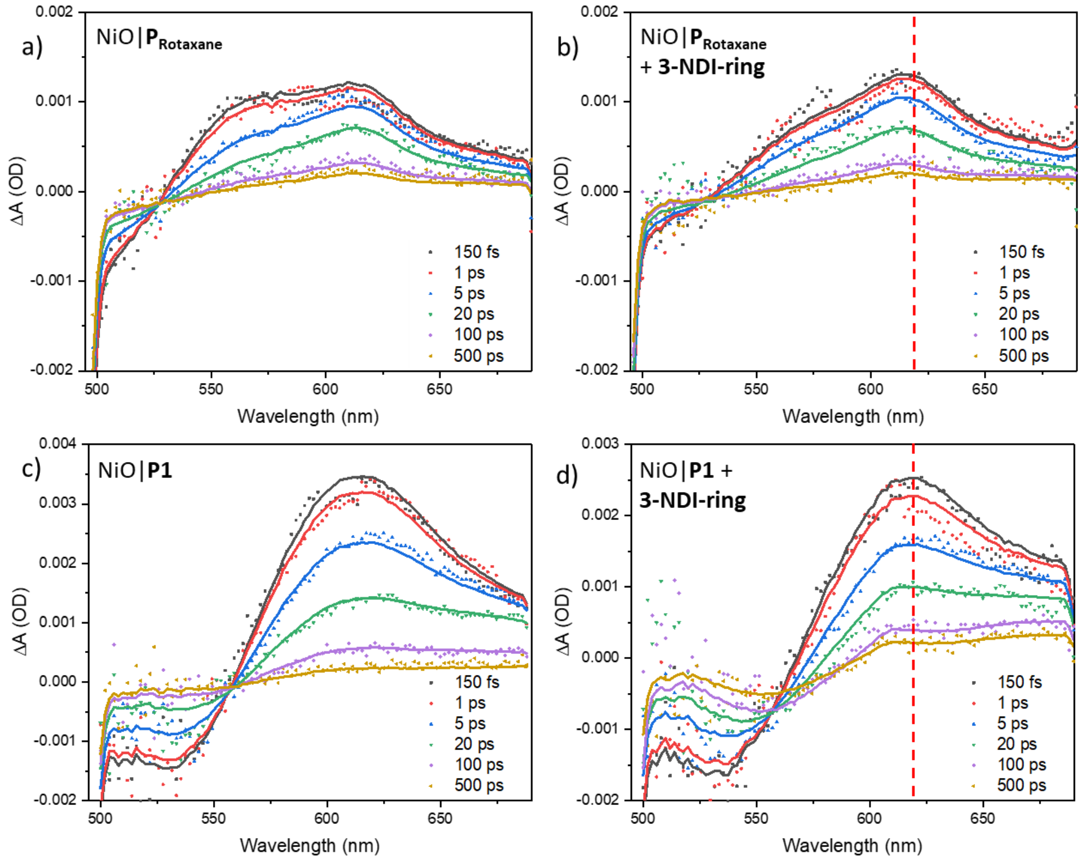

Figure S30. *Transient absorption (TA, $\lambda_{exc.}$ = 480 nm) data **P$_{Rotaxane}$** and **P1** on NiO in supporting electrolyte (1.5 mL, 1 M LiTFSI valeronitrile/MeCN, 15:85). a) Transient absorption spectra of NiO|**P$_{Rotaxane}$** at given time delays and b) Transient absorption spectra of NiO|**P$_{Rotaxane}$** at given time delays with additional **3-NDI-ring.** c) Transient absorption spectra of NiO|**P1** at given time delays and d) Transient absorption spectra of NiO|**P1** at given time delays with additional **3-NDI-ring**.*

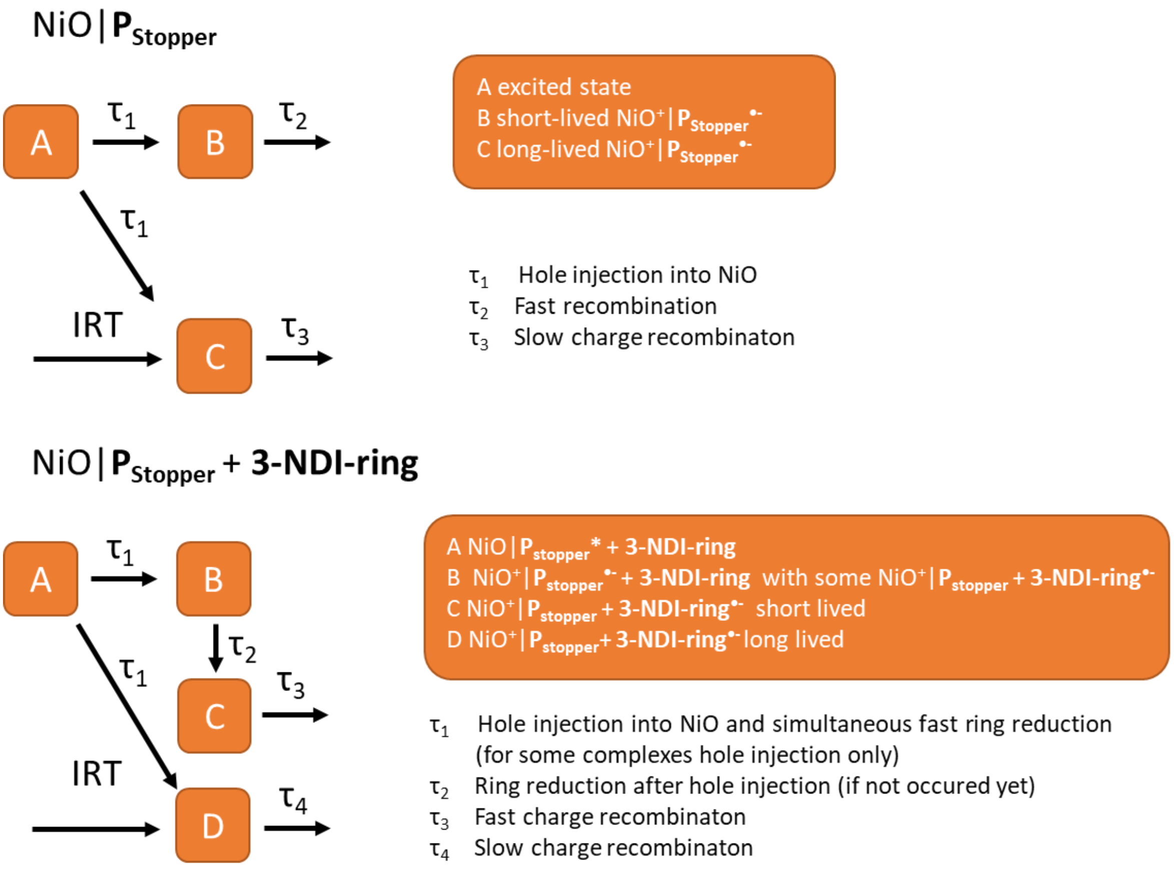

Figure S31. *Photophysical model used for target analysis for* $\mathbf{P}_{\mathit{Station}}$ *and* $\mathbf{P}_{\mathit{Station}}$ + **3-NDI-ring** *on mesoporous NiO.*

Table S3. *Time constants obtained from TA using the photophysical models indicated. Values indicated with ∞ are classified as infinity, because they are not substantially shorter than the experimental time range (500 ps). Dyes on thin mesoporous films (ZrO_2 and NiO) where measured in supporting electrolyte (1 M LiTFSI valeronitrile/MeCN,15:85 electrolyte) in absence or presence of the* **3-NDI-ring** *(5.9 mM).*

	**τ_1 (ps)**	**τ_2 (ps)**	**τ_3 (ps)**	**τ_4 (ps)**	**Model**
ZrO_2|$\mathbf{P}_{\mathbf{Stopper}}$	2.34 ± 0.04	32.9 ± 0.8	677 ± 6 (∞)		Sequential
NiO| $\mathbf{P}_{\mathbf{Stopper}}$	1.35 ± 0.02	-	27.9 ± 0.3	691 ± 16 (∞)	Figure S31
NiO| $\mathbf{P}_{\mathbf{Stopper}}$ with additional **3-NDI-ring** in the electrolyte	0.47 ± 0.013	5.07 ± 0.13	67.5 ± 0.3	2720 ± 270 (∞)	Figure S31

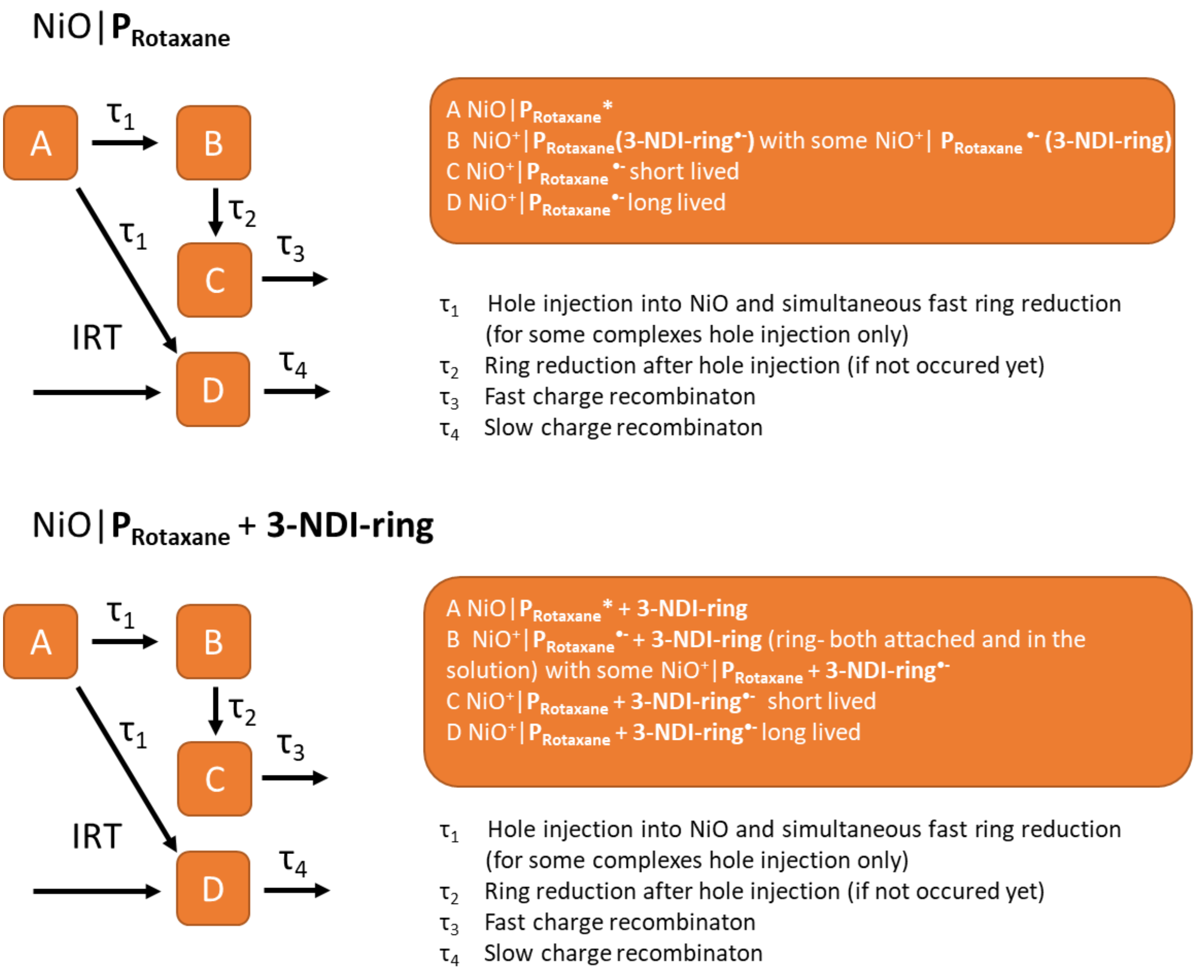

Figure S32. Photophysical model used for target analysis for **P$_{\textbf{Rotaxane}}$** and **P$_{\textbf{Rotaxane}}$** + **3-NDI-ring** on mesoporous NiO.

Table S4. *Time constants obtained from TA using the photophysical models indicated. Values indicated with ∞ are classified as infinity, because they are not substantially shorter than the experimental time range (500 ps). Dyes on thin mesoporous films (ZrO_2 and NiO) where measured in supporting electrolyte (1 M LiTFSI valeronitrile/MeCN, 15:85 electrolyte) in absence or presence of the* ***3-NDI-ring*** *(5.9 mM).*

	**τ_1 (ps)**	**τ_2 (ps)**	**τ_3 (ps)**	**τ_4 (ps)**	**Model**
ZrO_2|**P$_{\textbf{Rotaxane}}$**	2.71 ± 0.04	60.7 ± 1.3	660 ± 11 (∞)		Sequential
NiO|**P$_{\textbf{Rotaxane}}$**	0.21 ± 0.007	2.92 ± 0.05	41.7 ± 0.7	1926 ± 182 (∞)	Figure S32
NiO|**P$_{\textbf{Rotaxane}}$** with additional **3-NDI-ring** in the electrolyte	0.28 ± 0.015	1.57 ± 0.07	22.9 ± 0.4	771 ± 33 (∞)	Figure S32

S3. Device Fabrication and Characterization

S3.1. DSSC device Assembly

An elaborate description of working electrodes preparation and counter electrodes is reported in former work.[1] The same procedure and NiO paste were used in this study as is described in former work.[1] These plates were dipped in dye solution **P1** (0.3 mM in MeCN), $\mathbf{P_{Stopper}}$ or $\mathbf{P_{Rotaxaane}}$ (0.1 mM in MeCN). After 16 hours the plates are taken out of the solution and rinsed with MeCN to remove non-adsorbed dye. The plates changed color red for **P1** and orange for $\mathbf{P_{Stopper}}$ or $\mathbf{P_{Rotaxaane}}$. After air drying the plates are used for device assembly.

S3.2. DSSC Assembly Employing I^-/I_3^-

I^-/I_3^- Electrolyte preparation: LiI (138. mg, 1 mmol) and I_2 (25 mg, 0.1 mmol) were dissolved in 1 mL dry MeCN (Sigma-Aldrich) to obtain a 1 M electrolyte solution. The electrolyte was prepared fresh and used within 2 hours.

I^-/I_3^- Device assembly: The sandwich cells were prepared by melting the one-hole Pt counter electrode to the working electrode using Meltonix polymer 1170-60 (Solaronix, Switzerland, 60 μm) with a 350 °C heating source. The ends were coated with silver paste and left to dry for 30 minutes. Then the electrolyte was introduced by vacuum back filling. After cleaning and wiping the outside the hole was sealed with the Meltonix polymer 1170-60 and a glass cover slip. The cells were directly measured after fabrication.

S3.3. DSSC Assembly Employing 3-NDI-ring Electrolyte

3-NDI-ring electrolyte preparation: The electrolyte was prepared in a nitrogen filled glove box. To obtain the 25 mM 50:50 **3-NDI-ring/3-NDI-ring**$^{\bullet-}$ redox couple a Cobaltocene solution (0.5 mL, 12.5 mM in 1 M LiTFSI (valeronitrile:MeCN, 15:85 v/v) was added to 8.9 mg **3-NDI-ring**. The dark purple solution was stirred for 5 minutes and then led to set for 2 minutes to make sure that the oxidized Cobaltocene$^+$ precipitated. The electrolyte solution was prepared freshly for every new set of DSSCs and used within 2 hours.

3-NDI-ring device assembly: The sandwich cells were prepared by melting the one-hole Pt counter electrode to the working electrode using Meltonix polymer 1170-60 (Solaronix, Switzerland) of 60 μm thickness with a 350 °C heating source. The ends were coated with silver

paste and led to dry for 30 minutes. Then the electrolyte was introduced inside the glove box with a syringe. After cleaning and wiping the outside the hole was sealed with the Meltonix polymer 1170-60 and a glass cover slip. The cells were directly measured after fabrication.

S3.4. DSSC Assembly Employing 3-NDI-thread Electrolyte

Use of these DSSCs is described in SI section 5. The electrolyte was prepared in a nitrogen filled glove box. To obtain the 200 mM 50:50 **3-NDI-thread/3-NDI-thread**$^{\bullet-}$ redox couple a Cobaltocene solution (0.5 mL, 100 mM in 1 M LiTFSI (valeronitrile:MeCN, 15:85 v/v) was added to 53 mg **3-NDI-thread**. The dark purple solution was stirred for 5 minutes and then led to set for 2 minutes to make sure that the oxidized Cobaltocene^{+} precipitated. To obtain 100 mM, 100 μL of the 200 mM **3-NDI-thread/3-NDI-thread**$^{\bullet-}$ was diluted with 100 μL of 1 M LiTFSI valeronitrile:MeCN, 15:85 v/v. This dilution was repeated to obtain 50 mM and 25 mM **3-NDI-thread/3-NDI-thread**$^{\bullet-}$ electrolyte. The electrolyte solution was prepared freshly for every new set of DSSCs and used within 2 hours.

S3.5. DSSC Characterization

J–V characterization. Dye-sensitized solar cells (DSSCs) were characterized with *J–V* curves using the Oriel LCS-100 solar simulator. Before every measurement, the light intensity was set to 100 mW cm^{-2} with a calibrated silicon solar cell (Newport, 91150-2000). This reference photodiode consisted of an area of 2 cm × 2 cm calibrated monocrystalline silicon with a fused silica window and was equipped with a thermocouple which is assembled in accordance with IEC 60904-2. The certification is accredited by the National Renewable Energy laboratory to the ISO-17025 standard conducted by the PV lab at Newport Corporation. Spectral Mismatch correction factor M = 0.996 ± 0.004 is reported. The current-voltage characteristics of the DSSCs were measured by applying an external potential bias and measuring the generated current with a potentiostat PGSTAT302N from Autolab with a 5 mV s^{-1} scan rate and 0.488 s hold time. Measurements for cells with **3-NDI-ring** electrolyte was from +0.480 V to -0.01V (170 points) for $\mathbf{P_{Stopper}}$ and +0.420 V to -0.01V (100 points) for $\mathbf{P_{Rotaxane}}$. DSSCs employing the I^-/I_3^- electrolyte were scanned from 0.140 V to -0.01 V (60 points) for $\mathbf{P_{Rotaxane}}$ and $\mathbf{P_{Stopper}}$. Characterization was performed at room temperature (20°C) under ambient conditions.

IPCE. IPCE was measured with the Zahner Tunable Optical Light Source TLS03 starting from 414–723 nm (resolution = 1 nm) in continuous mode with phase at 0.1 Hz and 5 counts. Characterization was performed at ambient temperature (20°C).

Electrical Impedance spectroscopy. Electrical impedance spectroscopic (EIS) measurements were performed with the Zahner LSW-2 light source controlled by Zahner PP211 potentiostat. The measurements were performed at different light intensities (120, 100, 80, 60, 40, 30, 25, 20, 15, 10 and 5 mW cm^{-2}) from 1–100 KHz to lower limit 0.1 Hz at an amplitude of 5 mV. Characterization was done at ambient temperature (20°C).

Chopped light amperometry. Chopped light voltammetry was performed with the Zahner LSW-2 light source controlled by Zahner PP211 potentiostat. The light source intensity was programmed to perform on/off cycles of 10 seconds while every cycle the light intensity was increased from 5 to 10 10 mW cm^{-2} , then increased with 10 mW cm^{-2} starting from 10 mW cm^{-2} and ending at 130 mW cm^{-2}. Characterization was done at ambient temperature (20°C).

S3.6. Photovoltaic Performance Employing I^-/I_3^- Electrolyte

DSSCs were prepared using screen printed NiO photocathodes (3.5 µm, 0.196 cm^{-2}) that were sensitized with **P_{Stopper}** or **P_{Rotaxane}** MeCN solution for 16 hours. To enable a fair comparison between **$P_{Stopper}$** or **$P_{Rotaxane}$** DSSCs, first devices were prepared using the I^-/I_3^- electrolyte (0.9:0.1 M in MeCN) The devices were assembled with Pt counter electrode using a 60 µm thermoplast frame. The photovoltaic performances of DSSCs based on **$P_{Rotaxane}$** and **$P_{Stopper}$** employing I^-/I_3^- electrolyte under AM1.5G illumination (100 mW cm^{-2}) are represented in Table S5 and Figure S33. The performance of both dyes is very similar in terms of **$P_{Rotaxane}$** PCE (0.035%) in comparison to **$P_{Stopper}$** (0.031%) reflected by a small difference in V_{OC} (4 mV difference) and J_{SC} (0.08 mA cm^{-2} difference). The IPCE spectrum also reflects is similarity in performance between the **$P_{Stopper}$** (13.5 % at λ=468 nm) or **$P_{Rotaxane}$** (14.6% at λ=490 nm) The performance of DSSC using **$P_{Stopper}$** or **$P_{Rotaxane}$** employing I^-/I_3^- implies that in absence of dye–mediator interactions the systems are very similar and differences are minimal.

Table S5. Summary of the photovoltaic performance data for DSSC based on **$P_{Stopper}$**, **$P_{Rotaxane}$** and **P1** as benchmark system under AM 1.5G illumination (100 mW cm^{-2}) with the I^-/I_3^- electrolyte (1 M in MeCN). The average performance (N=4 for **$P_{Stopper}$**, **$P_{Rotaxane}$** and N=5 for **P1**) is provided with the best performing cell in brackets.

Dye	V_{OC} (V)	J_{SC} (mA cm^{-2})	FF	PCE (%)
**$P_{Stopper}$**	0.08±0.004 (0.08)	-1.41±0.07 (-1.48)	0.297±0.006 (0.300)	0.031±0.003 (0.035)
**$P_{Rotaxane}$**	0.08±0.04 (0.10)	-1.49±0.18 (-1.67)	0.296±0.006 (0.302)	0.035±0.004 (0.039)
**P1**	0.11 ± 0.003 (0.11)	-1.92 ± 0.05 (1.92)	0.296 ± 0.003 (0.298)	0.061 ± 0.002 (0.063)

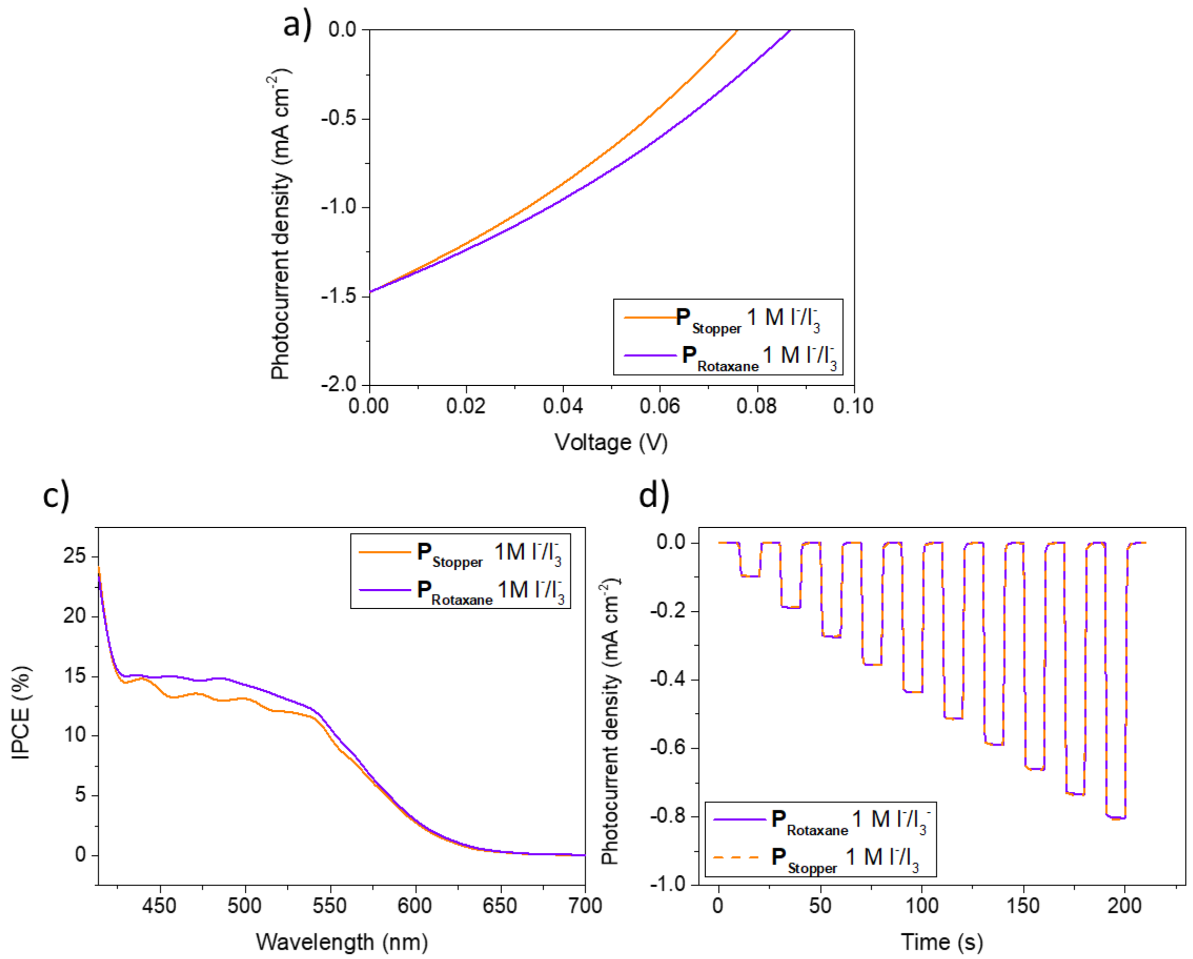

Figure S33. *Photovoltaic performances of the devices based on the* ***P****$_{stopper}$ (orange line) and the* ***P****$_{Rotaxane}$ dye (violet line) employing I^-/I_3^-as redox mediator (1 M). a) J–V curves b) Photocurrent action spectrum. c) Chopped light amperometry at different light flux varying from 5–130 mW cm^{-2} with on/off cycles of 10 seconds. The performance of* ***P****$_{Rotaxane}$ and* ***P****$_{stopper}$ shows overlap, indicating the similarity of these systems when I^-/I_3^-as redox mediator is employed.*

Statistics on DSSCs employing I^-/I_3^-

Table S6. *Overview of results obtained from J–V curves under with AM 1.5G illumination (100 mW cm^{-2}) of all I^-/I_3^- DSSCs that were prepared during this study.*

sc#	Dye	V_{oc} (V)	J_{sc} (A cm^{-2})	FF	PCE (%)
sc0427	$\mathbf{P_{Stopper}}$	0.075	-1.35E-03	0.299	0.030
sc0428	$\mathbf{P_{Stopper}}$	0.078	-1.48E-03	0.300	0.035
sc0429	$\mathbf{P_{Stopper}}$	0.075	-1.36E-03	0.296	0.030
sc0430	$\mathbf{P_{Stopper}}$	0.070	-1.48E-03	0.291	0.030
sc0431	$\mathbf{P_{Rotaxane}}$	0.070	-1.48E-03	0.291	0.030
sc0432	$\mathbf{P_{Rotaxane}}$	0.070	-1.48E-03	0.291	0.030
sc0433	$\mathbf{P_{Rotaxane}}$	0.088	-1.50E-03	0.298	0.039
sc0434	$\mathbf{P_{Rotaxane}}$	0.090	-1.33E-03	0.302	0.036
sc0436	$\mathbf{P_{Rotaxane}}$	0.076	-1.67E-03	0.299	0.038

Table S7. *Statistics of all* $\mathbf{P_{Stopper}}$ *DSSCs under study based on Supplementary Table S6.*

		V_{oc} (V)	J_{sc} (A cm^{-2})	FF	PCE (%)
$\mathbf{P_{Stopper}}$	Mean	0.075	-1.41E-03	0.297	0.031
	STDEV	0.003	6.37E-05	0.004	0.002
	Error	0.001	3.19E-05	0.002	0.001

Table S8. Statistics of all $\mathbf{P_{Rotaxane}}$ DSSCs under study based on Supplementary Table S6.

		V_{oc} (V)	J_{sc} (A cm^{-2})	FF	PCE (%)
$\mathbf{P_{Rotaxane}}$	Mean	0.079	-1.49E-03	0.296	0.035
	STDEV	0.009	1.07E-04	0.004	0.004
	Error	0.004	4.81E-05	0.002	0.002

S3.7. Electrochemical Impedance Spectroscopy on DSSCs employing I^-/I_3^-

The origins of the performance of DSSC using $\mathbf{P_{Stopper}}$ or $\mathbf{P_{Rotaxane}}$ employing I^-/I_3^- was further investigated with EIS, a widely applied method to study kinetics of electrochemical and photochemical processes,[17–19] and specifically in *p*-DSSCs as described in various reports[20–23] and a review.[24] In *n*-DSSC the three semicircles represented by the Nyquist diagram, can be assigned to three separate processes: charge transfer at the counter electrode in the kHz regime (1), recombination at the semiconductor interface typically 1–100 Hz (2) and diffusion within the electrolyte at the mHz region (3). These semicircles can be assigned by fitting circuit elements or a combination of multiple elements that represent the physical processes such as the successful transmission line model by Bisquert and coworkers, which can also be applied to *p*-DSSC.[25,26] The Nyquist plots obtained under 60 mW cm^{-2} irradiation are represented in Figure S34a and the measured data were fitted with an equivalent circuit build from a Randles circuit that represents the series resistance and the counter electrode resistance (at lower impedance, first wave) and a transmission line element that represents the photoelectrode (at higher impedance, second wave).[25] By performing EIS measurements under varying light intensities at open voltage, the chemical capacitance (C_μ) and recombination resistance (R_{REC}) as a function of potential can be obtained.[26] The C_μ in both cases shows an identical flat line that does not vary under different bias conditions (Figure S34b), which is common for NiO and was also observed by Wu and coworkers for the **P1** dye in combination with the same I^-/I_3^- electrolyte.[20] However, differences between $\mathbf{P_{Stopper}}$ and $\mathbf{P_{Rotaxane}}$ were found for the R_{REC} (Figure S34c), which is 2 times higher for $\mathbf{P_{Rotaxane}}$ (28 Ω cm^{-2} at 100 mV) compared to $\mathbf{P_{Stopper}}$ (15 Ω cm^{-2} at 100 mV). This two-fold difference implies less recombination for $\mathbf{P_{Rotaxane}}$ system translating in an almost two times increase in hole lifetime for $\mathbf{P_{Rotaxane}}$ (50 ms at 100 mV) compared to $\mathbf{P_{Stopper}}$ (29 ms at 100 mV) at a given voltage (Figure S34d). This difference in hole lifetime indicates that the $\mathbf{P_{Rotaxane}}$ has a small benefit from the **3-NDI-ring** being permanently installed on the dye. Satisfied that the difference in performance is understood as a consequence of the **3-NDI-ring** being mechanically bound to the dye, we next turned to DSSCs employing **3-NDI-ring** as redox mediator, making the differences in subsequent DSSCs described below attributable to pre-organization and ring-launching effects.

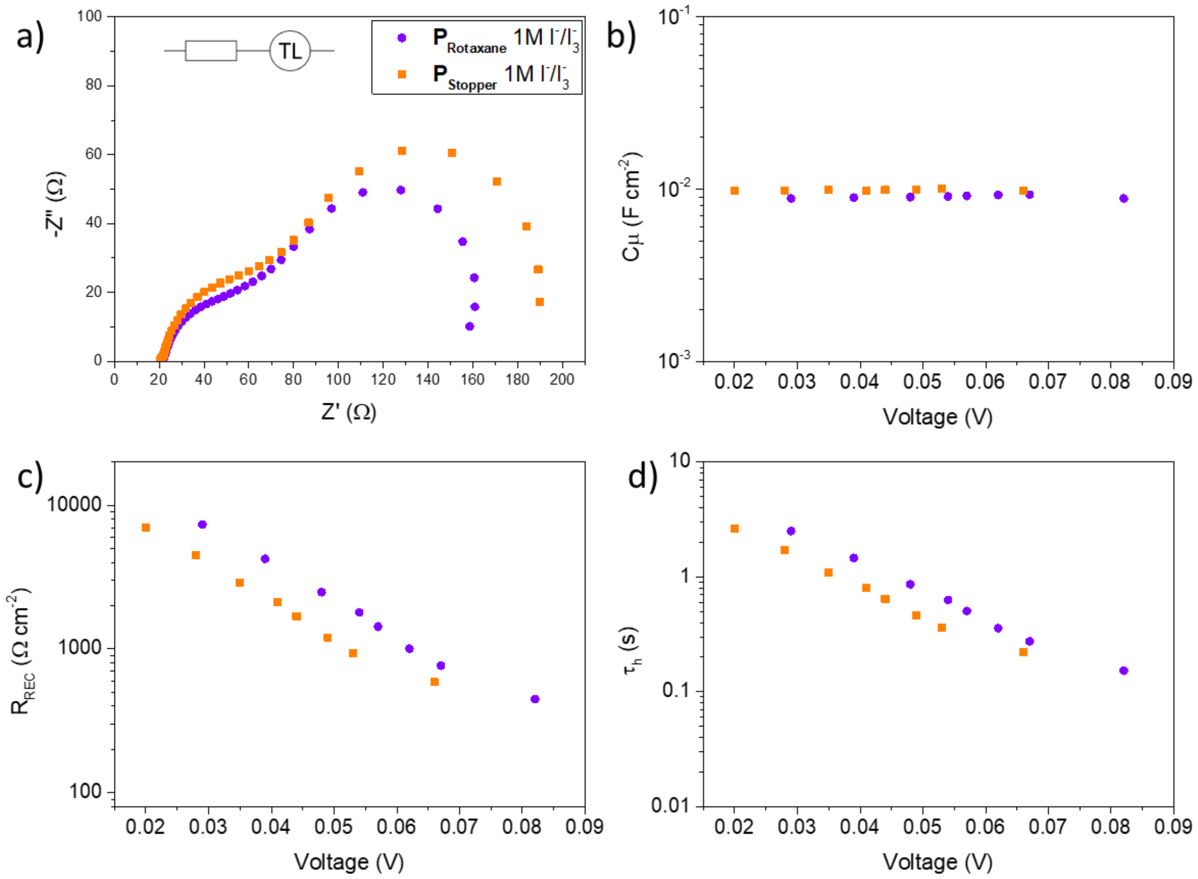

Figure S34. *EIS measurements under varying light intensities measured at open voltage for* $\mathbf{P}_{\mathbf{Rotaxane}}$ *(violet rounds) and* $\mathbf{P}_{\mathbf{Stopper}}$ *(orange squares) DSSC employing 1 M* I^-/I_3^- *electrolyte. The data was analyzed with a model consisting of transition line model. a) Nyquist plot under 60 mW* cm^{-2}*showing two characteristic semicircles. b) Chemical capacitance showing a straight line for both systems c) Recombination parameters as a function of voltage. d) Hole lifetime.*

S3.8. Characterization DSSCs Employing 3-NDI-ring

*Statistics on DSSCs employing **3-NDI-ring***

***Table S9.** Overview of results obtained from J–V curves under with AM 1.5G illumination (100 mW cm^{-2}) of all **3-NDI-ring** DSSCs that were prepared during this study.*

sc#	Dye	V_{oc} (V)	J_{sc} (A cm^{-2})	FF	PCE (%)
sc0416	$\mathbf{P_{Stopper}}$	0.408	-3.76E-04	0.442	0.068
sc0417	$\mathbf{P_{Stopper}}$	0.403	-3.41E-04	0.405	0.056
sc0419	$\mathbf{P_{Stopper}}$	0.380	-4.17E-04	0.445	0.071
sc0444	$\mathbf{P_{Stopper}}$	0.328	-3.16E-04	0.392	0.041
sc0453	$\mathbf{P_{Stopper}}$	0.376	-3.94E-04	0.405	0.060
sc0454	$\mathbf{P_{Stopper}}$	0.375	-3.62E-04	0.404	0.055
sc0488	$\mathbf{P_{Stopper}}$	0.338	-3.44E-04	0.388	0.045
sc0489	$\mathbf{P_{Stopper}}$	0.331	-2.72E-04	0.401	0.036
sc0476	$\mathbf{P_{Stopper}}$	0.326	-3.17E-04	0.396	0.041
sc0477	$\mathbf{P_{Stopper}}$	0.338	-3.00E-04	0.398	0.040
sc0478	$\mathbf{P_{Stopper}}$	0.331	-3.14E-04	0.402	0.042
sc0447	$\mathbf{P_{Rotaxane}}$	0.423	-3.76E-04	0.403	0.064
sc0492	$\mathbf{P_{Rotaxane}}$	0.425	-3.89E-04	0.378	0.063
sc0493	$\mathbf{P_{Rotaxane}}$	0.441	-3.90E-04	0.398	0.068
sc0494	$\mathbf{P_{Rotaxane}}$	0.428	-3.70E-04	0.371	0.059
sc0495	$\mathbf{P_{Rotaxane}}$	0.403	-3.97E-04	0.401	0.064
sc0496	$\mathbf{P_{Rotaxane}}$	0.466	-3.96E-04	0.380	0.070
sc0479	$\mathbf{P_{Rotaxane}}$	0.441	-4.02E-04	0.430	0.076
sc0480	$\mathbf{P_{Rotaxane}}$	0.403	-3.55E-04	0.431	0.062

***Table S10.** Statistics of all $\mathbf{P_{Stopper}}$ DSSCs under study based on Supplementary Table S9.*

		V_{oc} (V)	J_{sc} (A cm^{-2})	FF	PCE (%)
$\mathbf{P_{Stopper}}$	MEAN	0.358	-3.41E-04	0.407	0.050
	STDEV	0.029	4.26E-05	0.020	0.012
	ERROR	0.010	1.51E-05	0.007	0.004

***Table S11.** Statistics of all $\mathbf{P_{Rotaxane}}$ DSSCs under study based on Supplementary Table S9.*

		V_{oc} (V)	J_{sc} (A cm^{-2})	FF	PCE (%)
$\mathbf{P_{Rotaxane}}$	MEAN	0.431	-3.84E-04	0.399	0.066
	STDEV	0.021	1.61E-05	0.023	0.005
	ERROR	0.007	5.68E-06	0.008	0.002

S3.9. Electrochemical Impedance Spectroscopy on DSSCs Employing 3-NDI-ring

To further understand the suppression of recombination in devices based on $\mathbf{P_{Rotaxane}}$ Electrochemical Impedance Spectroscopic (EIS) measurements were carried out at different light intensities. The results were analyzed using the transmission line model with addition of a Warburg element to simulate electrolyte diffusion (Figure S35).[25]

Nyquist diagram (Figure 35a) barely shows the semicircle but show a 45° line instead which is associated with diffusion. The diffusion (D_e) of redox mediators in devices employing macrocycles (25 mM **3-NDI-ring**, $D_e = 1.4 \times 10^{-10}$ m^2 s^{-1}) is ~4 times slower than for I^-/I_3^- (1 M, $D_e = 4.85 \times 10^{-10}$ m^2 s^{-1}) [19] This difference in diffusion is in line with the mass-transfer limitations observed in the chopped light experiments (Figure 8c). Furthermore, as the *r* of iodide (*r* = 0.2 nm)[27] is 3.5 times smaller than **3-NDI-ring** (*r* = 0.7 nm, Figure S11) the former is expected to experience faster diffusion kinetics. The chemical capacitance C_μ reveals a slight upwards (28 mV) shift of the valance band for $\mathbf{P_{Rotaxane}}$ (Figure S35), which can be assigned to the difference in dye loading, leaving less NiO sites exposed to interact with ions present in the electrolyte.[20,25] The recombination resistance (R_{REC}) is double for $\mathbf{P_{Rotaxane}}$ devices (40.8 × 10^3 Ω cm^{-2} at 100 mV) compared to those based on $\mathbf{P_{Stopper}}$ (21.8 × 10^3 Ω cm^{-2} at 100 mV) implying less recombination occurs at the NiO–dye interface in the $\mathbf{P_{Rotaxane}}$ *p*-DSSCs. This difference in R_{REC} (at any given voltage) translates into a 50% increase in hole lifetime for $\mathbf{P_{Rotaxane}}$ *p*-DSSCs (811 ms at 100 mV) compared to devices based on $\mathbf{P_{Stopper}}$ (527 ms at 100 mV). The decrease in recombination in the $\mathbf{P_{Rotaxane}}$ system compared to $\mathbf{P_{Stopper}}$, translates into an extended hole lifetime, leading to a higher V_{OC}, J_{SC} and PCE.

To demonstrate the effect of the rotaxane-based dye design on the charge recombination, the hole lifetimes (at 100 mV) of the devices based on the different **P1**-derived dyes $\mathbf{P_{Rotaxane}}$ and $\mathbf{P_{Stopper}}$ are summarized together with PCE and dye loading in Table 3. The *p*-DSSC devices using I^-/I_3^- electrolytes were also assessed to serve as a point of reference, being a system exhibiting no diffusional limitation.

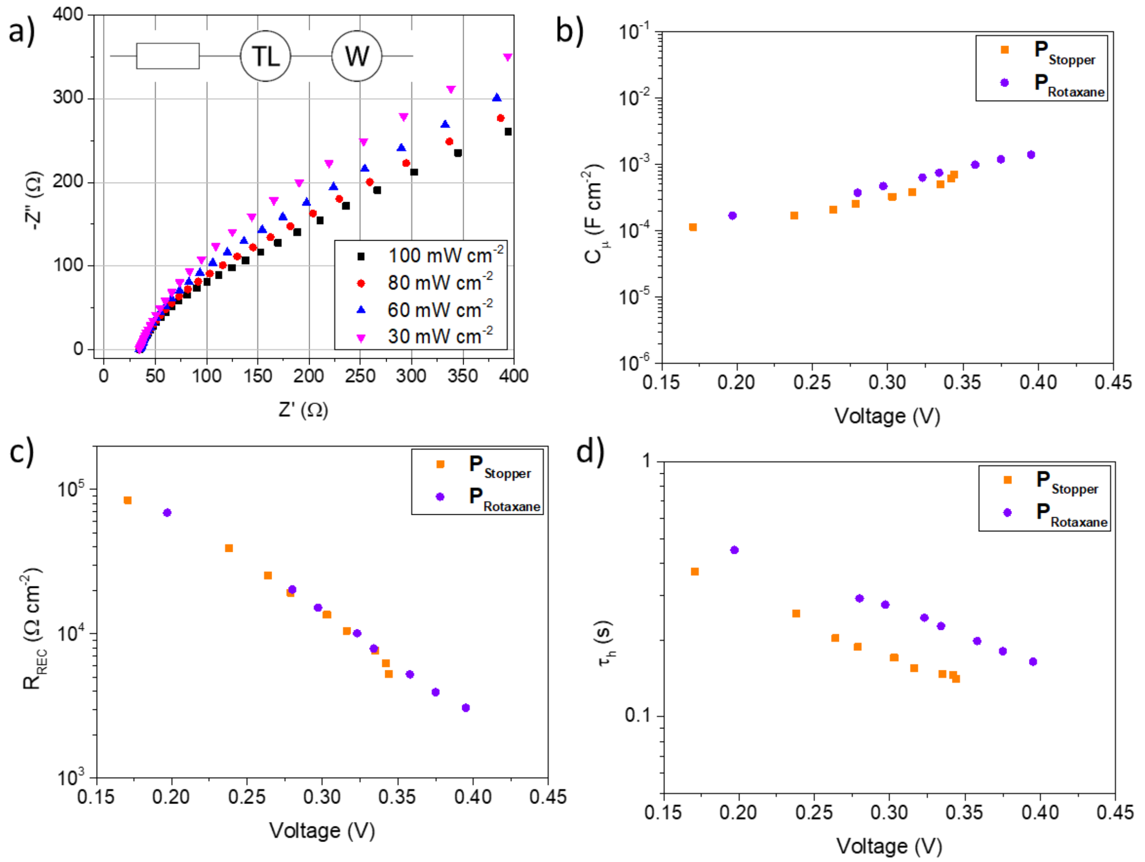

Figure S35. *EIS measurements under varying light intensities measured at open voltage for* $\boldsymbol{P_{Rotaxane}}$ *(violet rounds) and* $\boldsymbol{P_{Stopper}}$ *(orange squares) DSSC employing* ***3-NDI-ring*** *electrolyte (25 mM). The data was analyzed with a model consisting of transition line model and a Warburg element. a) Nyquist plot under 100, 80, 60, 30 mW cm*$^{-2}$*showingpart of a circle that become a 45° line. b) Chemical capacitance showing a descending line c) Recombination parameters as a function of voltage. d) Hole lifetime.*

S3.10. Calculation of limiting photocurrent

To demonstrate that we inhibited the recombination, we are determining the theoretical maximum current that we can expect in our device (J_{lim}) based on based on the redox mediator of choice and its concentration. From the concentration and diffusion coefficient of the redox mediator we can determine the mass transfer. The J_{lim} is independent of dye system and is solely dictated by the properties of the electrolyte, (thus J_{lim} is not photocurrent, just current).

The calculated J_{lim} is compared with the photocurrents at short circuit that we observe (J_{SC}) in our supramolecular dye-system (**3-NDI-ring/3-NDI-ring$^{\bullet -}$**) and traditional iodide-systems (I^-/I_3^-).

Equation 5 follows from a theoretical analysis based on electrolyte mass transfer relating the diffusion coefficient *D* and concentration *C** limiting the theoretical current density.[19] Using this equation the theoretical current can be calculated based on mass limitations.

$$J_{lim,(3-\boldsymbol{NDI-ring})} = f\,\frac{F\,D_{(3-\boldsymbol{NDI-ring})}\,C^{*}_{(3-\boldsymbol{NDI-ring})}}{d_{EL}} \qquad (5)$$

In Equation 5

- d_{EL} represents the distance between the working electrode and the counter electrode (60×10^{-6} m).
- *C** represents the concentration of the diffusion limiting species (**3-NDI-ring/3-NDI-ring$^{\bullet -}$**,12.5:12.5 mM; I^-/I_3^- 900:100 mM). Therefore C***3-NDI-ring$^{\bullet -}$** =12.5 mM; C* I_3^- = 100 mM)
- The factor *f* is 2 for **3-NDI-ring** versus *f* = 4 for I^-/I_3^-
- This gives: $J_{lim(\mathbf{3\text{-}NDI\text{-}ring})}$ = 0.6 mA cm^{-2} I^-/I_3^- ($J_{lim(I3-)}$ = 95 mA cm^{-2}).

Table S12. *The values used in Equation 5 to calculate J_{lim} to compare this to the photocurrent J_{SC}.*

Electrolyte type	Concentration limiting species C* (mM)	Diffusion coefficient *D* (cm^2 s^{-1})	J_{lim} (calculated) (mA cm^{-2})	J_{SC} (measured for $\mathbf{P_{Rotaxane}}$) (mA cm^{-2})	J_{SC} (measured for $\mathbf{P_{stopper}}$) (mA cm^{-2})
**3-NDI-ring/3-NDI-ring$^{\bullet -}$**	12.5	1.40 ×10^{-6}	-0.56	-0.39	-0.34
I^-/I_3^-	100	4.85 ×10^{-6}	-95.6	-1.49	-1.41

Based on the values represented in Table S12 we determined that under these concentration DSSC employing the **3-NDI-ring** reaches as much as $\mathbf{P_{stopper}}$ 60% of the theoretical current and for $\mathbf{P_{Rotaxane}}$ this is almost 70%, while for I^-/I_3^- electrolyte (I^-/I_3^- 0.9:0.1 M) in MeCN cells this is only 1.6%. The fact that the obtained photocurrents approach the limited current, implies that recombination has been suppressed to a minimum and the system based on supramolecular interactions is mostly limited by slow diffusion of the large macrocyclic mediator.

S3.11. Investigation of diffusion limitation with the 3-NDI-based electrolyte

Recombination is suppressed to a minimum in devices with **$P_{Rotaxane}$** employing **3-NDI-ring** as mediator. However, the chopped light amperometry (Figure 8) suggested mass transfer limitation caused by low concentration and slow diffusion of the redox mediator. Low solubility of the **3-NDI-ring** does not allow for higher concentration than 25 mM. The diffusion limitation with **3-NDI-ring** devices is further investigated with **3-NDI-thread** and the **$P_{Stopper}$** dye as model system. This system also promotes dye–mediator interactions between **3-NDI-thread** and the **DNP**-arms of **$P_{Stopper}$** (Figure S36). Binding of **3-NDI-thread** to the **DNP**-arm within **$P_{Stopper}$** were prohibited by limited solubility of the dye, therefore the recognition site moiety **DNP-fragment** (Figure S21a) was used to analyze the interaction strength by ^{1}H NMR titration (K_{ass} = 5 M^{-1}, Figure S21).

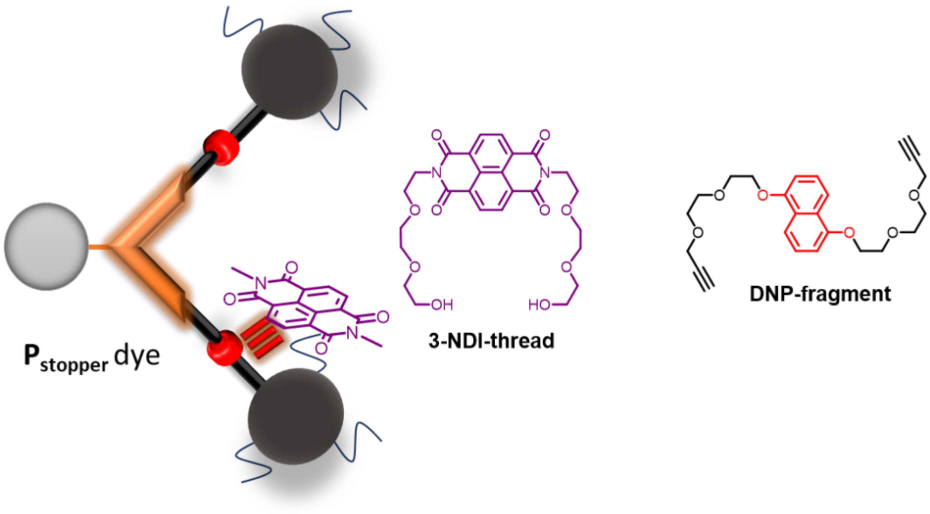

Figure 36. *The presumed Binding mode of **3-NDI-thread** to **$P_{Stopper}$** and the model compounds **3-NDI-thread** and **DNP-fragment**.*

DSSCs were prepared with [**3-NDI-thread] =** 25, 50, 100, and 200 mM to study the influence on the shape of the Nyquist plot. Figure S37a shows the Nyquist plot at 100 mW cm^{-2} irradiation for the different DSSCs. At low mediator concentration (25 mM and 50 mM) indeed the emergence of the 45° line from the semicircle is visible while this is not the case at higher mediator concentrations (100 mM and 200 mM). These plots were analyzed using the same model as for the **3-NDI-ring** DSSCs (Experimental Section 2.5.). The diffusion coefficient was modeled and indeed a trend is found from faster diffusion at 200 mM to slow diffusion for 25 mM mediator. Modeling these results confirm that at low mediator concentration (≤ 50 mM)

DSSCs are limited by slow diffusion of the redox couple. The reason for this limitation is the distance that a redox mediator has to travel through the electrolyte to mediate charge.

The influence of concentration on the performance of DSSC is further investigated with **3-NDI-thread** and the **$P_{Stopper}$** dye. For this specific system the performance does not scale with increased concentration, but an optimum is found between 50 and 25 mM. The highest voltage is reached for 50 mM, while the highest current is obtained for 25 mM. Table S13 lists the difference in τ_h for different mediator concentrations. Longer hole lifetimes are reached by DSSCs employing mediator concentrations ≤ 50 mM leading to higher PCEs in this system. Despite the higher mediator concentration > 50 mM, these DSSCs perform less in terms of V_{OC}, J_{SC}, FF and PCE. We speculate this is caused by stacking of **3-NDI-thread** with itself, which is a common observed phenomenon with flat naphthalene diimide structures.[28] These stacks comprised of **3-NDI-threads** may form polymers competing with the charge carrier function of the mediator interfering with DSSC performance.

Table S13. *Summary of the photovoltaic performance data for DSSC from J–V curves based on **$P_{Stopper}$** and different concentrations of **3-NDI-thread** electrolyte in 1 M LiTFSI valerontirile/MeCN, (15:85) under AM 1.5G illumination (100 mW cm^{-2}). Diffusion coefficient hole lifetimes (τ_h) of different systems at 100 mV determined by modeling EIS measurements.*

**C (mM)**	**V_{OC} (V)**	**J_{sc} (mA cm^{-2})**	**FF**	**PCE (%)**	**τ_h (ms)**	**D_e (m^2 s^{-1})**
200	0.101	-0.11	0.257	0.003	43.9	7.61×10^{-9}
100	0.218	-0.21	0.037	0.015	60.2	5.81×10^{-10}
50	0.308	-0.16	0.379	0.019	356	5.91×10^{-10}
25	0.250	-0.23	0.360	0.020	314	2.49×10^{-10}

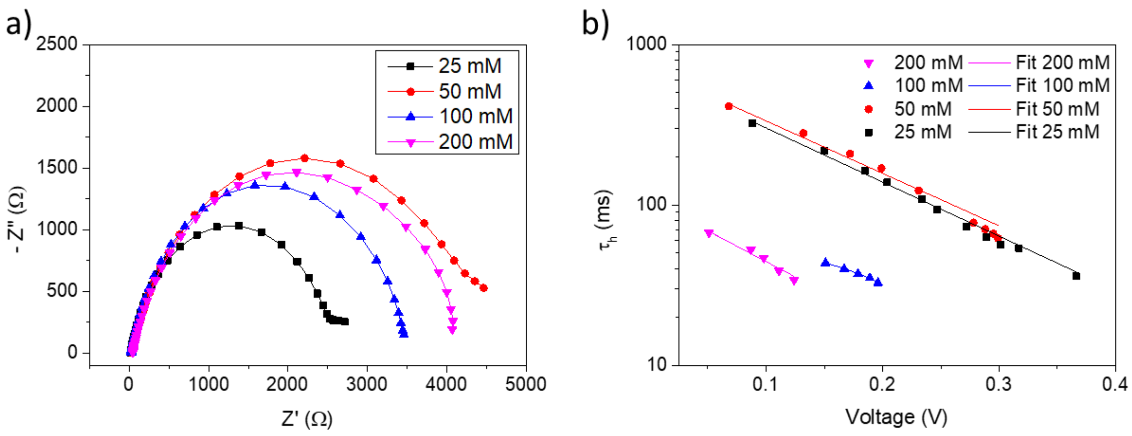

Figure S37. *EIS measurements conducted at varying light intensities measured at open voltage for* ***$P_{Stopper}$*** *DSSC employing* ***3-NDI-thread*** *electrolyte (25 mM, black squares; 50 mM, red dots; 100 mM, blue triangles; 200 mM pink triangles). a) Nyquist plot obtained at 100 mW cm^{-2} with varying concentration of* ***3-NDI-thread*** *of a semicircle that features a 45° line at low concentrations. b) Hole lifetime as a function of voltage obtained from EIS measurements for different concentrations of mediator.*

S3.12. Comparison IPCE set-up and solar simulator

IPCE is measured form 414–723 nm (resolution = 1 nm) in continuous mode. If we transform the IPCE spectrum of **P1** 1 M I^-/I_3^- DSSC (which can be found in Ref [1]) to the photon flux and integrate the spectrum using a python script we can determine the photocurrent density that we can compare with the photocurrent density that we actually measure in the *J–V* curves with the solar simulator with AM 1.5G illumination (100 mW cm^{-2}). As these values for the photocurrent density are very similar (Table S14), we assume that the IPCE measured for all DSSC under study match the outcome of the *J–V* curves.

Table S14. Integrated photocurrent density form IPCE measurement versus photocurrent density measured by J–V-curve with AM 1.5G illumination (100 mW cm^{-2}).

**Method**	**Measured photocurrent density (mA cm^{-2})**
Integrated photocurrent	2.10
J–V curve	1.97

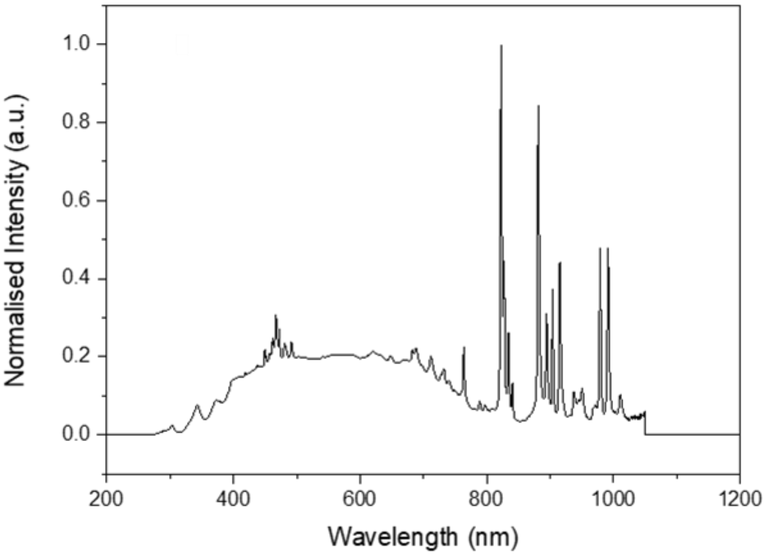

Figure S38. Spectrum solar simulator used in this study Oriel LCS-100 solar simulator.

S4. References

[1] T. Bouwens, T. M. A. Bakker, K. Zhu, J. Hasenack, M. Dieperink, A. M. Brouwer, A. Huijser, S. Mathew, J. N. H. Reek, *Nat Chem* **2023**, *15*, 213–221.

[2] C. L. Brown, D. Philp, N. Spencer, J. Fraser Stoddart, *Isr J Chem* **1992**, *32*, 61–67.

[3] P. Qin, H. J. Zhu, T. Edvinsson, G. Boschloo, A. Hagfeldt, L. C. Sun, *J Am Chem Soc* **2008**, *130*, 8570–8571.

[4] L. Steemers, M. J. Wanner, A. W. Ehlers, H. Hiemstra, J. H. van Maarseveen, *Org Lett* **2017**, *19*, 2342–2345.

[5] V. V. Pavlishchuk, A. W. Addison, *Inorganica Chim Acta* **2000**, *298*, 97–102.

[6] N. G. Connelly, W. E. Geiger, *Chem Rev* **1996**, *96*, 877–910.

[7] O. Š. Miljanić, W. R. Dichtel, S. I. Khan, S. Mortezaei, J. R. Heath, J. F. Stoddart, *J Am Chem Soc* **2007**, *129*, 8236–8246.

[8] X. Wang, S. S. Nurttila, W. I. Dzik, R. Becker, J. Rodgers, J. N. H. Reek, *Chem. Eur. J.* **2017**, *23*, 14769–14777.

[9] K. Zhu, S. K. Frehan, A. M. Jaros, D. B. O'Neill, J. P. Korterik, K. Wenderich, G. Mul, A. Huijser, *The Journal of Physical Chemistry C* **2021**, *125*, 16049–16058.

[10] J. J. Snellenburg, S. P. Laptenok, R. Seger, K. M. Mullen, I. H. M. van Stokkum, *J Stat Softw* **2012**, *49*, DOI 10.18637/jss.v049.i03.

[11] P. Qin, J. Wiberg, E. A. Gibson, M. Linder, L. Li, T. Brinck, A. Hagfeldt, B. Albinsson, L. Sun, *The Journal of Physical Chemistry C* **2010**, *114*, 4738–4748.

[12] K. Zhu, S. K. Frehan, G. Mul, A. Huijser, *J Am Chem Soc* **2022**, *144*, 11010–11018.

[13] U. B. Cappel, S. M. Feldt, J. Schöneboom, A. Hagfeldt, G. Boschloo, *J Am Chem Soc* **2010**, *132*, 9096–9101.

[14] X. Chen, H. Chen, J. Fraser Stoddart, *Angewandte Chemie International Edition* **2023**, *62*, DOI 10.1002/anie.202211387.

[15] S. A. Vignon, J. F. Stoddart, *Collect Czechoslov Chem Commun* **2005**, *70*, 1493–1576.

[16] E. A. Neal, S. M. Goldup, *Chem. Commun.* **2014**, *50*, 5128–5142.

[17] A. Hagfeldt, G. Boschloo, L. Sun, L. Kloo, H. Pettersson, *Chem. Rev.* **2010**, *110*, 6595–6663.

[18] B. Pashaei, H. Shahroosvand, P. Abbasi, *RSC Adv* **2015**, *5*, 94814–94848.

[19] J. Halme, P. Vahermaa, K. Miettunen, P. Lund, *Advanced Materials* **2010**, *22*, E210–E234.

[20] Z. Huang, G. Natu, Z. Ji, P. Hasin, Y. Wu, *The Journal of Physical Chemistry C* **2011**, *115*, 25109–25114.

[21] I. Hod, Z. Tachan, M. Shalom, A. Zaban, *Physical Chemistry Chemical Physics* **2013**, *15*, 6339.

[22] L. Favereau, Y. Pellegrin, L. Hirsch, A. Renaud, A. Planchat, E. Blart, G. Louarn, L. Cario, S. Jobic, M. Boujtita, F. Odobel, *Adv Energy Mater* **2017**, *7*, 1601776.

[23] Z. Liu, W. Li, S. Topa, X. Xu, X. Zeng, Z. Zhao, M. Wang, W. Chen, F. Wang, Y. B. Cheng, H. He, *ACS Appl Mater Interfaces* **2014**, *6*, 10614–10622.

[24] E. Benazzi, J. Mallows, G. H. Summers, F. A. Black, E. A. Gibson, *J Mater Chem C Mater* **2019**, *7*, 10409–10445.

[25] F. Fabregat-Santiago, G. Garcia-Belmonte, I. Mora-Seró, J. Bisquert, *Physical Chemistry Chemical Physics* **2011**, *13*, 9083.

[26] F. Fabregat-Santiago, J. Bisquert, E. Palomares, S. A. Haque, J. R. Durrant, *J Appl Phys* **2006**, *100*, 034510.

[27] R. D. Shannon, *Acta Crystallographica Section A* **1976**, *32*, 751–767.

[28] J. Leira-Iglesias, A. Tassoni, T. Adachi, M. Stich, T. M. Hermans, *Nat Nanotechnol* **2018**, *13*, 1021–1027.
